# About the dark corners in the gene function space of *Escherichia coli* remaining without illumination by scientific literature

**DOI:** 10.1186/s13062-023-00362-0

**Published:** 2023-02-28

**Authors:** Erwin Tantoso, Birgit Eisenhaber, Swati Sinha, Lars Juhl Jensen, Frank Eisenhaber

**Affiliations:** 1 Agency for Science, Technology and Research (A*STAR), Genome Institute of Singapore (GIS), 60 Biopolis Street, Singapore, 138672 Republic of Singapore; 2 Agency for Science, Technology and Research (A*STAR), Bioinformatics Institute (BII), 30 Biopolis Street #07-01, Matrix Building, Singapore, 138671 Republic of Singapore; 3 Novo Nordisk Foundation Center for Protein Research, Faculty of Health and Medical Sciences, University of Copenhagen, Copenhagen, Denmark; 4 School of Biological Sciences, Nanyang Technological University, 60 Nanyang Drive, Singapore, 637551 Republic of Singapore; 5 Present Address: European Bioinformatics Institute (EMBL-EBI), Wellcome Genome Campus, Hinxton, Cambridge, CB10 1SD UK

**Keywords:** *Escherichia coli*, Gene function space, Uncharacterized genes, Gene function discovery rate, Protein function, *yahV*, *yddL*, Cryptic prophage

## Abstract

**Background:**

Although *Escherichia coli* (*E. coli*) is the most studied prokaryote organism in the history of life sciences, many molecular mechanisms and gene functions encoded in its genome remain to be discovered. This work aims at quantifying the illumination of the *E. coli* gene function space by the scientific literature and how close we are towards the goal of a complete list of *E. coli* gene functions.

**Results:**

The scientific literature about *E. coli* protein-coding genes has been mapped onto the genome via the mentioning of names for genomic regions in scientific articles both for the case of the strain K-12 MG1655 as well as for the 95%-threshold softcore genome of 1324 *E. coli* strains with known complete genome. The article match was quantified with the ratio of a given gene name’s occurrence to the mentioning of any gene names in the paper. The various genome regions have an extremely uneven literature coverage. A group of elite genes with ≥ 100 full publication equivalents (FPEs, FPE = 1 is an idealized publication devoted to just a single gene) attracts the lion share of the papers. For K-12, ~ 65% of the literature covers just 342 elite genes; for the softcore genome, ~ 68% of the FPEs is about only 342 elite gene families (GFs). We also find that most genes/GFs have at least one mentioning in a dedicated scientific article (with the exception of at least 137 protein-coding transcripts for K-12 and 26 GFs from the softcore genome). Whereas the literature growth rates were highest for uncharacterized or understudied genes until 2005–2010 compared with other groups of genes, they became negative thereafter. At the same time, literature for anyhow well-studied genes started to grow explosively with threshold T10 (≥ 10 FPEs). Typically, a body of ~ 20 actual articles generated over ~ 15 years of research effort was necessary to reach T10. Lineage-specific co-occurrence analysis of genes belonging to the accessory genome of *E. coli* together with genomic co-localization and sequence-analytic exploration hints previously completely uncharacterized genes *yahV* and *yddL* being associated with osmotic stress response/motility mechanisms.

**Conclusion:**

If the numbers of scientific articles about uncharacterized and understudied genes remain at least at present levels, full gene function lists for the strain K-12 MG1655 and the *E. coli* softcore genome are in reach within the next 25–30 years. Once the literature body for a gene crosses 10 FPEs, most of the critical fundamental research risk appears overcome and steady incremental research becomes possible.

**Supplementary Information:**

The online version contains supplementary material available at 10.1186/s13062-023-00362-0.

## Introduction

The *E. coli* species is the most extensively studied prokaryote model organisms. It is not only a laboratory/biotechnology workhorse, a commensal gut bacterium or a pathogen in animal/human health [1–5], *E. coli* continues to colonize new secondary habitats [6–8].

With the K-12 genome completely sequenced in 1997 [9], the previously unprecedented but not yet materialized opportunity appeared to fully understand this prokaryote organism’s molecular mechanisms at a system-wide level. About 25 years later, thousands of *E. coli* genomes and other omics datasets are available in public databases. Comprehensive resources on *E. coli* genes, proteins, pathways, molecular interactions, and chemical reactions have been provided by EcoCyc [10, 11] through manual curation and integration of literature and experimental evidence. Yet, pangenome analyses revealed many insufficiently uncharacterized gene groups [12–15].

Completeness of the table of molecular, cellular and phenotypic functions [16] associated with every gene is a first step to understand the total biological potential encoded in a genome. Since there is no large-scale methodology to discover myriads of individual gene functions in parallel, it requires painstakingly careful research work dedicated to the study of specific genes/transcripts/proteins and scientific papers mentioning their names in their title, abstract and/or main text. Thus, it is possible to map the scientific literature onto the genome via gene/RNA/protein names occurring in the paper. As a trend, a genome region mentioned in a larger set of articles will be functionally better understood than another one with much less literature or even with no paper hit at all.

Thus, gene/RNA/protein names link the scientific literature with genome regions. The article match can be quantified with the ratio of a given gene name’s occurrence to the total mentioning of any gene names in the paper. We defined the full publication equivalent (FPE) as an idealized paper that is dedicated towards the function of one gene/protein only [17]. Typically, every paper talks about several genes/proteins concurrently. So, such a paper adds a fractional count towards the literature score of that gene/protein depending on how often it has been mentioned (Methods).

A similar exercise with the human genome in 2018 showed that it is very unevenly illuminated by scientific articles [17]. Whereas almost 95% of the respective literature describes an elite group of ~ 4800 protein-coding genes, another ~ 7000 genes are talked about in less than 0.5% of the articles. About 4000 human protein-coding genes are not mentioned in any scientific publication at all.

More surprisingly, until about the year 2000, the fastest growing groups of human genes in the newly added literature were those that have never/rarely been reported about in previous years. This optimistic trend culminated with ~ 550 new gene function discoveries in 2000. Thereafter, research on previously uncharacterized genes essentially collapsed and the fastest growing group of genes in the newly added literature was those with hundreds of articles about them published previously. This trend is especially remarkable at the background of the explosive growth of biomedical literature: The total number of papers published until 2000 is about the same as thereafter (10.7 million entries in 2000 and 24.3 million in 2017 in PUBMED [17, 18]).

Besides analyzing genomes of specific *E. coli* strains such as that of K-12 MG1655, we need a list of genes for this research effort that characterizes *E. coli* as a species. Comparisons of available complete genomes shows that the pool of homologous gene families (GFs) shared by all strains of *E. coli* is very small (a few hundred) [15]. At the same time, the *E. coli* pangenome is open and grows with the sophistication of ever cheaper sequencing technology and the entry of genomes especially from strains in new habitats [8, 15]. Undoubtedly, the accessory gene pool will keep increasing as more *E. coli* genomes are accumulated. On the contrary, the softcore genome (at the threshold of 92% or 95% of all genomes) is stable regardless of the addition of new genomes [15] and does provide the lists of gene families that is critical for our purpose.

In this work, we quantify the extent of illumination of the gene function space of *E. coli* (K-12 MG1655 strain’s genes and softcore genome gene families (GFs)) by the scientific literature. Where are the genome regions that would deserve enhanced scientific attention and promise new discovery? Subsequently, we search for functional hints for some of the so-called enigmatic genes—using co-occurrence analysis of genes of the accessory genome [15, 19] among the lineages of *E. coli*, genomic co-localization as well as traditional gene/protein sequence analysis.

## Results

### Current status of the *E. coli* gene function space coverage by the scientific literature

One lakh seventy one thousand five hundred and ninety PubMed publications (= total number of FPEs) attributable to the genes of *E. coli* K-12 MG1655 as of June 8th, 2022, mention 4097 out of the 4273 unique genes (Table 1, Additional file 3: Files 1 and 2) in our automated mapping procedure (Methods). The remaining 176 cases were tested by manual literature searches (Additional file 1: Table S1). Indeed, we found at least one specifically committed scientific article for 31 (for 8 of them, two or more). This finding illustrates that the rule set for the automated literature assignment procedure is rather underestimating FPE scores for the sake of suppressing false-positive gene-publication links. Thus, at least 145 genes of K-12 MG1655 do not have a single directly dedicated scientific article.

**Table 1:** The number of *E. coli* K-12 genes as well as sum of literature score in various FPE score ranges

FPE score range	#Genes	Percentage of 4273 genes (%)	Literature score	Percentage of total score (%)	ƩGenes	Category
0	176	4.12	0.00	0.00	176	Not studied
0 < x < 1	692	16.19	231.95	0.14	2190	Very understudied
1 ≤ x < 5	967	22.63	2484.55	1.45		
5 ≤ x < 10	531	12.43	3896.38	2.27		
10 ≤ x < 15	322	7.54	3980.72	2.32	719	Understudied
15 ≤ x < 20	221	5.17	3833.54	2.23		
20 ≤ x < 25	176	4.12	3945.99	2.30		
25 ≤ x < 30	153	3.58	4202.54	2.45	372	Moderately studied
30 ≤ x < 35	112	2.62	3639.35	2.12		
35 ≤ x < 40	107	2.50	3962.86	2.31		
40 ≤ x < 45	65	1.52	2758.66	1.61	357	Intensively studied
45 ≤ x < 50	72	1.68	3440.15	2.00		
50 ≤ x < 75	220	5.15	13,442.13	7.83		
75 ≤ x < 100	117	2.74	10,031.58	5.85	459	Very intensively studied
100 ≤ x < 500	299	7.00	56,145.26	32.72		
x ≥ 500	43	1.01	55,594.34	32.40		
Total	4273	–	171,590	–	4273	–

This table presents the results of the automated mapping of publications onto the K-12 MG1655 genome. We list the total number of genes in the respective FPE range at the time of study (“#Genes”). We added a row for the 176 genes not specifically mentioned in any article about *E. coli.* Also, we calculated the sum of the literature score for all genes in the respective FPE range (“Literature Score”). The total literature score is equivalent to the total number of articles identified in this study. The FPE score range is further classified into six categories and the total number of genes in that category is provided (“ΣGenes”)

The literature coverage for the gene function space of *E. coli* K-12 MG1655 is very uneven (Table 1). An elite group of 342 genes (~ 8% of all genes) with FPE-score ≥ 100 is claimed by about two thirds of all articles (65.12% of all FPEs). Many of the genes/proteins with FPE-score ≥ 500 (listed in Additional file 1: Table S2) are involved in cell division, basic metabolism and transport as well as in pathogenesis. In some cases, the gene is mentioned so often since it has been used as standardized laboratory tool for genetic/cellular engineering (e.g., the top hit b0344/β-galactosidase). At the same time, 2366 genes (> 55% of all genes) with an FPE-score < 10 are mentioned in only 3.86% of the relevant literature.

Separately, we investigated the literature coverage of the 3056 GFs belonging to the 95%-threshold *E. coli* softcore genome [15, 20]. We mapped literature data about genes belonging to six strains (Additional file 1: Table S3) to the softcore GFs. 174,120 articles mention at least one gene from those strains (Additional file 3: Files 1B and 2B). Notably, more than 98.5% of the relevant publications map to the laboratory model of *E. coli* K-12 MG1655. In fact, the K-12 relevant literature covers 99.8% of the softcore genome GFs that have been automatically annotated with publications by our procedure; thus, the other papers exclusively dedicated to genes from the other five strains add little new.

The softcore genome literature coverage of the *E. coli* resembles trends observed for K-12 (Additional file 1: Table S4). 160,598 publications mention genes/proteins included into any of the GFs that are part of softcore genome. Not a single article is automatically mapped for 39 GFs. Manual checks reveal 26 GFs having indeed no literature (legend to Additional file 1: Table S1). The total number of GFs with an FPE-score < 10 is 1347 (~ 44.08% of all GFs) but their share in the literature is miserable ~ 2.82%.

At the same time, more than two thirds (~ 67.72%) of all relevant publications describe the functions of just 342 elite GFs (with ≥ 100 FPEs) or just about 11.2% of the total softcore genome. Despite the funny numerical coincidence with the 342 elite genes from K-12 MG1655, these two sets are just largely overlapping but not identical. The common set consists of 310 GFs involving 313 *E. coli* K-12 MG1655 genes.

The following 29 genes in *E. coli* K-12 are intensively studied but they are not part of the softcore genome: b0294 (*ecpR*/GF_4060), b0343 (*lacY*/GF_8460), b0351 (*mhpF*/GF_4282), b0533 (*sfmH*/GF_11601), b0555 (*rrrD*/GF_380), b0557 (*borD*/GF_2868), b0565 (*ompT*/GF_499), b1159 (*mcrA*/GF_8991), b1182 (*hlyE*/GF_773), b1554 (*rrrQ*/GF_155), b1563 (*relE*/GF_500), b1617 (*uidA*/GF_3547), b1923 (*fliC*/GF_4133), b2000 (*flu*/GF_24343), b2027 (*wzzB*/GF_871), b2233 (*yfaL*/GF_2033), b2269 (*elaD*/GF_1434), b2592 (*clpB*/GF_10360), b2731 (*fhlA*/GF_9616), b2741 (*rpoS*/GF_6615), b2758 (*casC/*GF_9628), b2782 (*mazF*/GF_2500), b3501 (*arsR*/GF_510), b3531 (*bcsZ*/GF_9943), b3717 (*cbrC*/GF_3491), b4011 (*yjaA*/GF_20677), b4031 (*xylE*/GF_10135), b4348 (*hsdS*/GF_3705), and b4351 (*mrr*/GF_10255).

Similarly, 32 intensively studied GFs from the softcore genome do not contain an *E. coli* K-12 MG1655 homologue: GF_1621 (*tsr*), GF_2450 (*hupB*), GF_4083 (*dacA*), GF_4087 (*tdcE*), GF_6560 (*lpxP*), GF_7816 (*gadA*), GF_8530 (*ddlB*), GF_8575 (*fabZ*), GF_8688 (*copA*), GF_8883 (*mukB*), GF_9300 (*msrP*), GF_9561 (*pheA*), GF_9739 (*qseC*), GF_9929 (*uspA*), GF_9955 (*malS*), GF_10021 (*atpD*), GF_10037 (*wzxE*), GF_10051 (*tatA*), GF_10139 (*malK*), GF_10394 (*arcB*), GF_10447 (*cysK*), GF_11186 (*crr*), GF_12120 (*ptrB*), GF_18493 (*aroF*), GF_24466 (*proP*), GF_24602 (*acrB*), GF_27107 (*csgD*), GF_28093 (*mtr*), GF_29670 (*clpB*), GF_29701 (*argP*), GF_29714 (*ruvB*), GF_29740 (*glnB*).

### Changes with time of the *E. coli* gene function space coverage by the scientific literature

Clearly, the uneven illumination of the *E. coli* gene function space by the scientific literature is unsatisfactory. Yet, what are the past and recent trends in gene/protein function discovery for *E. coli* and is there a justified hope for a principal change of the status towards a full list of gene functions?

**Fig. 1:**
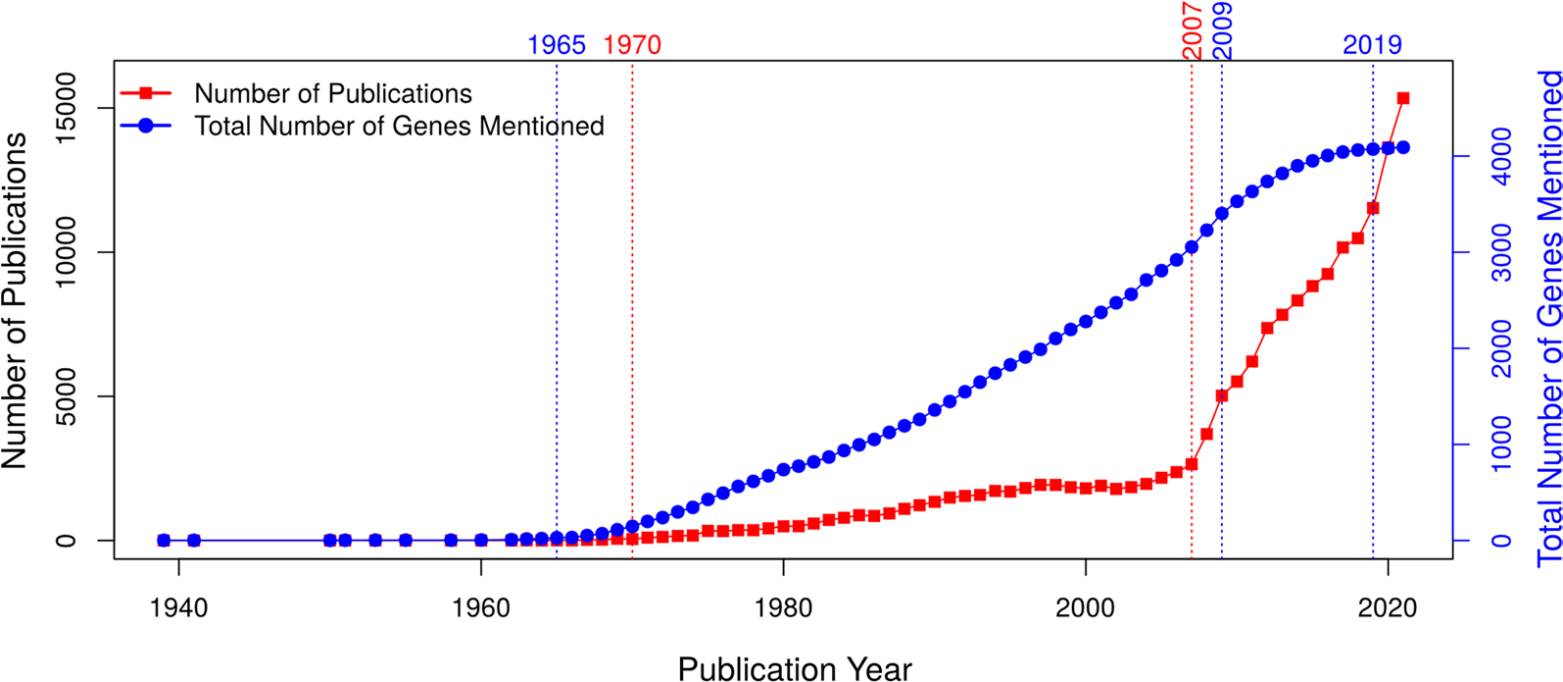
The total number of genes that has been mentioned in relation to the total number publications with *E. coli* K-12 MG1655 gene names from year 1939 to year 2021. The number of publications (left y-axis) for each year is represented by the red line, whereas the total number of genes mentioned (right y-axis) is shown by the blue line. The blue dashed vertical lines delimit the expansion period for the total number of genes from year 1965 to 2009 until it apparently plateaued after year 2019. The red dashed vertical lines at year 1970 and year 2007 emphasize the two publication periods from year 1970 to year 2007 and year 2007 to year 2021. The ratio of the number of publications in each year to the total number of new genes identified in each year is shown in the inset

Figure 1 shows the annual total number of dedicated publications mentioning *E. coli* K-12 MG1655 genes/proteins together with the total number of genes that have already been mentioned up to (and including) the year of study (Additional file 3: Files 3 and 4).

The growth of the number of genes mentioned approximately follows an S-curve. An infancy period prior to 1965 is followed by an expansion 1965–2009 and a saturation thereafter. An apparent plateau happened around year 2019 where the total number of genes mentioned has reached ~ 95% of the total (4073 genes out of 4273 genes). Indeed, the number of genes not mentioned at all has become quite small and the remaining set might represent problems that are increasingly difficult to crack. Thus, the discovery of function for yet uncharacterized K-12 genes has essentially stalled.

The publication dynamics seems completely dissociated. Moderate growth during 1970–2007 (slope = 66.3; *P*-value < 10^–15^) changes into explosive growth during 2007–2021 (slope = 776.1; *P*-value < 10^–10^). As a result, the ratio of new literature items to the total number of new genes skyrockets after year 2009 (insert of Fig. 1), indicating a considerable dilution of new content.

The softcore genome’s literature coverage exhibits the same trends (Additional files 2 and 3: Fig. S1 and Files 3B, 4B). The apparent plateau starting with 2019 corresponds to the total number of softcore GFs mentioned reaching ~ 98% of the total GFs (2999 out of 3056 GFs). Similarly, the publication dynamics is characterized by slow growth 1970–2007 (slope = 61.25; *P*-value < 10^–15^), a phase followed by an explosive growth 2007–2021 (slope = 726.2; *P*-value < 10^–10^).

### Accelerated growth of literature coverage for well-studied genes but not for under-characterized ones in recent years reverses the historically observed opposite pattern

**Fig. 2:**
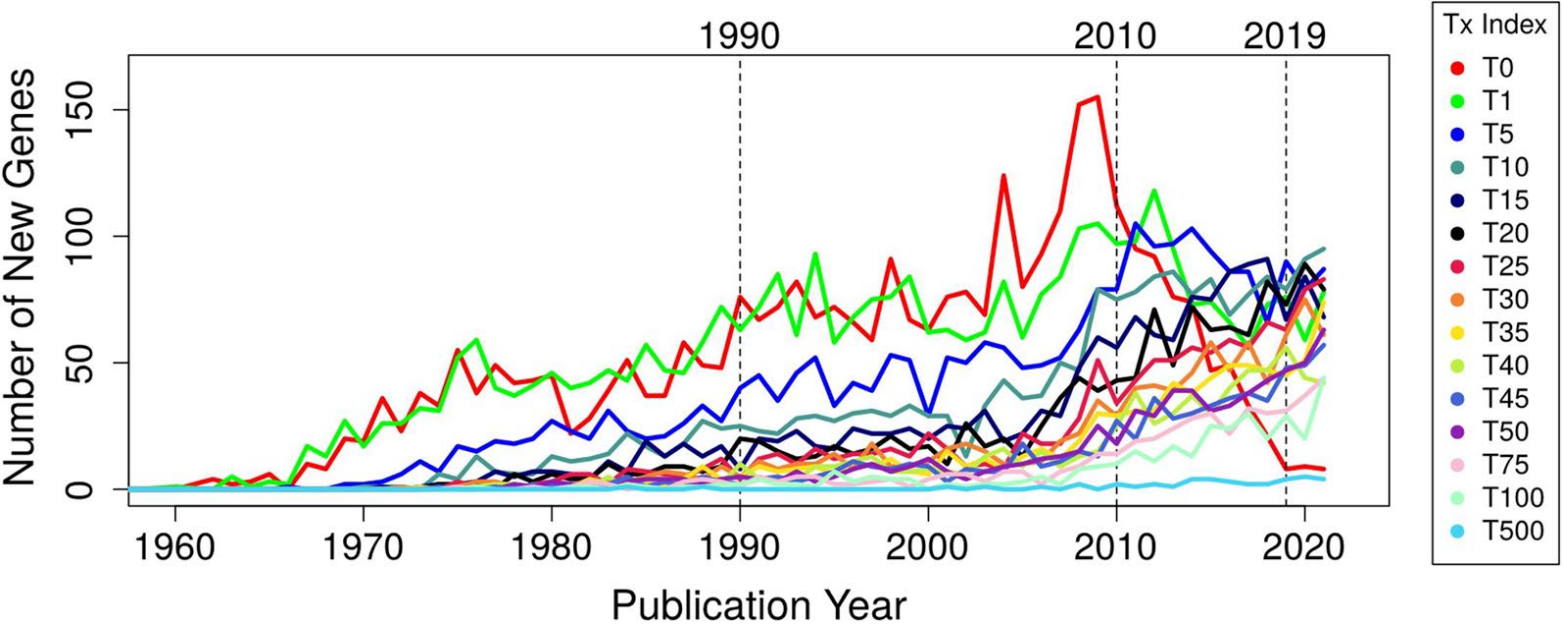
Gene function discovery rate from year 1960 to 2021 for *E. coli* K-12 MG1655. The gene function discovery rate measured as the number of new genes first mentioned (T0) or crossing a specific threshold of aggregated FPEs (T1, T5, T10, …, T50, T75, T100 and T500) from year 1960 until year 2021

Next, we break down the pool of all *E. coli* K-12 MG1655 genes with regard to FPE-ranges and analyse their annual changes with a linear regression model (Table 2, Fig. 2, Additional files 2 and 3: Figs. S2, S3 and File 5). We see that the number of genes with the publication status T0, T1 and T5 (least studied genes) grows until 2005–2010 with slopes that are higher than that of any other FPE bracket in the same time period. After ~ 2010, publication activity for uncharacterized or almost not studied genes collapses when, as evidenced by Table 1, the pool of such genes is far from exhausted. Remarkably, the number of genes with publication status T10 and better (except for T500) grew slowly until about 2005 only to have a drastically accelerated increase thereafter (with an order of magnitude enlarged slope). The data for the softcore genome exhibit similar trends (Additional files 1, 2 and 3: Table S5, Figs. S4, S5 and File 5B).

**Table 2:** The growing trend of literature coverage for *E. coli* K-12 genes in various FPE score thresholds

FPE score threshold	Phase 1					Phase 2				
	Years	Slope	R^2^	ρ	*P*-value	Years	Slope	R^2^	ρ	*P*-value
0	–	–	–	–	–	–	–	–	–	–
T0 (0 < x < 1)	1960–2009 ↑	**2.28**	0.81	0.90	1.04E-18	2009–2021 ↓	**− 11.37**	0.94	0.97	3.95E-08
T1 (1 ≤ x < 5)	1965–2009 ↑	**1.68**	0.79	0.89	5.76E-16	2009–2021 ↓	**− 3.63**	0.57	0.75	2.93E-03
T5 (5 ≤ x < 10)	1970–2013 ↑	**1.78**	0.84	0.92	3.20E-18	2013–2021 ↓	**− 2.33**	0.36	0.60	9.00E-02
T10 (10 ≤ x < 15)	1973–2001 ↑	**1.08**	0.89	0.94	1.70E-14	2001–2021 ↑↑	**3.46**	0.79	0.89	7.07E-08
T15 (15 ≤ x < 20)	1973–2003 ↑	**0.85**	0.84	0.92	3.68E-12	2003–2021 ↑↑	**3.61**	0.77	0.88	7.79E-07
T20 (20 ≤ x < 25)	1973–2004 ↑	**0.65**	0.77	0.88	3.44E-11	2004–2021 ↑↑	**3.88**	0.87	0.93	1.83E-08
T25 (25 ≤ x < 30)	1975–2004 ↑	**0.49**	0.61	0.78	2.94E-07	2004–2021 ↑↑	**3.81**	0.91	0.95	8.04E-10
T30 (30 ≤ x < 35)	1975–2004 ↑	**0.51**	0.75	0.87	4.82E-10	2004–2021 ↑↑	**3.42**	0.89	0.94	5.23E-09
T35 (35 ≤ x < 40)	1975–2004 ↑	**0.41**	0.75	0.86	7.83E-10	2004–2021 ↑↑	**3.06**	0.91	0.95	1.26E-09
T40 (40 ≤ x < 45)	1975–2006 ↑	**0.41**	0.69	0.83	3.69E-09	2006–2021 ↑↑	**2.65**	0.81	0.90	2.08E-06
T45 (45 ≤ x < 50)	1975–2006 ↑	**0.36**	0.66	0.81	1.53E-08	2006–2021 ↑↑	**2.83**	0.90	0.95	1.55E-08
T50 (50 ≤ x < 75)	1975–2006 ↑	**0.32**	0.75	0.87	1.60E-10	2006–2021 ↑↑	**2.82**	0.88	0.94	6.96E-08
T75 (75 ≤ x < 100)	1980–2006 ↑	**0.16**	0.37	0.61	7.34E-04	2006–2021 ↑↑	**2.33**	0.93	0.96	2.71E-09
T100 (100 ≤ x < 500)	1980–2006 ↑	**0.11**	0.23	0.48	1.19E-02	2006–2021 ↑↑	**2.02**	0.78	0.88	6.81E-06
T500 (x ≥ 500)	1980–2021 ↑	**0.09**	0.56	0.75	1.53E-08	–	–	–	–	–

The slope is the most important information that was shown in bold

The letter “T” in abbreviations “T0, T1, etc.” stands for “threshold” applied to FPE values. Further, the curve of the number of new genes in the respective FPE range as a function of the year (see Fig. 2) is analyzed with linear regression methods. The trend of changes is generally identified through two phases, *i. e.* Phase 1 and Phase 2. The slopes, R^2^, ρ and *P*-value in time intervals of Phase 1 and Phase 2 are listed based on linear regression model *y*_*i*_ ~ *C* + *b.x*_*i*_; where *y*_*i*_ = total number of new genes reaching the specific FPE threshold at year *i*; *x*_*i*_ = year *i*; *b* is the slope and *C* is intercept. The slope (*b*) indicates the rate increase/decrease of the total number of new genes reaching a specific FPE score threshold throughout the years. A positive slope indicates that, as a trend, the total number of new genes reaching a specific FPE score threshold is larger than the previous year (or from year to year); a negative slope indicates otherwise. ρ is the linear correlation between the total number of new genes reaching a specific FPE score threshold and year. R^2^ is the square of correlation or the goodness of fit of the linear regression. *P*-value is the statistical significance of the slope. The total number of genes reaching the specific FPE score threshold can then be estimated by: *N*_*i*_ ~ *N*_*(i-1)*_ + *y*_*i*_; where *N*_*i*_ and *N*_*(i-1)*_ = total number of genes reaching the specific FPE score threshold at year *i* and *(i-1*) respectively. The symbol ↑ indicates growing trend, whereas the symbol ↓ indicates declining trend. The symbol ↑↑ indicates accelerating growth trend

Thus, research teams clearly shy away from risky work on gene function discovery whereas more incremental research on relatively well-characterized genes flourishes as never before in history. The data breakdown (both for K-12 and the softcore genome) highlights the critical role of the publication threshold T10 for research in the system *E. coli* and, possibly, for other bacterial systems of similar complexity. Apparently, the risk of project failure goes substantially down once this knowledge threshold is taken and it becomes easier to produce incremental advances with limited resources.

How many actual articles are necessary for the transition from T5 to T10? In Table 3A (comparison with human genes in Table 3B), we show how large the body of literature must be for a given gene or GF in terms of actual articles to provide for a certain FPE value. The accumulation of relevant publications occurred differently for various genes but, as a trend, we see that, for each FPE unit, almost three articles have to be published. The border between T5 and T10 is at about 20 publications. Thus, gates for enhanced publication streams about a gene open up once ~ 20 articles have already been published about it.

**Table 3:** The real number of scientific articles necessary to generate a literature body of given FPE values

FPE threshold range	Min	Max	Median	Mean	RMSD
*(A)*					
T1	1	29	3	3.82	3.27
T5	5	94	13	16.56	11.09
T10	10	146	26	32.23	19.68
T15	15	184	40	46.94	26.09
T20	20	239	53	62.21	33.46
T25	25	311	65	76.14	39.44
T30	31	363	78	89.36	44.75
T35	37	445	90	102.66	49.86
T40	42	490	100	114.83	54.91
T45	48	514	114	128.42	60.26
T50	53	475	125	140.96	64.85
T75	81	594	187	204.00	85.81
T100	113	868	243.5	267.91	113.82
T500	632	1908	1001	1102.7	339.98
*(B)*					
T1	1	57	2	4.38	5.53
T5	5	232	12	19.68	18.83
T10	10	470	26	39.02	34.94
T15	15	825	41	59.04	52.13
T20	20	1116	56	78.96	69.47
T25	25	1279	70	98.31	86.18
T30	30	1343	85	118.06	102.14
T35	35	1584	100	137.20	114.52
T40	40	1731	115	157.51	132.06
T45	45	2027	129	176.16	147.20
T50	50	2418	145	196.24	164.89
T75	75	2699	218	291.68	238.85
T100	100	2890	291	384.09	310.62
T500	502	10,167	1417	1753.57	1158.06

The letter “T” in abbreviations “T0, T1, etc.” stands for “threshold” applied to FPE values (see Table 2). We list the minimal (Min), maximal (Max), median and mean (together with the respective standard deviation—SD) numbers of articles associated with genes in the year when they crossed certain literature thresholds. As a trend, the number of actual articles is 2–3 times larger than the FPE value itself

Table 3 (A) shows the results for the E.coli literature whereas, (B) presents the data for the body of literature about human genes. Regardless of the taxon, we see similar trends with regard to number of actual publications for a given FPE range

In Table 4A (comparison with human genes in Table 4B), we present data about how many years go by in average for a gene to achieve certain FPE thresholds. Clearly, this period will depend on the availability of adequate research technologies. For example for b03 44 (β-galactosidase), we recorded the first paper from 1939 (and the T500 threshold was reached ~ 80 years later). T10 is typically achieved after 15–20 years and it takes about 30 years to get to T100. For genes first analyzed 1995 and later when DNA sequencing, mass spectrometry, etc. were widely available, it still takes much more than a decade (about 15 years) to get to T10 and about 20 years to T100.

**Table 4:** Years necessary to generate a literature body of given FPE values

T	All genes					Genes with T0 in 1995 and later				
	Min	Max	Mean	Median	SD	Min	Max	Mean	Median	SD
*(A)*										
T1	0	40	5.72	4	6.48	0	25	5.77	5	5.41
T5	1	56	15.55	14	9.21	1	27	12.26	12	5.44
T10	2	54	20.32	19	10.19	2	26	14.59	14	5.26
T15	2	57	23.03	22	10.49	3	27	15.70	16	5.04
T20	3	58	25.03	24	10.61	3	27	16.88	17	5.09
T25	3	60	26.49	26	10.86	4	27	17.12	17	4.80
T30	4	65	27.74	27	11.01	5	26	17.48	17	4.78
T35	4	76	28.72	29	11.13	6	26	17.91	18	4.73
T40	4	79	29.34	29	11.12	6	27	18.25	19	4.76
T45	5	82	30.15	30	11.24	7	27	18.74	18.5	4.78
T50	6	70	30.84	31	11.26	7	26	18.53	19	4.47
T75	7	64	32.58	33	11.27	8	26	18.94	19	4.52
T100	8	67	33.43	33	10.94	10	25	18.73	19	4.04
T500	16	54	39.56	41	8.38	25	25	25.00	25	NA

T	Genes with T0 in 1980 and later					Genes with T0 in 2000 and later				
	Min	Max	Mean	Median	SD	Min	Max	Mean	Median	SD
*(B)*										
T1	0	36	3.95	2	4.89	0	21	5.09	4	4.42
T5	0	42	11.01	10	6.65	0	22	10.13	10	4.45
T10	0	41	14.05	13	7.34	1	22	11.98	12	4.45
T15	1	42	15.89	15	7.71	1	22	12.96	13	4.42
T20	1	41	17.11	17	7.94	1	22	13.42	14	4.39
T25	1	42	17.99	18	8.08	1	22	13.80	14	4.40
T30	2	42	18.71	18	8.18	2	22	13.97	14	4.32
T35	2	42	19.25	19	8.23	2	22	14.21	15	4.32
T40	2	41	19.80	20	8.34	2	22	14.32	15	4.29
T45	2	42	20.15	20	8.38	2	22	14.33	15	4.27
T50	2	42	20.50	20	8.38	2	22	14.42	15	4.22
T75	2	42	21.69	22	8.50	3	22	14.96	15	4.23
T100	2	42	22.46	22	8.53	4	22	15.16	15	4.14
T500	6	42	25.38	26	7.84	8	22	15.62	17	4.52

The letter “T” in abbreviations “T0, T1, etc.” stands for “threshold” applied to FPE values (see Table 2). We list the minimal (Min), maximal (Max), median and mean (together with the respective standard deviation—SD) numbers of years needed to accumulate the necessary FPEs for a given gene relative to the gene’s year for T0

Surprisingly, the data for the number of years necessary to generate a body of literature that corresponds to a given FPE bracket is quite similar for *E. coli* and for human genes despite the human system being so much more complex (and the research being correspondingly more expensive for the human systems). Also, we see the disproportionally large decrease of the number of years to achieve T75, T100, and T500 compared with lower FPE score ranges for both taxa in recent years. As a trend, the research progress is faster in human than in *E. coli* despite the latter being the much simpler system with still many gene functions to discover

(A) As the research technology has dramatically changed compared with the time when the first genes achieved T0 (the first recorded publication for b0344 is from 1939), we present the data for all eligible K-12 MG1655 genes as well as separately for those with their T0 event 1995 and later. The years necessary for getting into higher T ranges get smaller for more recently studied genes but still remain clearly above a decade. Notably, the median number of years needed to make a threshold dropped more dramatically for higher literature thresholds (> 14 years for T75, T100 and T500 versus a drop by just 5–7 years for T10, T15 and T20)

(B) Similar data for the body of literature for human genes with their T0 event 1980 (or later) and, separately, for those with first mentioning in 2000 (or later). The years necessary for getting into higher T ranges get smaller for more recently studied genes but still remain clearly above a decade. Notably, the median number of years needed to make a threshold dropped more dramatically for higher literature thresholds (> 7 years for T75, T100 and T500 versus a drop by just 1–3 years for T10, T15 and T20)

### COG functional code distribution shows a stark difference in categories for the genes in very intensively studied and not well studied genes

**Fig. 3:**
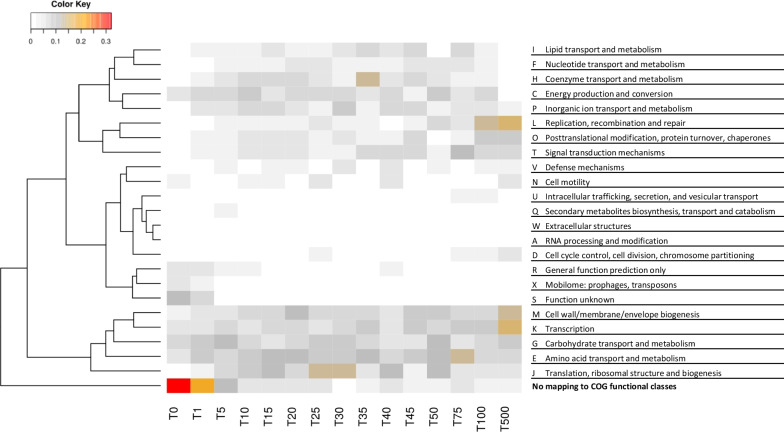
The heatmap profile of genes’ COG functions in each FPE score range. The heatmap coloring represents the percentage of genes in each COG category for each FPE score range (i.e., sum of the percentages for each column is one). Thus, the respective *E. coli* genes in each FPE score range are identified first. Then, they are categorized to the COGs according to the COG database sequence models and, subsequently, the COG functional code is associated with the gene. The genes without mapped COG function are classified as “UNMAPPED”. The heatmap shows the ratio of the number of genes with a given COG functional code in a given FPE score range to the total number of genes within that particular FPE score range. The color ranges from white (very low), grey (low), orange (high) to red (very high). The highly studied genes are overrepresented in the functional code M (cell wall/membrane/envelope biogenesis), K (transcription) and L (replication, recombination and repair). This is probably expected as it is related to bacterial pathogenesis and replication. The understudied genes are overrepresented in the function code Q (secondary metabolites biosynthesis, transport and catabolism), W (extracellular structures), A (RNA processing and modification), X (mobilome), and U (intracellular trafficking, secretion and vesicular transport). This suggests that there are still plenty of opportunity in the study of bacteria's secondary metabolites as well as mobilome-related functions

We mapped the gene IDs for the K-12 strain onto the NCBI COG reference database (possible for 3542 out of 4273 genes). We calculated the ratio of the number of genes with a given COG functional code and within a given FPE score range (using the T-coding as in Table 2) to the total number of genes within that particular FPE score range (shown as heatmap in Fig. 3). An unsupervised hierarchical clustering was performed on the functional code level while preserving the order of the FPE score ranges.

The very highly studied genes are overrepresented in codes M (cell wall/membrane/envelope biogenesis), K (transcription) and L (replication, recombination, and repair). There is also a substantial number of genes with codes E (amino acid transport and metabolism) and G (carbohydrate transport and metabolism) for the very highly studied genes.

Codes Q (secondary metabolites biosynthesis, transport, and catabolism), W (extracellular structures), A (RNA processing and modification), X (mobilome: prophages, transposons) and U (intracellular trafficking, secretion, and vesicular transport) are under-represented in all FPE score ranges. Major breakthroughs in function discoveries are to be expected for these genes.

### Coincidence associative analysis based on the pangenome matrix is used to infer the potential biological process for uncharacterized genes

**Fig. 4:**
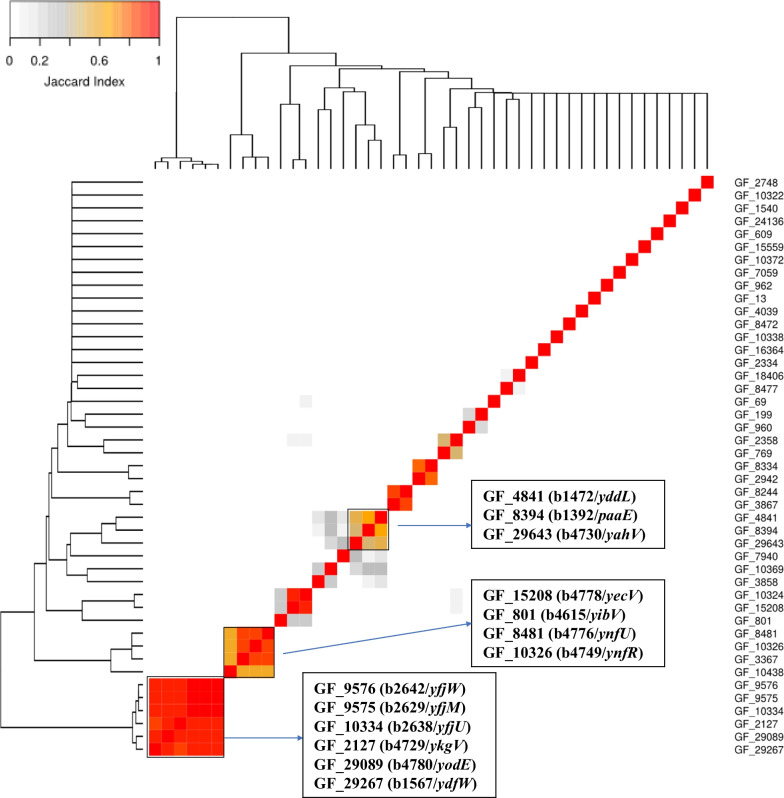
Heatmap of pairwise Jaccard Index among the 45 GFs with significantly associated GFs. The Jaccard Index represents the extend of overlap between the associated GFs. Higher Jaccard Index (closer to 1 or in red color) means higher overlap of the associated GFs between two GFs, which could suggest the two GFs could be closely related. We highlight three clusters with at least three GFs sharing common associated GFs. These clusters contain GF_29643[b4730/*yahV*], GF_4841 [b1472/*yddL*] and GF_8394 [b1392/*paaE*] (cluster I), GF_15208 [b4778/*yecV*], GF_801 [b4615/*yibV*], GF_8481 [b4776/*ynfU*] and GF_10326 [b4749/*ynfR*] (cluster II) and GF_9576 [b2642/*yfjW*], GF_9575 [b2629/*yfjM*], GF_10334 [b2638/*yfjU*], GF_2127 [b4729/*ykgV*], GF_29089 [b4780/*yodE*] and GF_29267 [b1567/*ydfW*] (cluster III). The cluster II genes belong to Qin and rac cryptic prophages, whereas cluster III genes are known to be part of cryptic prophages CP4-57 and CP4-6. Cryptic prophages have been suggested to play an important role in bacterial cell physiology including bacterial cell growth, biofilm formation and environmental stress resistance [60]

The list of 176 *E. coli* K-12 MG1655 genes without any automatically assigned literature was mapped onto 171 GFs from the pan-genome [15] (Additional file 1: Table S6). GFs with an automatically mapped K-12 publication via the homologues have been excluded (11 cases). Among the remaining 160 GFs (Additional file 3: File 6), 36 GFs belong to the 95%-threshold softcore genome. Next, we investigated if any of the 124 remaining GFs co-occur with statistical significance (*P*-value ≤ 10^–20^) together with any of the GFs in the accessory genome [15, 19] in restricted lineage ranges of *E. coli*. We find that 45 GFs have at least one significantly associated GF (Additional file 3: File 7) and, for some of these GFs, there is a substantial overlap among their associated GF lists. Three clusters with at least three GFs sharing common associated GFs are highlighted (Fig. 4). Two of them (clusters II and III) are associated with cryptic prophages.

### Manual annotation of the GFs in cluster I (GF_29643, GF_4841 and GF_8394) suggests the potential biological processes

Gene *yahV* gene (GF_29643) codes for a short bitopic protein (24 AA) with a transmembrane (TM) helix in the region 4–23 (Additional file 2: Fig. S6) [21, 22]. Many important biological processes [23–28] such as transport, modulating signal transduction, and stress response utilize small TM proteins [29–31]. Reportedly, *yahV* expression is higher during the exponential growth phase compared to the stationary phase [32].

Notably, *yahV* is co-localized with the betABIT operon (upstream) and *pdeL* gene (downstream) and this arrangement is conserved across *E. coli* genomes that harbour the *yahV* gene (Additional file 2: Figs. S7 and S8). This homologous gene cluster is specific to the majority of *E. coli* strains in phylogroups A, B1 (non-shiga) and C. The betABIT operon is expressed only under aerobic condition during osmotic stress for the production of osmoprotectants [33]. *pdeL* appears involved in the regulation of cell motility [34].

Gene *yddL* (GF_4841) codes for a protein with 96 AA with structural homology to outer membrane β-barrel proteins (e.g., osmoporin 2J1N, *P* < 10^–12^ with HHPRED [35, 36] in ANNOTATOR [21, 22]). Also, BetAware-Deep [37] predicts a four TM β-strand outer-membrane protein (*P* = 0.93, Additional file 2: Fig. S9). Annotations for *yddL* in databases are conflicting: pseudogene in the EcoGene 3.0 database [38], putative uncharacterized lipoprotein (GenBank accession UUN72560) [39] and as outer membrane β-barrel protein (GenBank accession CAD6007832, apparently via sequence homology).

Gene *paaE* (GF_8394), though left without automatically assigned literature, is actually characterized as β-ketoadipyl CoA thiolase (Additional file 1: Table S1) [40]. It is a member of the *paa* operon critical for phenylacetate catabolism [41] under aerobic condition; thus, it helps mobilizing phenylacetate and other aromatic compounds as the source of carbon and energy.

The cluster of these 3 GFs together with their 65 significantly associated GFs (Additional file 2: Fig. S10) has the most literature mapped compared with other clusters. 55 have at least one assigned article. GF_29643, GF_4841, GF_8394 and 27 other GFs are fully pairwise associated (Additional file 2: Fig. S11).

Additional file 3: File 8 shows the manual annotations of the 30 mutually associated GFs (including GF_29643, GF_4841 and GF_8394). To note, there is one GF (GF_21565), which is not present in the MG1655 strain; however, this GF exists in other strains of *E. coli* K-12. We also included *tynA* GF (from the accessory genome; sub-significantly associated to our gene list) and *paaJ* and *paaY* (GFs from the softcore genome). This summary suggests four potentially related biological processes involving *yahV* and *yddL*:

1. Osmotic regulation (*ybfB* due to its location near to *kdpFABC* operon, *ycgY* due to its location next to *treA* gene, *yjeN*, potentially *frlD*, and potentially *yahV*);
2. Energy metabolism (through *paa* gene cluster, *feaB/feaR/tynA* operon, *ynbG* due to its location next to *paa* operon, potentially *frlD*, and possibly *yddL*);
3. Cell motility (*ybiA*, *yraK* and potentially *yahV*); and
4. Stress response (*ybeQ*, *yqcG*, *yjeN* and possibly *frlD*).

Logically, these four biological processes are interconnected (Additional file 2: Fig. S12). Under osmotic stress, osmotic regulation, stress response, cell motility and metabolic changes go hand in hand. Studies have shown that *E. coli* is robust to changes in osmotic pressure [42] and this is an important adaptation strategy for enteric bacteria. While *yahV* might serve as a membrane-bound small protein that potentially stabilizes membranes during osmotic shock or modulates signal transduction during stress response; the *yddL* gene might be related to membrane permeability of solutes in response to osmotic shifts.

Published expression data [43] (for 11 out of the 33 GFs, Additional file 2: Fig. S13) related to osmotic regulation support this thought. Most of the genes/proteins have no expression at all except for the condition “NaCl Stress”. Another study [44] found the gradually increased expression of 19 related genes with growing salt concentration (Additional file 2: Fig. S14).

## Discussion

Understanding biological functions encoded in a genome involves diverse aspects including qualitative (e.g., the list of functions encoded by various genomic regions, especially of those for proteins and non-coding RNAs) as well as quantitative considerations (e.g., the sets of metabolic fluxes in various regimes of gene expression). The notion of gene function itself is a hierarchical concept involving properties of the encoded biomacromolecule (the molecular function), its role in the interaction with other cellular components (the cellular function) and its effect on the organism’s phenotype (the phenotypic function) [16].

Full genome sequencing came into being with the promise of understanding an organism in its entirety [45–47] since, in accordance with fundamental principles of molecular biology, all information for biomolecular pathways, network and mechanisms is contained in the genome sequence. The reality is that the state of the art is far from this ideal. Yet, creating a complete list of qualitative function descriptions for all genomic regions appears a feasible goal for relatively simple organisms. Once this hurdle is taken, it will set the stage for quantitative modelling with the inclusion of all relevant pathways and biomolecular mechanisms.

*E. coli* as the most studied prokaryote model organism is the prime candidate for a full gene function list [9]. As scientific literature analyses in this work show, at least some critical functional information is available for most genes (Table 1). In the case of *E. coli* strain K-12 MG1655, close to 11% of the *E. coli* genes (the so-called elite genes in FPE brackets T75, T100 and T500) have benefited from extensive literature coverage (with a cumulative FPE score of 121,771 or approximately 71% of the total relevant literature). On the other hand, only 137 out of 4273 genes have no dedicated article (see legend to Additional file 3: File 6). For 1907 genes, the available stock of scientific publications is even above the T10 level (≥ 10 FPEs), the threshold that we found as encouraging lower risk, incremental research.

Our results also devise what kind of research projects would provide the greatest intellectual impact improving the systemic understanding of *E. coli* by focusing on those genes in our lists within low FPE ranges or not mentioned in the literature at all. For example, the variation of culturing conditions or the development of new measurement methods might enable the tracing the activity of previously unobserved genes. The work of Shimada et al. [48] is a good example for such an approach. For the first time, four previously uncharacterized transcription factors were found to be single-target regulators and were associated with their gene targets by a variation of a genomic SELEX screening.

It is surprising that so many *E. coli* genes are still hypothetical with unclear functions to date despite the tremendous attention that this bacterium received in molecular life science research [12]. Most of all, the 145 K-12 MG1655 genes without dedicated articles (137 if literature for homologues in the GFs is counted, Additional file 3: File 6), especially those representing 25 softcore genome GFs including one core GF (GF_10343, b3782/*rhoL*), deserve attention and provide discovery potential. Thus, there are still *E. coli* genes with fundamental function, widely distributed among lineages but not well studied.

Also, additional investigations are needed for understanding functions of 2190 genes (~ 55% of the K-12 genome) below the T10 FPE bracket (Table 1). We expect important new discoveries in RNA biology, secondary metabolite biogenesis, intracellular transport, and functions of genome-incorporated phages/mobile elements becoming the result of this research (Fig. 3).

Yet, publication trends during the past one or two decades (Table 2, Fig. 2) are not in line with the expectation of accelerated study of uncharacterized genes (T0, T1 or T5); instead, the number of publications devoted to anyhow well-studied genes (for genes with T10 and higher) grows explosively when new gene function discovery is on decline since ~ 2009.

It appears as if the current support system of academic research tends to drive research teams away from those risky tasks:

1. We noted before that, at the transition from T5 to T10, the main fundamental research risk is overcome as, from thereon, it appears easier to produce a paper. As the typical body of publications is about 20 articles at this level, we can estimate the total cost of an *E. coli* gene function discovery at about USD 5 million (assuming 250,000$ full costs for one article). Thus, this is the order of magnitude of costs for discovering an *E. coli* gene/protein function from scratch.
2. The data presented in Table 4 convincingly shows that timelines necessary to achieve the functional insights that correspond to T10 are much larger than a decade even with the availability of modern research technologies. The important point is that the time necessary to invest into the research for understudied genes rather remembers the duration of a tenured professor’s academic appointment than the contract period of a postdoc, time-limited faculty or a typical grant. A financing mechanism that covers the > 10 year period other than just the enthusiasm of a young team leader is needed. This problem gets compounded by the absence of preliminary data for uncharacterized genes, typically a precondition to apply for a grant.

If we extrapolate into the future with the function discovery rates from the past few years assuming them unchanged from the current level, we see that it will take about two decades to empty the pool of ~ 150 completely uncharacterized K-12 MG1655 genes (the threshold T0 has been crossed annually by ~ 10 genes since 2019). With about 80 new genes getting annually into the T10 bracket, the remaining 2190 genes below T10 will probably see a status lift towards and above T10 within the next 25–30 years. Thus, this event is historically close and many of us will witness it in their life time.

If we compare the status of *E. coli* gene function discovery with that of human genes, we see that the situation is much rosier for the bacterium. Less than ~ 3.5% of all K-12 MG1655 genes have not been mentioned in any article. But in the case of the human genome, this is true for about 4000 human protein-coding genes (about 19% of the total). Then, the role of non-coding RNAs is much larger for human physiology but their functional characterization is still in its infancy [17]. If there is no change in the trends for new function discovery, a century might be a small time to soak up the pool of completely uncharacterized human protein-coding genes [17, 47].

It is also interesting to note that the onset of enhanced publication about human genes is between T25 and T30 (see Table 3 in [17]). The boundary corresponds to about 80 published articles (see Table 3B in this work). The price tag for this amount of research is in the order of USD 20–25 million. The comparison with the respective values for *E. coli* highlights how much working with human genes is more difficult, especially if information about homologues from simpler to investigate taxa is not available. Interestingly, the median number of years to achieve a literature body for human genes for a given FPE bracket is not much different from that for *E. coli* genes (compare Table 4A and B). This shows that it is easier it is to get funding for studying the more complex human systems even if this might not be scientifically justified (for example, when the gene sequence, structure and network conservation are known to be strong).

As we found in the course of this work, co-occurrence analysis of genes from the accessory genome among strains together with genomic co-localization and protein sequence analysis can lead to valuable hints. The evidence points to GF_29643 (*yahV*) and GF_4841 (*yddL*) as being involved in osmotic stress response and cell motility.

## Methods

### Datasets—*E. coli* K-12 MG1655

The DNA, protein, and coding sequence annotation files (in Gene File Format (GFF)) for the *E. coli* K-12 MG1655 strain were downloaded from the NCBI RefSeq database (assembly ID GCF_000005845.2). The gene annotations and descriptions were extracted from the GFF file following tags and keywords, respectively.

Among the 4324 coding sequence (CDS) features in the *E. coli* K-12 MG1655 strain, 36 are annotated as pseudogenes. The remaining 4288 CDS map to 4285 protein sequences (Additional file 3: File 1), out of which 4280 (for 4273 unique gene IDs) are longer than 10 amino acids (AA).

For the purpose of this work, we need a list of genes that characterizes *E. coli* as a species. Comparisons of available complete genomes show that the pool of homologous gene families (GFs) shared by all strains of *E. coli* is very small (a few hundred) [15]. At the same time, the *E. coli* pangenome is open and grows with the sophistication of ever cheaper sequencing technology and the entry of genomes especially from strains in new habitats [8, 15]. Undoubtedly, the accessory gene pool will keep increasing as more *E. coli* genomes are accumulated. On the contrary, the softcore genome (at the threshold of 92% or 95% of all genomes) is stable regardless of the addition of new genomes [15] and does provide the list of GFs that is critical for our purpose in this work.

The *E. coli* pangenome (the pangenome matrix and lists of proteins IDs in the gene families (GFs)) as well as the GF coincidence association results computed with CoinFinder [19]) were obtained from our earlier study [15] (from the respective GitHub entry [20]) in the version based on the ProteinOrtho GF clustering method [49]. That pangenome was calculated for a set of 1324 *E. coli* strains with complete genome sequences. It includes 24,889 GFs. The GF coincidence association result was evaluated based on the most common sequence types among the *E. coli* genomes. This set of strains includes 674 genomes and 6244 GFs as previously described [15].

The GFs with representation in the genome of *E. coli* K-12 MG1655 were extracted from the column GCF_000005845.2 in the pangenome matrix. This gives a total of 3973 GFs for the 4280 protein sequences (representing 4273 unique gene IDs) in the strain *E. coli* K-12 MG1655. The mapping of each of the protein IDs from the strain *E. coli* K-12 to the respective gene ID, gene name and its description are provided in Additional file 3: File 1. The lengths of the amino acid sequences are obtained from the NCBI RefSeq database.

### Datasets—six *E. coli* strains with literature mapping from the STRING database

In order to obtain the literature mapping for *E. coli* softcore genome, we first established the *E. coli* strains that can be used and, subsequently, we extracted the genes belonging to the softcore GFs. The *E. coli* strains are taken from version 11.5 of the STRING database [50]. The original literature search was for articles that mention a gene from any *E. coli* strain genome. The articles were then mapped onto reference genomes via gene names and their synonyms.

There are 11 *E. coli* strains in the STRING species list; however, only 6 of the strains have been linked to literature and RefSeq annotation. These 6 strains are *E. coli* O157H7 str. EDL933 (Taxonomy ID: 155,864), *E. coli* CFT073 (Taxonomy ID: 199,310), *E. coli* 536 (Taxonomy ID: 362,663), *E. coli* BL21 (Taxonomy ID: 469,008), *E. coli* ATCC 8739 (Taxonomy ID: 481,805) and *E. coli* K12 MG1655 (Taxonomy ID: 511,145). The corresponding genome assembly IDs for the 6 strains are provided in Additional file 1: Table S3. We also list mapping results in this table (see below for technical details). Most of the articles (98.5% of the gene-literature links) mention gene names that can be associated with gene names in strain K-12 MG1655. This does not necessarily mean that the scientific work reported in the respective article was actually done on this strain. The total amount of literature for genes with names that are not part of this strain is very small. Further, the locus_tag from each genome annotation is mapped to its corresponding protein accession ID. Subsequently, the GF is identified from the *E. coli* pangenome data from our previous study [15].

### Literature mapping—named entity recognition (NER) and text corpus construction

The NER of protein and gene names from *E. coli* in scientific texts was carried out using the text-mining software and dictionaries developed for generating version 11.5 of the STRING database [50] similarly to our previous work on the human genome [17]. Briefly, this computation is performed using a highly efficient dictionary-based NER engine implemented in C++, which is described in detail elsewhere [51]. The keyword dictionary merges synonym information from multiple sources, including the UniProtKB [52] databases. An explicit rule system [53], which combines sets of regular expressions and a list of blocked names, is applied to suppress the recognition of entity names in target texts when the respective words are frequently used to mean something else, for example in the case of certain acronyms and common English words.

To construct a literature corpus, we first downloaded all articles from the PubMed Central (PMC) Open Access Subset in BioC format [54]. Our pipeline, which was also used for the STRING database [50], performs further checks of these documents to eliminate, among other things, documents that are not written in English. The remaining articles are then merged with abstracts downloaded from the Medline/PubMed [55] to make use of abstracts whenever a full text version was not available.

Running the NER software on this literature corpus resulted in a file stating, which genes/proteins were mentioned where in which documents. The results used in this work are from a run completed on the 8th of June 2022. We found 171,590 publications represented by PubMed IDs attributable to the gene names of the strain *E. coli* K-12 MG1655. 174,120 literature articles (about six *E. coli* strains) mention at least one gene from 3056 GFs belonging to the 95%-threshold *E. coli* softcore genome [15, 20].

It is important to note that the result of this process can never be perfect [17]. Firstly, if a name is missing in the dictionary, the corresponding mentions of the gene or protein will be missed. Secondly, although we block problematic names, the names in the dictionary will sometimes give false positives where the name does not refer to the gene/protein in question. For example, gene names and strain names could be identical but this conflict cannot be resolved within this approach. Exclusion rules are in place to suppress such false-positive NER but this makes the automated literature assignment procedure rather underestimating the publication coverage of a gene. As we see in the analyses done in this work, this effect is quite widely spread. Thirdly, if we do not have access to the full-text version of a document, we will obviously only be able to find mentions in the title and abstract. Fourthly, there is no protection against typos in the original text that can create the appearance/absence of gene/RNA/protein names.

Lastly, we need to emphasize that we wish to find articles that are dedicated to specific genes and report substantial information about the function of a gene. Thus, pure high-throughput studies, especially full genome sequencing papers, which do not mention the gene name in some functional context in the main text (but, maybe, in some tables in the Additional file 1), are not helpful in this context and are not counted by our approach.

For these reasons, it would be problematic to attempt to judge, for example, when the first paper for a specific gene was published, since the first mention can easily have been missed, or the first identified mention could be a false positive. However, the quality of the results is easily good enough to make statistical observations [17, 56], for example, about how much is published about one gene compared to another, or how the publication count for a given gene changes over time. Further, as a trend, a genome region mentioned in a larger set of articles will be functionally better understood than another one with much less literature or even with no paper hit at all.

We have also run the NER software on the same literature corpus for human gene name matching and used the results from a computation completed on the 25th of October 2022 in this work for comparison with *E. coli* data (for Tables 3B and 4B).

### Literature mapping—fractional counting of entity names and determination of full publication equivalents (FPE)

We follow previously described procedures [17]. In brief, a document can mention multiple proteins without pertaining equally much to all of them. To address this, we use a fractional counting scheme [56] in which each paper that mentions at least one gene/protein contributes a total count of 1, which is distributed across the mentioned gene/proteins relative to how many times each of them was mentioned. Thus, the total fractional count *f*_*i*_ for protein or gene *i* is

$$f_{i} = \mathop \sum \limits_{j \in D} \frac{{n_{ij} }}{{n_{j} }}.$$

Here, *D* is the document set, ${n}_{ij}$ is the number of times protein or gene *i* is mentioned in document *j*, ${n}_{\bullet j}$ is total number of mentions of any gene/protein in document *j*.

We generated a master file where each line contains a genomic entity name, a publication identifier, the publication date and the fractional count associated with that genomic entity name. From this source, it possible to assess the amount of literature published about a given genomic entity (the literature score) in periods of time by summing up the respective fractional counts for publications in the years considered. We define a literature score of one as full publication equivalent (FPE) [17], the amount of literature necessary to achieve one idealized publication solely dedicated to a single genomic entity (gene, protein or non-coding RNA). As was shown before [56], more publications per named genomic entity strongly correlate with more complete insight into its functional aspects. Thus, further in the text, we will use the number of FPEs per named genomic entity as proxy for the level of knowledge about its biological function.

For the convenience of the reader, the FPE score for each gene in each publication document will be provided in Additional files to ease reproduction of results. The software “R” and Microsoft Excel were used for statistical tests.

### Mapping gene IDs to the COG reference database

The COG function codes for *E. coli* K-12 MG1655 genes are extracted from the NCBI COG database [57–59]. In total, there are 3542 gene IDs that can be mapped to a total of 2150 COG IDs relevant to the *E. coli* K-12 MG1655 strain in the database. The functional code for each COG ID is determined from the file “cog-20.def.tab” as downloaded from NCBI COG database. When a COG entry has multiple functional codes, the first functional code for this COG ID was used.

To investigate the COG functional distributions of the genes in each FPE score range, we mapped the genes in each FPE score range to their COG IDs from NCBI COG reference database and identified how many of them remain unmapped. If a gene is mapped to multiple COG IDs, the weightage of the COG ID will be assigned as a fraction of the totally mapped COG IDs, accordingly. For example, if one gene is mapped to two COG IDs, then, the weightage for each COG ID of the gene will be 0.5, respectively. Finally, the functional codes for each mapped COG ID are identified and assigned the weightage as defined previously. There are 26 functional codes altogether, but only 23 functional codes are relevant to *E. coli* K-12 MG1655 (letters A and C through X). There is no gene mapped to the other 3 functional codes (i.e., B, Y and Z). Any of the unmapped genes to COG ID will be assigned “unmapped”. The assignments for the functional codes can be found in Additional file 1: Table S7.

### Integrating *E. coli *pangenome data and coincidentally associated gene families for computational investigation of uncharacterized genes

For the GF coincidence associative analysis, we used the previously calculated coincident association results obtained with CoinFinder [19] for the most common sequence types in *E. coli*, a set consisting of 674 genomes. The coincident association results were based on the accessory GFs that we found present in at least 10 and at most 640 *E. coli* genomes. The significant association threshold for a pair of GFs was set at a *P*-value = 10^–20^.

We extracted the genes without any mapped literature (FPE score zero) and evaluated if any of these genes have associated GFs based on the CoinFinder analysis. The genes with significantly associated GFs were further evaluated from two perspectives, i.e. (1) how well the associated GFs are functionally studied in accordance to available literature, and (2) do the associated GFs form an operon (or a synteny cluster)? Computational determination of synteny followed the procedure applied earlier [15]. The combined information was then used to infer the biological pathway or process potentially involving the uncharacterized genes.

## Supplementary Information

**Additional file 1: Table S1.** Manual checking of 176 genes from K-12 MG1655 and of 39 GFs from the *E. coli* softcore genome without any automatically assigned literature. **Table S2.** List of 43 genes with aggregated FPE score ≥ 500. The genes are sorted according to its aggregated FPE Score. **Table S3.** The total number of genes and publications together with the mapped softcore and publications for the six *E. coli* strains. **Table S4.** The number of *E. coli* softcore genes as well as sum of literature score in various FPE score ranges. **Table S5.** The growing trend of literature coverage for *E. coli* softcore genes in various FPE score thresholds. **Table S6.** Exclusion lists of genes from the group of 176 K-12 MG1655 genes.

**Additional file 2: Fig. S1.** We show the total number of *E. coli* softcore genes’ related publications (red line relative to the left y-axis) and the total number of genes mentioned in the respective literature (blue line relative to the right y-axis) from year 1939 up to year 2021. The blue dashed vertical lines mark the expansion period for the total number of genes from year 1965 to 2009. It apparently plateaus after the year 2019. The red dashed vertical lines at years 1970 and 2007 indicate two periods of publication dynamics: 1970–2007 and 2007–2021. The ratio of the number of publications in each year to the total number of new genes identified in each year is shown in the insert. **Fig. S2.** FPE plots for different FPE score ranges from year 1960 until 2021 for *E. coli* K-12 genes are separately shown for five different categories, i.e. (**A**) very understudied, (**B**) understudied, (**C**) moderately studied, (**D**) intensively studied and (**E**) very intensively studied. The y-axis is given in the same scale for visual comparison across different categories. **Fig. S3.** We illustrate the number of new genes of *E. coli* K-12 achieving the FPE score ranges (T0, T1, T5, T10, T15, T20, T25, T30, T35, T40, T45, T50, T75, T100, T500) across the years in (**A**) phase 1 and (**B**) phase 2 periods. The linear regression line (number of new genes (y-axis) versus year (x-axis)) is shown. The magnitude of the slope is provided in Table 2. **Fig. S4.** FPE plots for different FPE score range from year 1960 until 2021 for *E. coli* softcore genes are separately shown for five different categories, i.e. (**A**) very understudied, (**B**) understudied, (**C**) moderately studied, (**D**) intensively studied and (**E**) very intensively studied. The y-axis is given in the same scale for visual comparison across different categories. **Fig. S5.** We illustrate the number of new genes of the *E. coli* softcore genome achieving the FPE score ranges (T0, T1, T5, T10, T15, T20, T25, T30, T35, T40, T45, T50, T75, T100, T500) across the years in (**A**) phase 1 and (**B**) phase 2 periods. The linear regression line (number of new genes (y-axis) versus year (x-axis)) is shown. The magnitude of the slope is provided in Additional file 1: Table S5. **Fig. S6.** Prediction of the transmembrane (TM) region in the protein sequence *yahV* (GF_29643) in *E. coli* K-12 MG1655 using TMHMM 2.0. The TM region is predicted to cover positions 4-23 of the protein sequence. **Fig. S7.** The upstream and downstream genes of *yahV* based on NCBI RefSeq. The *betABIT* operon is upstream of *yahV* gene. *betABIT* is expressed only under aerobic condition during osmotic stress for production of osmoprotectants. The *pdeL* gene, on the other hand, is downstream of the gene *yahV*. The *pdeL* gene appears involved in the regulation of cell motility. **Fig. S8.** Neighboring gene families of GF_29643 (*yahV*; circled in red) focusing on genomes that carry GF_29643. Ten GFs upstream and ten GFs downstream of GF_29643 are extracted and investigated. Each GF is represented as a node and two nodes are linked by an edge if they are next to each other. The thickness of the edge represents the weighted link between the two GFs. Clearly, GF_29643’s genomic position is conserved across the *E. coli* genomes that carry the *yahV* gene. Note that GF_8617 represents the betT gene and GF_25808 contains the *pdeL* gene. **Fig. S9.** The predicted transmembrane beta-barrel (TMBB) structure of protein *yddL* (GF_4841) using BetAware-Deep. The predicted localization is outer membrane TMBB with the overall TMBB probability of 0.93. There are four (4) TM β-strand segments as shown in the figure. **Fig. S10.** We illustrate the GFs associated with GF_29643, GF_4841 and GF_8394. The associated GFs of these three GFs have high overlap with each other and, therefore, can be related. Each node represents a GF and the edge (connecting line) indicates a significant coincident association between nodes (*P*-value ≤ 1 × 10–20). The size of the node is determined by the node’s degree (the number of associated GFs). The color of the node is represented by a gradient color from grey to red which is determined by the node’s degree as well. The three cluster-founding GFs are highlighted by red arrows. Please note that only 60 out of 68 GFs found are present in *E. coli* K-12 MG1655. **Fig. S11.** The number of overlapping associated GFs among three GFs, i.e., GF_29643, GF_8394 and GF_4841. **Fig. S12.** Manual annotation of associated GFs to GF_29643 (*yahV*), GF_4841 (*yddL*), and GF_8394 (*paaE*). There are four potential biological processes related to these 3 GFs, i.e. osmotic regulation, stress response, cell motility and energy metabolism. The corresponding genes are given for each biological process. The genes with unclear function are given as “Not Clear”. **Fig. S13.** The protein expression of 11 genes extracted from Caglar’s proteomics data. Only 11 genes out of 30 gene families, which are fully connected or significantly associated to each other, have the protein expression in Caglar’s proteomics data. Please note that the *E. coli* strain used in Caglar’s study is *E. coli* REL606, which belongs to phylogroup A (sequence type ST93). This is different from *E. coli* K-12 MG1655, which has sequence type ST10. The highlighted box (with a red dashed line) emphasizes the expression results from cultures under NaCl_Stress condition. **Fig. S14.** We visualize the gene expression of 19 genes extracted from the Metris et al. data in accordance with osmotic conditions. These 19 genes are from our set of 30 GFs, which are fully connected or significantly associated to each other. Please note that the *E. coli* strain used in Metris’ study is *E. coli* K12 MG1655, which is the same as the *E. coli* strain in our analysis.

**Additional file 3: File 1.** Details about *E. coli* K-12 MG1655 genes with mapped literature FPE-scores. This file provides the list of *E. coli* K-12 MG1655 protein accession IDs (ProteinID) with the gene IDs (GeneID), gene names (GeneName), protein lengths (Length), product descriptions (Product), GF IDs from the *E. coli* pangenome study (GF_ID) and the aggregated FPE score till June 2022 for the gene ID. **File 1B.** Details about *E. coli* softcore genome GFs with mapped literature FPE-scores. This file provides the list of *E. coli* softcore genome GFs with gene family ID from the *E. coli* pangenome study (GF_ID), the representative sequence, the product description (Product), gene name (GeneName) and the aggregated FPE-score till June 2022. **File 2.** The mapping of *E. coli* K-12 MG1655 gene id (Gene) to the PubmedID, the year of publication for the respective PubmedID (Year), the number of times the gene appear in that PubmedID (Count) and the calculated FPE score (FPE_Score) for the gene id. The sum of FPE_Score for each unique PMID should be equal to 1. **File 2B.** The mapping of *E. coli* softcore gene family ID (GF_ID) to the PubmedID, the year of publication for the respective PubmedID (Year), the number of times the gene appear in that PubmedID (Count) and the calculated FPE score (FPE_Score) for the GF_ID. The sum of FPE_Score for each unique PMID should be equal to 1. **File 3.** This supplementary file provides the total number of *E. coli* K-12 MG1655 gene id that has been mentioned (#genes have been mentioned) till the specified year (Year). **File 3B.** This supplementary file provides the total number of *E. coli* softcore gene family id (GF_ID) that has been mentioned (#GF_ID have been mentioned) till the specified year (Year). **File 4.** This supplementary file provides the year when the *E. coli* K-12 MG1655 gene id (GeneID) was first mentioned in the literature. This is an approximation based on the literature mapping data. **File 4B.** This supplementary file provides the year when the *E. coli* softcore genome GF_ID (GF_ID) was first mentioned in the literature. This is an approximation based on the literature mapping data. **File 5.** The data table for *E. coli* K-12 MG1655 with year of study on rows and Tx on columns. The value for each row represents the number of genes achieved Tx (T0, T1, …, T500) in the respective year. **File 5B.** The data table for *E. coli* softcore genome with year of study on rows and Tx on columns. The value for each row represents the number of genes achieved Tx (T0, T1, …, T500) in the respective year. **File 6.** Manual analysis of the lists of 176 genes (*E. coli* K-12 MG1655) and 39 GFs (95%-threshold softcore genome) without automatically assigned publications. This file (first worksheet) shows the list of 176 genes (177 transcripts due to *yibX* with two transcripts YP_010051208.1 (80AA) and YP_010051209.1 (24AA)) that do not have any literature mapped using our automated procedure. These genes/transcripts are reinvestigated by two approaches, i.e. (1) mapping onto the GFs from the previously published pangenome study and (2) manual queries on PubMed. If the gene/transcript does not have any mapped literature directly or via another gene from the respective pangenome GF after reinvestigation, it was categorized as “Unmapped”. The list is ordered according to the gene family ID (GF_ID) with its pangenome category (accessory, softcore or core), gene id (GeneID) and gene name (GeneName) information given in the table. The columns “Homologue”, “Manual Check” and “Unmapped” contain the binary entry of 0 or 1 as the indicator of the reinvestigation. An entry of 1 “Manual Check” column suggests that publications relevant for the gene/transcript/protein’s function can be recovered through manual checking of PUBMED. An entry of 1 in the “Homologue” column shows that one or more of the GF member genes have mapped literature from the K-12 literature (11 cases) or from the manual PUBMED searches. The value 0 indicates otherwise. Genes that cannot be mapped either through homologous mapping or manual checking will be assigned 1 in the “Unmapped” column; otherwise, it is assigned as 0. The “Pangenome_Category” classifies the GF_ID into “accessory”, “softcore” and “core” genome according to our previously published *E. coli* pangenome study. The softcore genome is defined as set of GF IDs that are present in at least 95% of the *E. coli* strains. The core genome is defined as set of GF IDs that are present in all *E. coli* strains of the *E. coli* pangenome study (using 1324 completely sequenced genomes). Any other GF ID that is found in less than 95% of the *E. coli* strains is categorized as accessory genome. As a summary, 137 proteins encoded by the 177 transcripts from 176 genes remain unmapped. The remaining 40 cases are explained as: 31 can be assigned publications via a manual PUBMED search (Additional file1: Table S1). 12 have literature-annotated homologues in their GF (11 by automated mapping of the K-12 literature, one (*ibsE*) after including manual PUBMED search results). In three cases, both conditions apply. Thus, the 176 genes (177 transcripts/proteins) map onto 171 GFs. The 137 unmapped proteins belong to 135 GFs, out of which 25 are part of the softcore genome (one is even from the core genome) and 110 are accessory genome GFs. Similarly, we analyzed the 39 GFs of the softcore genome (second worksheet: 39 GFs-softcore analysis, see also legend for Additional file 1: Table S1) that were not automatically mapped to literature. Ten of them contain K-12 genes that were annotated with articles by our manual PUBMED searches. 23 GFs coincide with GFs from the K-12 gene mapping that have no associated publication even after manual testing. Among six GFs with no K-12 gene, 3 can be mapped to multiple publications, whereas the other 3 remain unmapped. Thus, 26 GFs from the softcore genome have no assigned publication. **File 7.** The list of 45 gene family IDs (GF_ID) that have at least one significant coincidently associated GF_ID based on the CoinFinder analysis. The number of significantly associated GF_ID (Num_AGs) is provided together with the information on how many of these significantly associated GF_ID have FPE score > 0 (Num_AGs_with_Literature) and its percentage (Num_AGs_with_Literature/Num_AGs * 100%). The total FPE score of these associated GF_IDs are given (Total_FPE). The associated gene name (GeneName) and product description (Product) are listed as well. The highlighted rows (in same color) are the clusters of GF_IDs, which share common associated GF_IDs. **File 8.** The annotation for 30 gene family IDs (GF_ID) that are significantly associated to GF_29643, GF_4841 and GF_8394 (highlighted rows). The gene id (GeneID), gene name (GeneName), product description (Product), mapped NCBI COG reference ID (COG ID), COG Functional code, COG Functional Description and the inferred potential biological process are given. Finally, further relevant information is provided in the remarks column. The rows with red text are added because they are either softcore genes (*paaJ* and *paaY*) or borderline significantly associated (*tynA*) in the coincidence analysis. Therefore, there are 33 GFs in total in this spreadsheet.

## Data Availability

All data generated and analyzed during this study are included in this published article and its Additional files. Additional file 1 provides 6 Tables. Additional file 2 (a zip-package) contains 14 Figures. The zip-package Additional file 3 provides a legends file with content description of 13 further files contained in the package.

## Abbreviations

CDS: Coding sequence
DNA: Deoxyribonucleic acid
FPE: Full publication equivalent
*E. coli*: *Escherichia coli*
GF: Gene/protein family (family of sequentially similar genes/proteins thought to be orthologous)
ID: Identifier
NCBI: National Center of Biological Information USA
USD: United States Dollar
