# Supplementary material for: About the dark corners in the gene function space of Escherichia coli remaining without illumination by scientific literature
Source: Biol Direct. 2023 Feb 28;18:7. doi: 10.1186/s13062-023-00362-0 (PMC9976479; doi:10.1186/s13062-023-00362-0)
Supplement: Supplementary file 1 — Additional file 1: Table S1. Manual checking of 176 genes from K-12 MG1655 and of 39 GFs from the E. coli softcore genome without any automatically assigned literature. Table S2. List of 43 genes with aggregated FPE score ≥ 500. The genes are sorted according to its aggregated FPE Score. Table S3. The total number of genes and publications together with the mapped softcore and publications for the six E. coli strains. Table S4. The number of E. coli softcore genes as well as sum of literature score in various FPE score ranges. Table S5. The growing trend of literature coverage for E. coli softcore genes in various FPE score thresholds. Table S6. Exclusion lists of genes from the group of 176 K-12 MG1655 genes. [file 13062_2023_362_MOESM1_ESM.pdf]

**Supplementary Table S1**
**Manual checking of 176 genes from K-12 MG1655 and of 39 GFs from the *E. coli* softcore genome without any automatically assigned literature**

For the list of 176 genes from the genome of E. coli K-12 MG1655, a manual PUBMED search was carried out using the following syntax. The command is provided for the example of gene b0276/yagJ and it was modified for the other genes correspondingly:

https://pubmed.ncbi.nlm.nih.gov/?term=((b0276[Text Word]) OR (yagJ[Text Word])) AND (Escherichia coli[Text Word])

The search result was empty for 145 genes and delivered a non-zero output for 31 genes enumerated in this table (we manually excluded clearly false-positive hits if any were generated). In all articles listed, the respective gene is mentioned in the main text or even in the abstract. To note, thirteen of these studies seem large-scale, typically genome-wide analyses and hardly count as dedicated articles in the understanding of this work.

Yet, the automated literature assignment missed all the articles listed in the table contrary to our intentions. The explanation is due to context exclusion rules in the rule set introduced to suppress other false-positive literature assignments that might be caused by usage of gene names in a non-gene name circumstance. Thus, the automated procedure has a trend for under-estimating FPE scores.

24 genes have one article missed. For five genes, it is a pair of articles. For the two genes b1265 (*trpL*) and b3643 (*rph*), there are a larger number of mapped articles, and we list only a few of the relevant ones.

Some genes have ambiguous naming and identification of mapped literatures requires some extra efforts. For example, the name "*trpL*" is a *trp* operon leader peptide and is used in experimental models to study transcriptional pausing mechanisms[1]. But in the literature search, we can find TrpL coding for a dietary-related experiment. As another example, searching for the gene *ibsC* in *E. coli* returns some articles related to irritable bowel syndrome (IBS)[2], which are clearly not about the gene *ibsC*.

This manual PUBMED check does not exclude certain ambiguities due to other limitations inherent to our approach. Sometimes, gene names are mentioned in a very convoluted form. For example, in the case of gene No. 7 in the list (b1392/*paaE*), the automated literature mapping procedure did not assign any paper and the manual check found just one. Yet as we discovered later, Teufel *et al.*[3] published a small report mentioning "*paaABCDE*", an expression denoting a group of genes in the operon *paa* including the gene *paaE*, among other genes.

Similarly, we analysed the 39 GFs of the softcore genome that where not automatically mapped to literature (see also Supplementary File 6/second worksheet). 33 of them contain K-12 genes.

A subgroup of ten GFs were found being annotated with articles by our manual PUBMED searches (GF_1516, GF_4189, GF_9029, GF_9184, GF_10279, GF_10300, GF_10333, GF_11896, GF_16540, and GF_17432). The remaining 23 of them (GF_1804, GF_2294, GF_2576, GF_2906, GF_3516, GF_3865, GF_4055, GF_7378, GF_8799, GF_9471, GF_9529, GF_10297, GF_10316, GF_10343 (from the core, with b3782/*rhoL*), GF_14365, GF_15465,

GF_17477, GF_18519, GF_22441, GF_24357, GF_26787, GF_27674, GF_28417) coincide with GFs from the K-12 gene mapping that have no associated publication even after manual testing.

A similar check for six GFs that do not contain K-12 genes suggests that 3 of them (GF_2374/*lafU*[4, 5], GF_3137/*fruR*[6-9] and GF_4428/*crl*[10-13]) can be mapped to publications. The remaining 3 (GF_382, GF_2374, GF_3137, GF_4254, and GF_19709) are described as hypothetical/unknown function and remain unmapped. Thus, we have 26 softcore genome GFs without dedicated articles including one core genome GF.

| No. | Gene | ProteinID | GeneName | Pubmed ID | Large-scale study |
|---|---|---|---|---|---|
| 1 | b0012 | YP_009518733.1 | *mbiA/htgA* | 24111745[14] | - |
| 2 | b0276 | YP_009518744.1 | *yagJ* | 27718375[15] | 27718375[15] |
| 3 | b1149 | YP_009518764.1 | *ymfN* | 14733619[16] | 14733619[16] |
| 4 | b1151 | YP_009518765.1 | *beeE* | 14733619[16] | 14733619[16] |
| 5 | b1265 | NP_415781.1 | *trpL* | 35833713[1], 3609747[17], 9457797[18], 2045362[19], … | - |
| 6 | b1366 | YP_009518777.1 | *ydaY* | 14592990[20] | 14592990[20] |
| 7 | b1392 | NP_415910.1 | *paaE* | 31689071[3], 17259607[21] | - |
| 8 | b1543 | YP_009518786.1 | *ydfJ* | 21744086[22], 17222132[23] | 17222132[23] |
| 9 | b1548 | YP_009518788.1 | *nohA* | 11101675[24] | - |
| 10 | b1567 | YP_009518792.1 | *ydfW* | 24025676[25] | - |
| 11 | b1715 | NP_416230.1 | *pheM* | 28351917[26] | - |
| 12 | b2356 | YP_009518803.1 | *yfdM* | 30995473[27] | - |
| 13 | b2358 | YP_009518804.1 | *yfdO* | 21266997[28] | - |
| 14 | b2598 | NP_417089.1 | *pheL* | 21177642[29] | - |
| 15 | b3418 | NP_417877.1 | *malT* | 23934774[30] | - |
| 16 | b3586 | NP_418043.1 | *yiaV* | 15668009[31] | 15668009[31] |
| 17 | b3643 | YP_009518822.1 | *rph* | 8501045[32], 1512252[33], 28808133[34], 1644789[35], ... | - |
| 18 | b4419 | YP_025297.1 | *ldrA* | 24513967[36] | - |
| 19 | b4545 | YP_009518805.1 | *ypdJ* | 24025676[25] | - |
| 20 | b4606 | YP_001165325.1 | *ypfM* | 29808326[37] | 29808326[37] |
| 21 | b4664 | YP_002791256.1 | *ibsD* | 32029755[38] | 32029755[38] |
| 22 | b4665 | YP_002791255.1 | *ibsC* | 32516493[39], 20980267[40] | - |
| 23 | b4667 | YP_002791247.1 | *ibsA* | 20453032[41] | - |
| 24 | b4668 | YP_002791248.1 | *ibsB* | 32268068[42] | 32268068[42] |
| 25 | b4702 | YP_003933616.1 | *mgtL* | 29100053[43], 28644990[44] | 28644990[44] |
| 26 | b4723 | YP_009518761.1 | *ymcF* | 28861998[45] | 28861998[45] |
| 27 | b4724 | YP_009518790.1 | *ynfQ* | 28861998[45] | 28861998[45] |
| 28 | b4725 | YP_009518806.1 | *rseD* | 28924029[46] | - |
| 29 | b4727 | YP_009518737.1 | *yacM* | 15044829[47], 29645342[48] | 29645342[48] |
| 30 | b4766 | YP_010051174.1 | *argL* | 319913[49], 4553006[50] | - |
| 31 | b4803 | YP_010051176.1 | *speFL* | 32094585[51] | - |

1. Jeanneau,S., Jacques,P.E., & Lafontaine,D.A. Investigating the role of RNA structures in transcriptional pausing using in vitro assays and in silico analyses. *RNA. Biol.* **19**, 916-927 (2022).
2. Takakura,W. & Pimentel,M. Small Intestinal Bacterial Overgrowth and Irritable Bowel Syndrome - An Update. *Front Psychiatry* **11**, 664 (2020).
3. Teufel,R. *et al.* Bacterial phenylalanine and phenylacetate catabolic pathway revealed. *Proc. Natl. Acad. Sci. U. S. A* **107**, 14390-14395 (2010).
4. Ren,C.P., Beatson,S.A., Parkhill,J., & Pallen,M.J. The Flag-2 locus, an ancestral gene cluster, is potentially associated with a novel flagellar system from Escherichia coli. *J. Bacteriol.* **187**, 1430-1440 (2005).
5. Hsiao,F.S., Yang,S.K., Lin,J.M., Chen,Y.W., & Chen,C.S. Protein interactome analysis of iduronic acid-containing glycosaminoglycans reveals a novel flagellar invasion factor MbhA. *J. Proteomics.* **208**, 103485 (2019).
6. Ramseier,T.M. *et al.* In vitro binding of the pleiotropic transcriptional regulatory protein, FruR, to the fru, pps, ace, pts and icd operons of Escherichia coli and Salmonella typhimurium. *J. Mol. Biol.* **234**, 28-44 (1993).
7. Negre,D. *et al.* FruR-mediated transcriptional activation at the ppsA promoter of Escherichia coli. *J. Mol. Biol.* **276**, 355-365 (1998).
8. Ow,D.S. *et al.* Inactivating FruR global regulator in plasmid-bearing Escherichia coli alters metabolic gene expression and improves growth rate. *J. Biotechnol.* **131**, 261-269 (2007).
9. Liu,L., Duan,X., & Wu,J. Modulating the direction of carbon flow in Escherichia coli to improve l-tryptophan production by inactivating the global regulator FruR. *J. Biotechnol.* **231**, 141-148 (2016).
10. Lelong,C., Rolland,M., Louwagie,M., Garin,J., & Geiselmann,J. Mutual regulation of Crl and Fur in Escherichia coli W3110. *Mol. Cell Proteomics.* **6**, 660-668 (2007).
11. Dudin,O., Lacour,S., & Geiselmann,J. Expression dynamics of RpoS/Crl-dependent genes in Escherichia coli. *Res. Microbiol.* **164**, 838-847 (2013).
12. Zhao,S. *et al.* Resonance assignments of sigma factor S binding protein Crl from Escherichia coli. *Biomol. NMR Assign.* **13**, 223-226 (2019).
13. Naziri,Z., Kilegolan,J.A., Moezzi,M.S., & Derakhshandeh,A. Biofilm formation by uropathogenic Escherichia coli: a complicating factor for treatment and recurrence of urinary tract infections. *J. Hosp. Infect.* **117**, 9-16 (2021).
14. Fellner,L. *et al.* Phenotype of htgA (mbiA), a recently evolved orphan gene of Escherichia coli and Shigella, completely overlapping in antisense to yaaW. *FEMS Microbiol. Lett.* **350**, 57-64 (2014).
15. Sargentini,N.J., Gularte,N.P., & Hudman,D.A. Screen for genes involved in radiation survival of Escherichia coli and construction of a reference database. *Mutat. Res.* **793-794**, 1-14 (2016).
16. Mehta,P., Casjens,S., & Krishnaswamy,S. Analysis of the lambdoid prophage element e14 in the E. coli K-12 genome. *BMC. Microbiol.* **4**, 4 (2004).
17. Sano,K. & Matsui,K. Structure and function of the trp operon control regions of Brevibacterium lactofermentum, a glutamic-acid-producing bacterium. *Gene* **53**, 191-200 (1987).

18. Mori,H., Iida,A., Fujio,T., & Teshiba,S. A novel process of inosine 5'-monophosphate production using overexpressed guanosine/inosine kinase. *Appl. Microbiol. Biotechnol.* **48**, 693-698 (1997).
19. Bae,Y.M. & Stauffer,G.V. Genetic analysis of the attenuator of the Rhizobium meliloti trpE(G) gene. *J. Bacteriol.* **173**, 3382-3388 (1991).
20. Gurvich,O.L. *et al.* Sequences that direct significant levels of frameshifting are frequent in coding regions of Escherichia coli. *EMBO J.* **22**, 5941-5950 (2003).
21. Nogales,J. *et al.* Characterization of the last step of the aerobic phenylacetic acid degradation pathway. *Microbiology (Reading. )* **153**, 357-365 (2007).
22. Tang,G. *et al.* Identification of a novel bacterial K(+) channel. *J. Membr. Biol.* **242**, 153-164 (2011).
23. Domka,J., Lee,J., Bansal,T., & Wood,T.K. Temporal gene-expression in Escherichia coli K-12 biofilms. *Environ. Microbiol.* **9**, 332-346 (2007).
24. Vassinova,N. & Kozyrev,D. A method for direct cloning of fur-regulated genes: identification of seven new fur-regulated loci in Escherichia coli. *Microbiology (Reading. )* **146 Pt 12**, 3171-3182 (2000).
25. Mohd Yusoff,M.Z., Hashiguchi,Y., Maeda,T., & Wood,T.K. Four products from Escherichia coli pseudogenes increase hydrogen production. *Biochem. Biophys. Res. Commun.* **439**, 576-579 (2013).
26. Gordon,G.C., Cameron,J.C., & Pfleger,B.F. RNA Sequencing Identifies New RNase III Cleavage Sites in Escherichia coli and Reveals Increased Regulation of mRNA. *mBio.* **8**, (2017).
27. Song,S., Guo,Y., Kim,J.S., Wang,X., & Wood,T.K. Phages Mediate Bacterial Self-Recognition. *Cell Rep.* **27**, 737-749 (2019).
28. Wang,X. *et al.* Cryptic prophages help bacteria cope with adverse environments. *Nat. Commun.* **1**, 147 (2010).
29. Gurvich,O.L., Nasvall,S.J., Baranov,P.V., Bjork,G.R., & Atkins,J.F. Two groups of phenylalanine biosynthetic operon leader peptides genes: a high level of apparently incidental frameshifting in decoding Escherichia coli pheL. *Nucleic Acids Res.* **39**, 3079-3092 (2011).
30. Schiefner,A., Gerber,K., Brosig,A., & Boos,W. Structural and mutational analyses of Aes, an inhibitor of MalT in Escherichia coli. *Proteins* **82**, 268-277 (2014).
31. Hu,Y. & Coates,A.R. Transposon mutagenesis identifies genes which control antimicrobial drug tolerance in stationary-phase Escherichia coli. *FEMS Microbiol. Lett.* **243**, 117-124 (2005).
32. Jensen,K.F. The Escherichia coli K-12 "wild types" W3110 and MG1655 have an rph frameshift mutation that leads to pyrimidine starvation due to low pyrE expression levels. *J. Bacteriol.* **175**, 3401-3407 (1993).
33. Jensen,K.F., Andersen,J.T., & Poulsen,P. Overexpression and rapid purification of the orfE/rph gene product, RNase PH of Escherichia coli. *J. Biol. Chem.* **267**, 17147-17152 (1992).
34. Bowden,K.E., Wiese,N.S., Perwez,T., Mohanty,B.K., & Kushner,S.R. The rph-1-Encoded Truncated RNase PH Protein Inhibits RNase P Maturation of Pre-tRNAs with Short Leader Sequences in the Absence of RppH. *J. Bacteriol.* **199**, (2017).
35. Kelly,K.O., Reuven,N.B., Li,Z., & Deutscher,M.P. RNase PH is essential for tRNA processing and viability in RNase-deficient Escherichia coli cells. *J. Biol. Chem.* **267**, 16015-16018 (1992).

36. Yamaguchi,Y., Tokunaga,N., Inouye,M., & Phadtare,S. Characterization of LdrA (long direct repeat A) protein of Escherichia coli. *J. Mol. Microbiol. Biotechnol.* **24**, 91-97 (2014).
37. Ng,T.W. *et al.* Differential gene expression in Escherichia coli during aerosolization from liquid suspension. *Appl. Microbiol. Biotechnol.* **102**, 6257-6267 (2018).
38. Ye,C., Lin,H., Zhang,M., Chen,S., & Yu,X. Characterization and potential mechanisms of highly antibiotic tolerant VBNC Escherichia coli induced by low level chlorination. *Sci. Rep.* **10**, 1957 (2020).
39. Mok,W.W., Patel,N.H., & Li,Y. Decoding toxicity: deducing the sequence requirements of IbsC, a type I toxin in Escherichia coli. *J. Biol. Chem.* **285**, 41627-41636 (2010).
40. Jahanshahi,S. & Li,Y. An Effective Method for Quantifying RNA Expression of IbsC-SibC, a Type I Toxin-Antitoxin System in Escherichia coli. *Chembiochem.* **21**, 3120-3130 (2020).
41. Han,K., Kim,K.S., Bak,G., Park,H., & Lee,Y. Recognition and discrimination of target mRNAs by Sib RNAs, a cis-encoded sRNA family. *Nucleic Acids Res.* **38**, 5851-5866 (2010).
42. Li,S. *et al.* Genome-Wide CRISPRi-Based Identification of Targets for Decoupling Growth from Production. *ACS Synth. Biol.* **9**, 1030-1040 (2020).
43. Chadani,Y. *et al.* Intrinsic Ribosome Destabilization Underlies Translation and Provides an Organism with a Strategy of Environmental Sensing. *Mol. Cell* **68**, 528-539 (2017).
44. Chueca,B., Perez-Saez,E., Pagan,R., & Garcia-Gonzalo,D. Global transcriptional response of Escherichia coli MG1655 cells exposed to the oxygenated monoterpenes citral and carvacrol. *Int. J. Food Microbiol.* **257**, 49-57 (2017).
45. D'Lima,N.G. *et al.* Comparative Proteomics Enables Identification of Nonannotated Cold Shock Proteins in E. coli. *J. Proteome. Res.* **16**, 3722-3731 (2017).
46. Yakhnin,H., Aichele,R., Ades,S.E., Romeo,T., & Babitzke,P. Circuitry Linking the Global Csr- and sigma(E)-Dependent Cell Envelope Stress Response Systems. *J. Bacteriol.* **199**, (2017).
47. Zalacain,M. *et al.* A global approach to identify novel broad-spectrum antibacterial targets among proteins of unknown function. *J. Mol. Microbiol. Biotechnol.* **6**, 109-126 (2003).
48. VanOrsdel,C.E. *et al.* Identifying New Small Proteins in Escherichia coli. *Proteomics.* **18**, e1700064 (2018).
49. Sens,D., Natter,W., & James,E. Evolutionary drift of the argF and argl genes. Coding for isoenzyme forms of ornithine transcarbamylase in E. coli K12. *Cell* **10**, 275-285 (1977).
50. Syvanen,J.M. & Roth,J.R. Structural genes for ornithine transcarbamylase in Salmonella typhimurium and Escherichia coli K-12. *J. Bacteriol.* **110**, 66-70 (1972).
51. Herrero,D., V *et al.* Ornithine capture by a translating ribosome controls bacterial polyamine synthesis. *Nat. Microbiol.* **5**, 554-561 (2020).

**Supplementary Table S2**

**List of 43 genes with aggregated FPE score ≥ 500. The genes are sorted according to its aggregated FPE Score.**

| GeneID | GeneName | Product Description | GF_ID | FPE Score |
|---|---|---|---|---|
| **b0344** | *lacZ* | beta-galactosidase | GF_16470 | 7771.026 |
| **b0014** | *dnaK* | chaperone protein DnaK | GF_16808 | 4863.671 |
| **b1923** | *fliC* | flagellar filament structural protein | GF_4133 | 3832.099 |
| **b2699** | *recA* | DNA recombination/repair protein RecA | GF_9596 | 3292.364 |
| **b2233** | *yfaL* | putative autotransporter adhesin YfaL | GF_2033 | 1821.806 |
| **b3359** | *argD* | N-acetylornithine aminotransferase/N-succinyldiaminopimelate aminotransferase | GF_4123 | 1812.858 |
| **b2231** | *gyrA* | DNA gyrase subunit A | GF_27077 | 1647.844 |
| **b4143** | *groL* | chaperonin GroEL | GF_5744 | 1497.186 |
| **b0957** | *ompA* | outer membrane protein A | GF_19292 | 1483.038 |
| **b0928** | *aspC* | aspartate aminotransferase | GF_10146 | 1449.186 |
| **b0095** | *ftsZ* | cell division protein FtsZ | GF_8531 | 1197.353 |
| **b0294** | *ecpR* | DNA-binding transcriptional dual regulator MatA | GF_4060 | 1183.701 |
| **b3987** | *rpoB* | RNA polymerase subunit beta | GF_10114 | 1126.995 |
| **b2741** | *rpoS* | RNA polymerase%2C sigma S (sigma 38) factor | GF_6615 | 1109.314 |
| **b4043** | *lexA* | DNA-binding transcriptional repressor LexA | GF_29695 | 1108.321 |
| **b3973** | *birA* | DNA-binding transcriptional repressor/biotin-[acetyl-CoA-carboxylase] ligase BirA | GF_10107 | 1106.663 |
| **b3699** | *gyrB* | DNA gyrase subunit B | GF_10402 | 1030.61 |
| **b0929** | *ompF* | outer membrane porin F | GF_7698 | 1024.15 |
| **b1617** | *uidA* | beta-glucuronidase | GF_3547 | 1016.759 |
| **b3702** | *dnaA* | chromosomal replication initiator protein DnaA | GF_10007 | 994.4677 |
| **b3067** | *rpoD* | RNA polymerase%2C sigma 70 (sigma D) factor | GF_12022 | 924.4761 |
| **b0842** | *mdfA* | multidrug efflux pump MdfA/Na(+):H(+) antiporter/K(+):H(+) antiporter | GF_8829 | 854.0526 |
| **b4150** | *ampC* | beta-lactamase | GF_2953 | 841.6712 |
| **b4024** | *lysC* | aspartate kinase III | GF_10132 | 790.144 |
| **b0383** | *phoA* | alkaline phosphatase | GF_8636 | 781.7246 |
| **b3342** | *rpsL* | 30S ribosomal subunit protein S12 | GF_29819 | 778.2261 |
| **b3035** | *tolC* | outer membrane channel TolC | GF_9745 | 739.9653 |
| **b3357** | *crp* | DNA-binding transcriptional dual regulator CRP | GF_29705 | 735.3363 |
| **b4058** | *uvrA* | excision nuclease subunit A | GF_10149 | 721.9311 |
| **b2945** | *endA* | DNA-specific endonuclease I | GF_9706 | 650.2455 |
| **b0611** | *rna* | RNase I | GF_4076 | 631.398 |

| | | | | |
|---|---|---|---|---|
| **b0064** | *araC* | DNA-binding transcriptional dual regulator AraC | GF_8514 | 610.5677 |
| **b4034** | *malE* | maltose ABC transporter periplasmic binding protein | GF_10138 | 608.5745 |
| **b4172** | *hfq* | RNA-binding protein Hfq | GF_29822 | 593.9419 |
| **b0624** | *crcB* | F(-) channel | GF_8724 | 581.1883 |
| **b4320** | *fimH* | type 1 fimbriae D-mannose specific adhesin | GF_29602 | 581.0575 |
| **b2215** | *ompC* | outer membrane porin C | GF_368 | 579.7966 |
| **b3822** | *recQ* | ATP-dependent DNA helicase RecQ | GF_28326 | 573.4825 |
| **b0888** | *trxB* | thioredoxin reductase | GF_20742 | 566.6851 |
| **b0565** | *ompT* | DLP12 prophage%3B protease 7 | GF_499 | 555.7283 |
| **b3806** | *cyaA* | adenylate cyclase | GF_24191 | 510.9377 |
| **b0533** | *sfmH* | putative fimbrial adhesin protein SfmH | GF_11601 | 510.0025 |
| **b4142** | *groS* | cochaperonin GroES | GF_4659 | 503.7996 |

**Supplementary Table S3**

**The total number of genes and publications together with the mapped softcore and publications for the six *E. coli* strains**

| taxon_id | STRING name | Genome Assembly ID | #Genes | #Publications | #Softcore | #Publications (Softcore) |
|---|---|---|---|---|---|---|
| 155864 | Escherichia coli O157H7 str. EDL933 | GCF_000732965.1 | 2874 | 3293 | 2069 | 2229 |
| 199310 | Escherichia coli CFT073 | GCF_000007445.1 | 1276 | 378 | 907 | 340 |
| 362663 | Escherichia coli 536 | GCF_000013305.1 | 391 | 115 | 322 | 110 |
| 469008 | Escherichia coli BL21 | GCF_000009565.1 | 1766 | 3153 | 1485 | 2981 |
| 481805 | Escherichia coli ATCC8739 | GCF_000019385.1 | 317 | 101 | 282 | 97 |
| 511145 | Escherichia coli K12 MG1655 | GCF_000005845.2 | 4097 | 171590 | 3011 | 158937 |
| | | | **Total (Union)** | **174120** | **3017** | **160598** |

**Supplementary Table S4**

**The number of *E. coli* softcore genes as well as sum of literature score in various FPE score ranges.**

We list the total number of softcore genes in the respective FPE range at the time of study ("#Genes"). We added a row for the 39 genes not specifically mentioned in any article about *E. coli*. Also, we calculated sum of the literature score for all genes in the respective FPE range ("Literature Score"). The total literature score is equivalent to the total number of articles identified in this study. The FPE score range is further classified into six categories and the total number of genes in that category is provided ("ΣGenes").

| FPE Score Range | #GFs | Percentage of 3056 GFs | Total Literature Score | Percentage of Total Score | ΣGFs | Category |
|---|---|---|---|---|---|---|
| 0 | 39 | 1.28% | 0 | 0.00% | 39 | Not studied |
| 0<x<1 | 336 | 10.99% | 118.12 | 0.07% | 1308 | Very understudied |
| 1≤x<5 | 586 | 19.18% | 1518.32 | 0.95% | | |
| 5≤x<10 | 386 | 12.63% | 2886.73 | 1.80% | | |
| 10≤x<15 | 279 | 9.13% | 3445.79 | 2.15% | 613 | Understudied |
| 15 ≤x< 20 | 183 | 5.99% | 3162.11 | 1.97% | | |
| 20 ≤x< 25 | 151 | 4.94% | 3373.96 | 2.10% | | |
| 25 ≤x< 30 | 119 | 3.89% | 3251.85 | 2.02% | 329 | Moderately studied |
| 30 ≤x< 35 | 102 | 3.34% | 3301.96 | 2.06% | | |
| 35≤x<40 | 108 | 3.53% | 4034.94 | 2.51% | | |
| 40≤x<45 | 63 | 2.06% | 2672.42 | 1.66% | 320 | Intensively studied |
| 45≤x<50 | 45 | 1.47% | 2129.00 | 1.33% | | |
| 50≤x<75 | 212 | 6.94% | 12813.19 | 7.98% | | |
| 75≤x<100 | 105 | 3.44% | 9131.59 | 5.69% | 447 | Very intensively studied |
| 100≤x<500 | 305 | 9.98% | 59455.68 | 37.02% | | |
| x≥500 | 37 | 1.21% | 49302.37 | 30.70% | | |
| **Total** | **3056** | **-** | **160598** | **-** | | |

**Supplementary Table S5**

**The growing trend of literature coverage for *E. coli* softcore genes in various FPE score thresholds**

The letter "T" in abbreviations "T0, T1, etc." stands for "threshold" applied to FPE values. Further, the curve of the number of new genes in the respective FPE range as a function of the year (see Supplementary Figure S4) is analysed with linear regression methods. The trend of changes is generally identified through two phases, *i. e.* Phase 1 and Phase 2. The slopes, $R^2$, ρ and P-value in time intervals of Phase 1 and Phase 2 are listed based on linear regression model $y_i \sim C + b.x_i$; where $y_i$ = total number of new genes reaching the specific FPE threshold at year *i*; $x_i$ = year *i*; *b* is the slope and *C* is intercept. The slope (*b*) indicates the rate of total number of new genes reaching a specific FPE score threshold throughout the years. A positive slope indicates that the total number of new genes reaching a specific FPE score threshold is larger than the previous year (or from year to year); a negative slope indicates otherwise. **ρ** is the linear correlation between the total number of new genes reaching a specific FPE score threshold and year. $R^2$ is the square of correlation or the goodness of fit of the linear regression. P-value is the significance of the slope. The total number of genes reaching the specific FPE score threshold can then be estimated by: $N_i \sim N_{(i-1)} + y_i$; where $N_i$ and $N_{(i-1)}$ = total number of genes reaching the specific FPE score threshold at year *i* and *(i-1)* respectively. The symbol ↑ indicates growing trend, whereas the symbol ↓ indicates declining trend. The symbol ↑↑ indicates accelerating growth trend.

| FPE Score Threshold | Phase 1 | | | | | Phase 2 | | | | |
|---|---|---|---|---|---|---|---|---|---|---|
| | Years | Slope | $R^2$ | ρ | P-value | Years | Slope | $R^2$ | ρ | P-value |
| 0 | - | - | - | - | - | - | - | - | - | - |
| T0 (0<x<1) | 1960 – 2009 ↑ | 1.39 | 0.76 | 0.87 | 3.55E-16 | 2009 – 2021 ↓ | -6.38 | 0.89 | 0.94 | 1.53E-06 |
| T1 (1≤x<5) | 1965 – 2009 ↑ | 1.21 | 0.68 | 0.82 | 3.34E-12 | 2009 – 2021 ↓ | -3.56 | 0.69 | 0.83 | 4.23E-04 |
| T5 (5≤x<10) | 1970 – 2013 ↑ | 1.48 | 0.83 | 0.91 | 5.67E-18 | 2013 – 2021 ↓ | -5.62 | 0.92 | 0.96 | 4.94E-05 |
| T10 (10≤x<15) | 1973 – 2001 ↑ | 0.97 | 0.78 | 0.88 | 2.09E-10 | 2001 – 2021 ↑↑ | 2.73 | 0.73 | 0.85 | 9.62E-07 |
| T15 (15 ≤x< 20) | 1973 – 2003 ↑ | 0.84 | 0.83 | 0.91 | 7.13E-12 | 2003 – 2021 ↑↑ | 2.91 | 0.65 | 0.81 | 3.21E-05 |
| T20 (20 ≤x< 25) | 1973 – 2004 ↑ | 0.64 | 0.77 | 0.88 | 3.68E-11 | 2004 – 2021 ↑↑ | 3.16 | 0.84 | 0.92 | 8.47E-08 |
| T25 (25 ≤x< 30) | 1975 – 2004 ↑ | 0.48 | 0.64 | 0.80 | 1.36E-07 | 2004 – 2021 ↑↑ | 3.33 | 0.87 | 0.93 | 1.89E-08 |
| T30 (30 ≤x< 35) | 1975 – 2004 ↑ | 0.47 | 0.71 | 0.84 | 5.67E-09 | 2004 – 2021 ↑↑ | 3.26 | 0.88 | 0.94 | 6.85E-09 |
| T35 (35≤x<40) | 1975 – 2004 ↑ | 0.40 | 0.77 | 0.88 | 1.46E-10 | 2004 – 2021 ↑↑ | 2.91 | 0.85 | 0.92 | 5.08E-08 |
| T40 (40≤x<45) | 1975 – 2006 ↑ | 0.38 | 0.66 | 0.81 | 1.75E-08 | 2006 – 2021 ↑↑ | 2.22 | 0.83 | 0.91 | 1.07E-06 |

| | | | | | | | | | |
|---|---|---|---|---|---|---|---|---|---|
| T45 (45≤x<50) | 1975 – 2006 ↑ | 0.35 | 0.59 | 0.77 | 2.45E-07 | 2006 – 2021 ↑↑ | 2.08 | 0.71 | 0.85 | 3.75E-05 |
| T50 (50≤x<75) | 1975 – 2006 ↑ | 0.34 | 0.70 | 0.84 | 2.09E-09 | 2006 – 2021 ↑↑ | 2.53 | 0.77 | 0.88 | 8.33E-06 |
| T75 (75≤x<100) | 1980 – 2006 ↑ | 0.17 | 0.39 | 0.63 | 4.78E-04 | 2006 – 2021 ↑↑ | 2.03 | 0.85 | 0.92 | 3.62E-07 |
| T100 (100≤x<500) | 1980 – 2006 ↑ | 0.10 | 0.23 | 0.48 | 1.09E-02 | 2006 – 2021 ↑↑ | 1.86 | 0.77 | 0.88 | 7.19E-06 |
| T500 (x≥500) | 1980 – 2021 ↑ | 0.08 | 0.42 | 0.64 | 4.14E-06 | - | - | - | - | - |

**Supplementary Table S6**

**Exclusion lists of genes from the group of 176 K-12 MG1655 genes.**

176 *E. coli* K-12 MG1655 genes are left without any assigned literature by our automated procedure (Supplementary File 1). When mapped to the GFs constituting the *E. coli* pan-genome, they are found contained in 171 GFs (see upper part of the table). Four GFs include several genes). The gene b4795/*yibX* has two transcripts that match different GFs. We checked if any of the genes in those 171 GFs mapped to a publication with our automated procedure. We were able to find articles for 11 genes via their homologues (see lower part of the table).

Supplementary File 6 lists the remaining 160 GFs together with the 11 in this list. 36 GFs belong to the 95%-threshold softcore genome. GF_10343 with b3782/*rhoL* is even part of the core genome. This suggests that there are still genes within the *E. coli* genome with fundamental function, widely distributed among lineages but not well studied.

| Gene family No. in the *E. coli* pangenome | Genes from K-12 MG1655 contained in the gene family |
|---|---|
| GF_1516 | *ymcF* and *ynfQ* |
| GF_10366 | *yibX* (YP_010051208.1, 80AA) |
| GF_10367 | *yibX* (YP_010051209.1, 24AA) |
| GF_10369 | *ymgK* and *yicU* |
| GF_10438 | *ynaM* and *ynfT* |
| GF_17432 | *ibsA*, *ibsC*, *ibsD* and *ibsE* |
| GF_6 | *yagB, yafW, cbeA* and *yfjZ* |
| GF_109 | *yfjQ* and *yafZ* |
| GF_140 | *nohA* and *nohD* |
| GF_184 | *tfaD*, *tfaR* and *tfaQ* |
| GF_911 | *ldrA*, *ldrB*, *ldrC* and *ldrD* |
| GF_1072 | *insH21*, *insH1*, *insH2*, *insH3*, *insH4*, *insH5*, *insH6*, *insH7*, *insH8*, *insH9*, *insH10* and *insH11* |
| GF_2454 | *insB1*, *insB2*, *insB3*, *insB4*, *insB5*, *insB6* and *insB9* |
| GF_8211 | *yiaV* and *yibH* |
| GF_8507 | *ydiR* and *fixB* |

| | |
|---|---|
| GF_10234 | *insA1*, *insA2*, *insA3*, *insA4*, *insA5*, *insA6*, *insA7* and *insA9* |
| GF_10286 | *yabR* and *azuC* |

**Supplementary Table S7**

**The functional code description of the COG reference database.** None of the genes in E. coli K-12 MG1655 is annotated with functional code B, Y and Z; therefore excluded (~~strikethrough~~).

| Functional Code | Functional Description |
|---|---|
| **A** | RNA processing and modification |
| ~~**B**~~ | ~~Chromatin structure and dynamics~~ |
| **C** | Energy production and conversion |
| **D** | Cell cycle control, cell division, chromosome partitioning |
| **E** | Amino acid transport and metabolism |
| **F** | Nucleotide transport and metabolism |
| **G** | Carbohydrate transport and metabolism |
| **H** | Coenzyme transport and metabolism |
| **I** | Lipid transport and metabolism |
| **J** | Translation, ribosomal structure, and biogenesis |
| **K** | Transcription |
| **L** | Replication, recombination, and repair |
| **M** | Cell wall/membrane/envelope biogenesis |
| **N** | Cell motility |
| **O** | Posttranslational modification, protein turnover, chaperones |
| **P** | Inorganic ion transport and metabolism |
| **Q** | Secondary metabolites biosynthesis, transport, and catabolism |
| **R** | General function prediction only |
| **S** | Function unknown |
| **T** | Signal transduction mechanisms |
| **U** | Intracellular trafficking, secretion, and vesicular transport |
| **V** | Defense mechanisms |
| **W** | Extracellular structures |
| **X** | Mobilome: prophages, transposons |
| ~~**Y**~~ | ~~Nuclear structure~~ |
| ~~**Z**~~ | ~~Cytoskeleton~~ |
