# Supplementary material for: About the dark corners in the gene function space of Escherichia coli remaining without illumination by scientific literature
Source: Biol Direct. 2023 Feb 28;18:7. doi: 10.1186/s13062-023-00362-0 (PMC9976479; doi:10.1186/s13062-023-00362-0)
Supplement: Supplementary file 2 — Additional file 2: Fig. S1. We show the total number of E. coli softcore genes’ related publications (red line relative to the left y-axis) and the total number of genes mentioned in the respective literature (blue line relative to the right y-axis) from year 1939 up to year 2021. The blue dashed vertical lines mark the expansion period for the total number of genes from year 1965 to 2009. It apparently plateaus after the year 2019. The red dashed vertical lines at years 1970 and 2007 indicate two periods of publication dynamics: 1970–2007 and 2007–2021. The ratio of the number of publications in each year to the total number of new genes identified in each year is shown in the insert. Fig. S2. FPE plots for different FPE score ranges from year 1960 until 2021 for E. coli K-12 genes are separately shown for five different categories, i.e. (A) very understudied, (B) understudied, (C) moderately studied, (D) intensively studied and (E) very intensively studied. The y-axis is given in the same scale for visual comparison across different categories. Fig. S3. We illustrate the number of new genes of E. coli K-12 achieving the FPE score ranges (T0, T1, T5, T10, T15, T20, T25, T30, T35, T40, T45, T50, T75, T100, T500) across the years in (A) phase 1 and (B) phase 2 periods. The linear regression line (number of new genes (y-axis) versus year (x-axis)) is shown. The magnitude of the slope is provided in Table 2. Fig. S4. FPE plots for different FPE score range from year 1960 until 2021 for E. coli softcore genes are separately shown for five different categories, i.e. (A) very understudied, (B) understudied, (C) moderately studied, (D) intensively studied and (E) very intensively studied. The y-axis is given in the same scale for visual comparison across different categories. Fig. S5. We illustrate the number of new genes of the E. coli softcore genome achieving the FPE score ranges (T0, T1, T5, T10, T15, T20, T25, T30, T35, T40, T45, T50, T75, T100, T500) across the years [file 13062_2023_362_MOESM2_ESM.zip › Legends to Supplementary Figures.pdf]

Supplementary Figure S1

We show the total number of *E. coli* softcore genes' related publications (red line relative to the left y-axis) and the total number of genes mentioned in the respective literature (blue line relative to the right y-axis) from year 1939 up to year 2021. The blue dashed vertical lines mark the expansion period for the total number of genes from year 1965 to 2009. It apparently plateaus after the year 2019. The red dashed vertical lines at years 1970 and 2007 indicate two periods of publication dynamics: 1970-2007 and 2007-2021. The ratio of the number of publications in each year to the total number of new genes identified in each year is shown in the insert.

Supplementary Figure S2

FPE plots for different FPE score ranges from year 1960 until 2021 for *E. coli* K-12 genes are separately shown for five different categories, i.e. (A) very understudied, (B) understudied, (C) moderately studied, (D) intensively studied and (E) very intensively studied. The y-axis is given in the same scale for visual comparison across different categories.

Supplementary Figure S3

We illustrate the number of new genes of *E. coli* K-12 achieving the FPE score ranges (T0, T1, T5, T10, T15, T20, T25, T30, T35, T40, T45, T50, T75, T100, T500) across the years in (A) phase 1 and (B) phase 2 periods. The linear regression line (number of new genes (y-axis) versus year (x-axis)) is shown. The magnitude of the slope is provided in Table 2.

Supplementary Figure S4

FPE plots for different FPE score range from year 1960 until 2021 for *E. coli* softcore genes are separately shown for five different categories, i.e. (A) very understudied, (B) understudied, (C) moderately studied, (D) intensively studied and (E) very intensively studied. The y-axis is given in the same scale for visual comparison across different categories.

Supplementary Figure S5

We illustrate the number of new genes of the *E. coli* softcore genome achieving the FPE score ranges (T0, T1, T5, T10, T15, T20, T25, T30, T35, T40, T45, T50, T75, T100, T500) across the years in (A) phase 1 and (B) phase 2 periods. The linear regression line (number of new genes (y-axis) versus year (x-axis)) is shown. The magnitude of the slope is provided in Supplementary Table S5.

Supplementary Figure S6

Prediction of the transmembrane (TM) region in the protein sequence *yahV* (GF_29643) in *E. coli* K-12 MG1655 using TMHMM 2.0. The TM region is predicted to cover positions 4-23 of the protein sequence.

Supplementary Figure S7

The upstream and downstream genes of *yahV* based on NCBI RefSeq. The *betABIT* operon is upstream of *yahV* gene. *betABIT* is expressed only under aerobic condition during osmotic stress

for production of osmoprotectants. The *pdeL* gene, on the other hand, is downstream of the gene *yahV*. The *pdeL* gene appears involved in the regulation of cell motility.

Supplementary Figure S8

Neighboring gene families of GF_29643 (*yahV*; circled in red) focusing on genomes that carry GF_29643. Ten GFs upstream and ten GFs downstream of GF_29643 are extracted and investigated. Each GF is represented as a node and two nodes are linked by an edge if they are next to each other. The thickness of the edge represents the weighted link between the two GFs. Clearly, GF_29643's genomic position is conserved across the *E. coli* genomes that carry the *yahV* gene. Note that GF_8617 represents the *betT* gene and GF_25808 contains the *pdeL* gene.

Supplementary Figure S9

The predicted transmembrane beta-barrel (TMBB) structure of protein *yddL* (GF_4841) using BetAware-Deep. The predicted localization is outer membrane TMBB with the overall TMBB probability of 0.93. There are four (4) TM β-strand segments as shown in the figure.

Supplementary Figure S10

We illustrate the GFs associated with GF_29643, GF_4841 and GF_8394. The associated GFs of these three GFs have high overlap with each other and, therefore, can be related. Each node represents a GF and the edge (connecting line) indicates a significant coincident association between nodes (P-value $\leq 1 \times 10^{-20}$). The size of the node is determined by the node's degree (the number of associated GFs). The color of the node is represented by a gradient color from grey to red which is determined by the node's degree as well. The three cluster-founding GFs are highlighted by red arrows. Please note that only 60 out of 68 GFs found are present in *E. coli* K-12 MG1655.

Supplementary Figure S11

The number of overlapping associated GFs among three GFs, i.e., GF_29643, GF_8394 and GF_4841.

Supplementary Figure S12

Manual annotation of associated GFs to GF_29643 (*yahV)*, GF_4841 (*yddL)*, and GF_8394 (*paaE*). There are four potential biological processes related to these 3 GFs, i.e. osmotic regulation, stress response, cell motility and energy metabolism. The corresponding genes are given for each biological process. The genes with unclear function are given as "Not Clear".

Supplementary Figure S13

The protein expression of 11 genes extracted from Caglar's proteomics data. Only 11 genes out of 30 gene families, which are fully connected or significantly associated to each other, have the protein expression in Caglar's proteomics data. Please note that the *E. coli* strain used in Caglar's study is *E. coli* REL606, which belongs to phylogroup A (sequence type ST93). This is different from *E. coli* K-12 MG1655, which has sequence type ST10. The highlighted box (with a red dashed line) emphasizes the expression results from cultures under NaCl_Stress condition.

Supplementary Figure S14

We visualize the gene expression of 19 genes extracted from the Metris *et al*. data in accordance with osmotic conditions. These 19 genes are from our set of 30 GFs, which are fully connected or significantly associated to each other. Please note that the *E. coli* strain used in Metris' study is *E. coli* K12 MG1655, which is the same as the *E. coli* strain in our analysis.
