# Supplementary figures and images for: About the dark corners in the gene function space of Escherichia coli remaining without illumination by scientific literature
Source: Biol Direct. 2023 Feb 28;18:7. doi: 10.1186/s13062-023-00362-0 (PMC9976479; doi:10.1186/s13062-023-00362-0)

Number of Publications

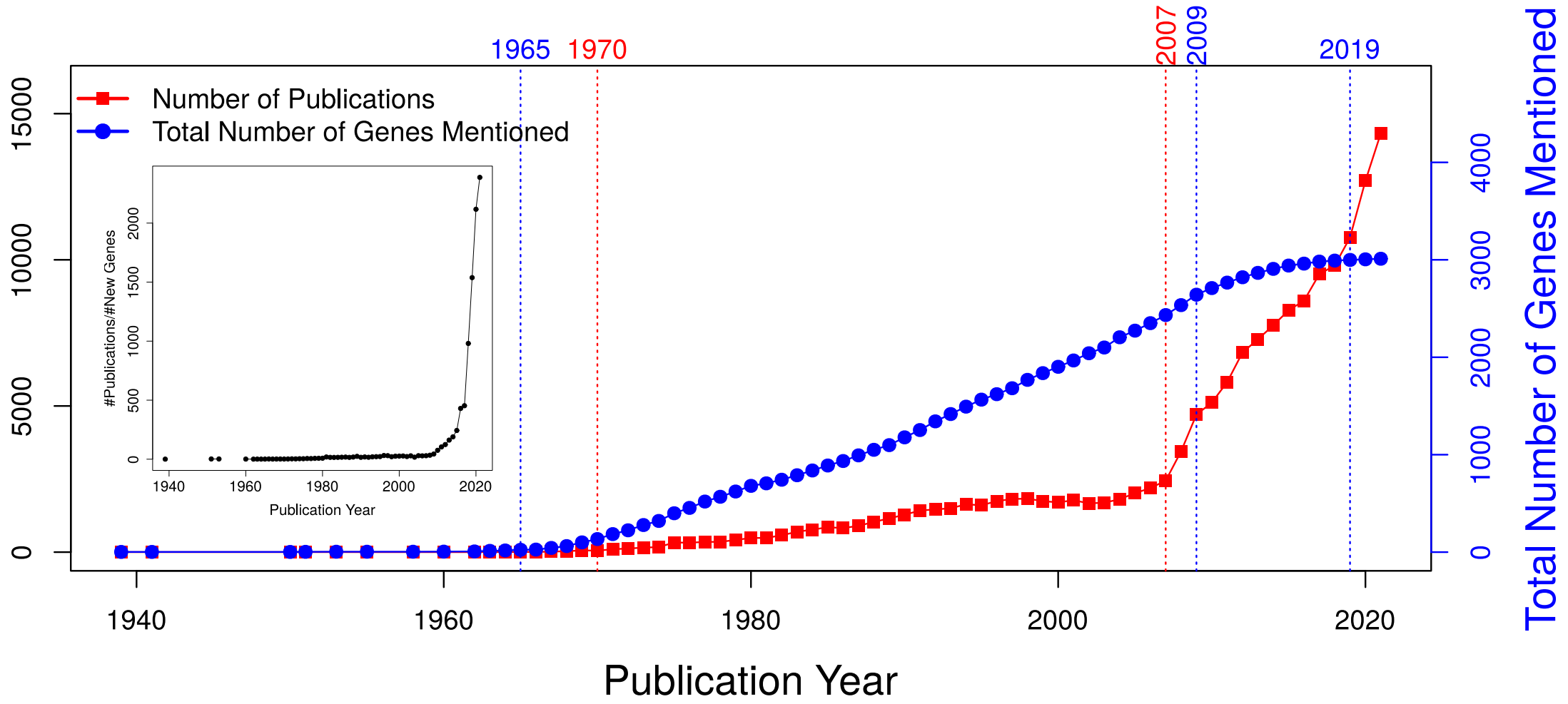

Supplement: Supplementary file 2 — Additional file 2: Fig. S1. We show the total number of E. coli softcore genes’ related publications (red line relative to the left y-axis) and the total number of genes mentioned in the respective literature (blue line relative to the right y-axis) from year 1939 up to year 2021. The blue dashed vertical lines mark the expansion period for the total number of genes from year 1965 to 2009. It apparently plateaus after the year 2019. The red dashed vertical lines at years 1970 and 2007 indicate two periods of publication dynamics: 1970–2007 and 2007–2021. The ratio of the number of publications in each year to the total number of new genes identified in each year is shown in the insert. Fig. S2. FPE plots for different FPE score ranges from year 1960 until 2021 for E. coli K-12 genes are separately shown for five different categories, i.e. (A) very understudied, (B) understudied, (C) moderately studied, (D) intensively studied and (E) very intensively studied. The y-axis is given in the same scale for visual comparison across different categories. Fig. S3. We illustrate the number of new genes of E. coli K-12 achieving the FPE score ranges (T0, T1, T5, T10, T15, T20, T25, T30, T35, T40, T45, T50, T75, T100, T500) across the years in (A) phase 1 and (B) phase 2 periods. The linear regression line (number of new genes (y-axis) versus year (x-axis)) is shown. The magnitude of the slope is provided in Table 2. Fig. S4. FPE plots for different FPE score range from year 1960 until 2021 for E. coli softcore genes are separately shown for five different categories, i.e. (A) very understudied, (B) understudied, (C) moderately studied, (D) intensively studied and (E) very intensively studied. The y-axis is given in the same scale for visual comparison across different categories. Fig. S5. We illustrate the number of new genes of the E. coli softcore genome achieving the FPE score ranges (T0, T1, T5, T10, T15, T20, T25, T30, T35, T40, T45, T50, T75, T100, T500) across the years [file 13062_2023_362_MOESM2_ESM.zip › Supplementary Figure S1.pdf]

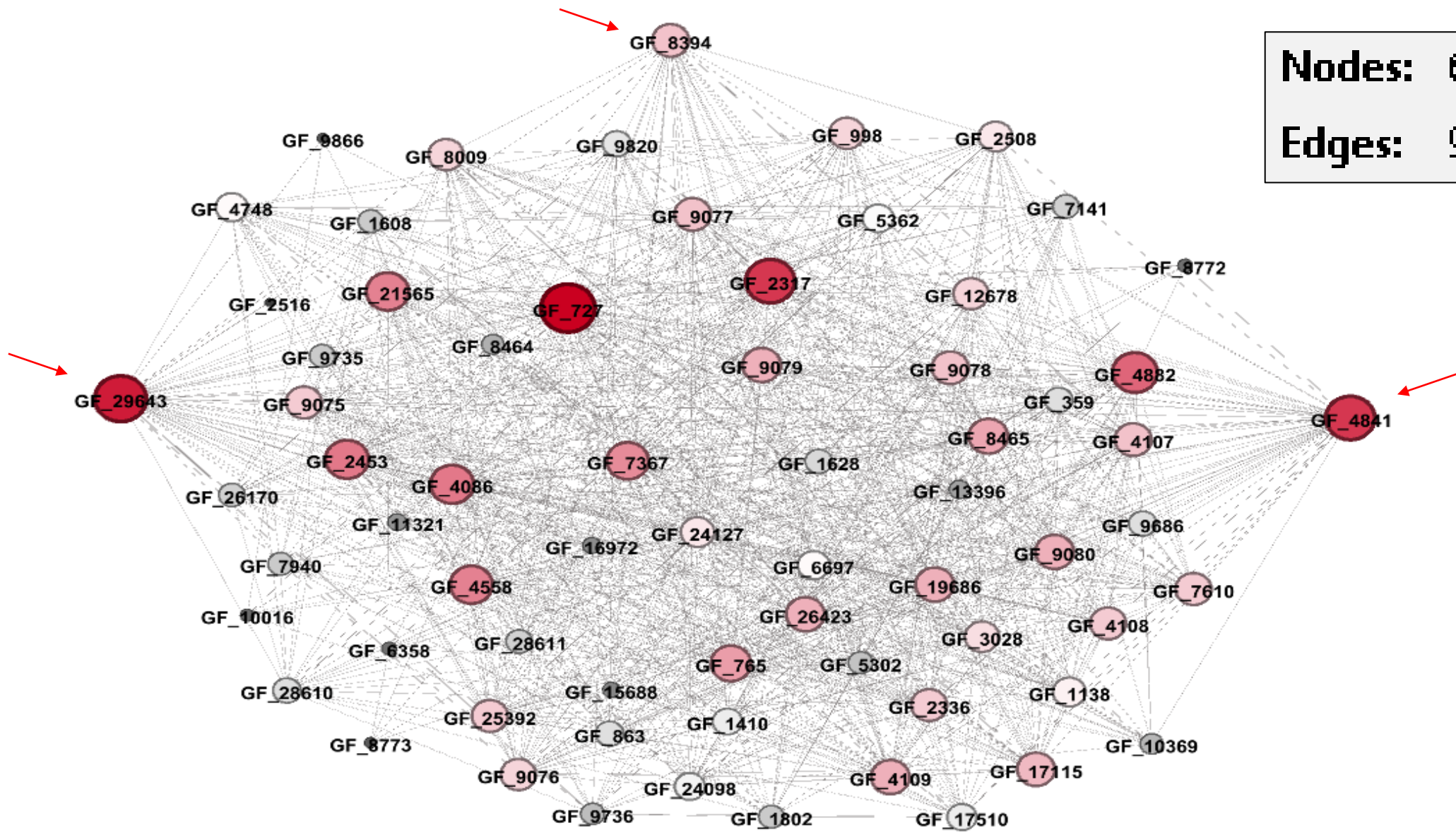

**Nodes:** 68

**Edges:** 973

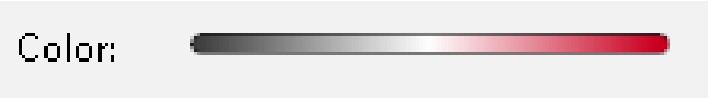

Supplement: Supplementary file 2 — Additional file 2: Fig. S1. We show the total number of E. coli softcore genes’ related publications (red line relative to the left y-axis) and the total number of genes mentioned in the respective literature (blue line relative to the right y-axis) from year 1939 up to year 2021. The blue dashed vertical lines mark the expansion period for the total number of genes from year 1965 to 2009. It apparently plateaus after the year 2019. The red dashed vertical lines at years 1970 and 2007 indicate two periods of publication dynamics: 1970–2007 and 2007–2021. The ratio of the number of publications in each year to the total number of new genes identified in each year is shown in the insert. Fig. S2. FPE plots for different FPE score ranges from year 1960 until 2021 for E. coli K-12 genes are separately shown for five different categories, i.e. (A) very understudied, (B) understudied, (C) moderately studied, (D) intensively studied and (E) very intensively studied. The y-axis is given in the same scale for visual comparison across different categories. Fig. S3. We illustrate the number of new genes of E. coli K-12 achieving the FPE score ranges (T0, T1, T5, T10, T15, T20, T25, T30, T35, T40, T45, T50, T75, T100, T500) across the years in (A) phase 1 and (B) phase 2 periods. The linear regression line (number of new genes (y-axis) versus year (x-axis)) is shown. The magnitude of the slope is provided in Table 2. Fig. S4. FPE plots for different FPE score range from year 1960 until 2021 for E. coli softcore genes are separately shown for five different categories, i.e. (A) very understudied, (B) understudied, (C) moderately studied, (D) intensively studied and (E) very intensively studied. The y-axis is given in the same scale for visual comparison across different categories. Fig. S5. We illustrate the number of new genes of the E. coli softcore genome achieving the FPE score ranges (T0, T1, T5, T10, T15, T20, T25, T30, T35, T40, T45, T50, T75, T100, T500) across the years [file 13062_2023_362_MOESM2_ESM.zip › Supplementary Figure S10.pdf]

GF\_29643

GF\_8394

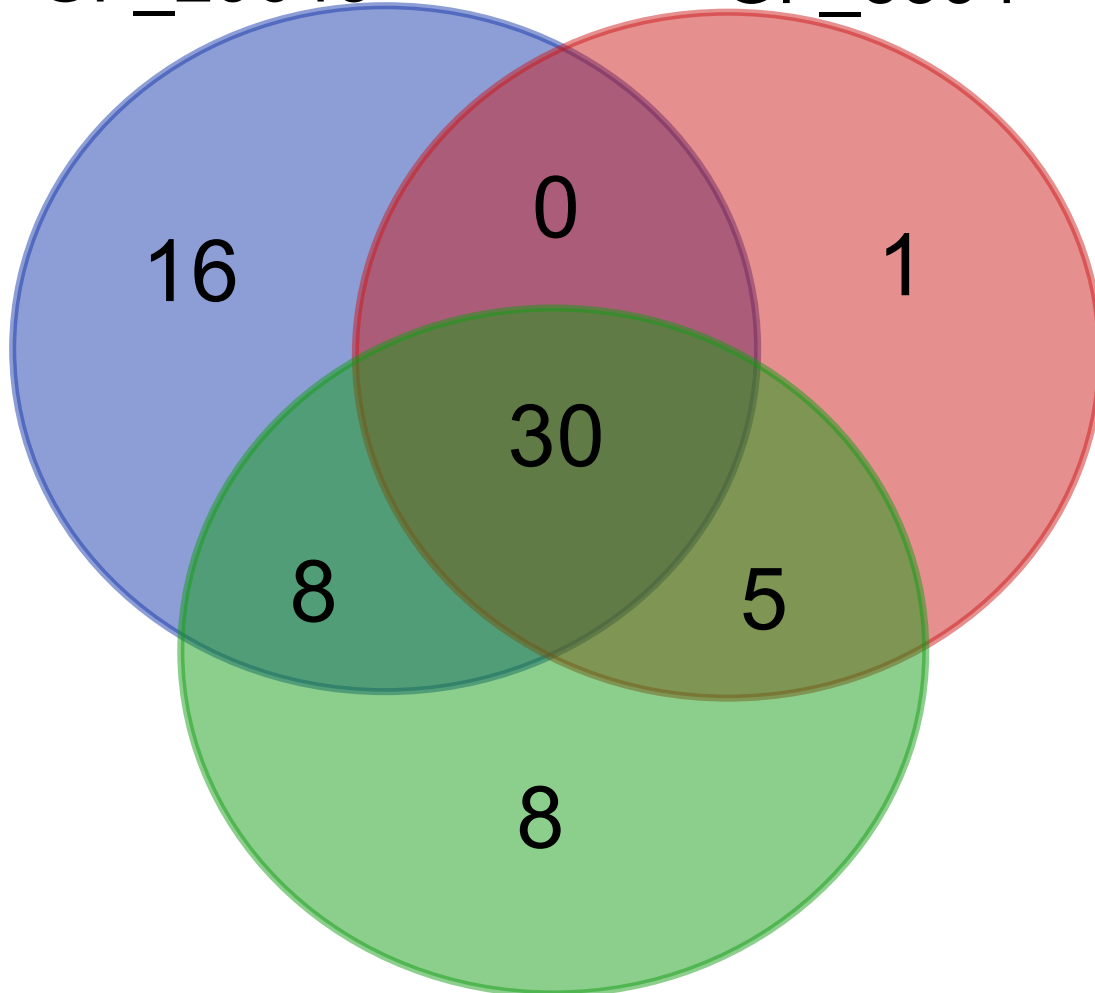

GF\_4841

Supplement: Supplementary file 2 — Additional file 2: Fig. S1. We show the total number of E. coli softcore genes’ related publications (red line relative to the left y-axis) and the total number of genes mentioned in the respective literature (blue line relative to the right y-axis) from year 1939 up to year 2021. The blue dashed vertical lines mark the expansion period for the total number of genes from year 1965 to 2009. It apparently plateaus after the year 2019. The red dashed vertical lines at years 1970 and 2007 indicate two periods of publication dynamics: 1970–2007 and 2007–2021. The ratio of the number of publications in each year to the total number of new genes identified in each year is shown in the insert. Fig. S2. FPE plots for different FPE score ranges from year 1960 until 2021 for E. coli K-12 genes are separately shown for five different categories, i.e. (A) very understudied, (B) understudied, (C) moderately studied, (D) intensively studied and (E) very intensively studied. The y-axis is given in the same scale for visual comparison across different categories. Fig. S3. We illustrate the number of new genes of E. coli K-12 achieving the FPE score ranges (T0, T1, T5, T10, T15, T20, T25, T30, T35, T40, T45, T50, T75, T100, T500) across the years in (A) phase 1 and (B) phase 2 periods. The linear regression line (number of new genes (y-axis) versus year (x-axis)) is shown. The magnitude of the slope is provided in Table 2. Fig. S4. FPE plots for different FPE score range from year 1960 until 2021 for E. coli softcore genes are separately shown for five different categories, i.e. (A) very understudied, (B) understudied, (C) moderately studied, (D) intensively studied and (E) very intensively studied. The y-axis is given in the same scale for visual comparison across different categories. Fig. S5. We illustrate the number of new genes of the E. coli softcore genome achieving the FPE score ranges (T0, T1, T5, T10, T15, T20, T25, T30, T35, T40, T45, T50, T75, T100, T500) across the years [file 13062_2023_362_MOESM2_ESM.zip › Supplementary Figure S11.pdf]

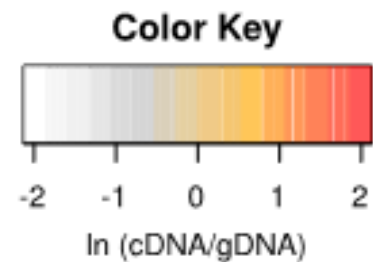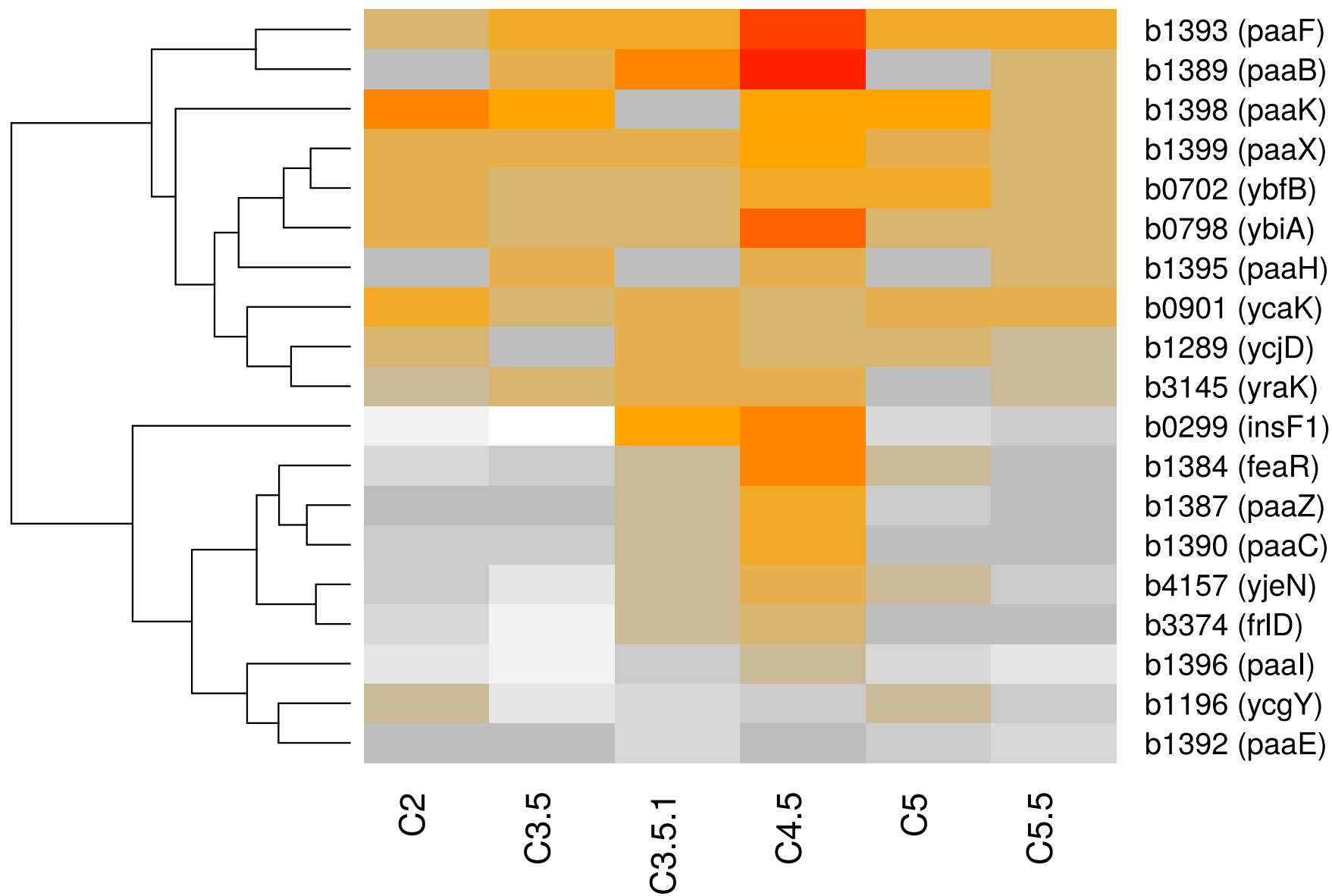

Supplement: Supplementary file 2 — Additional file 2: Fig. S1. We show the total number of E. coli softcore genes’ related publications (red line relative to the left y-axis) and the total number of genes mentioned in the respective literature (blue line relative to the right y-axis) from year 1939 up to year 2021. The blue dashed vertical lines mark the expansion period for the total number of genes from year 1965 to 2009. It apparently plateaus after the year 2019. The red dashed vertical lines at years 1970 and 2007 indicate two periods of publication dynamics: 1970–2007 and 2007–2021. The ratio of the number of publications in each year to the total number of new genes identified in each year is shown in the insert. Fig. S2. FPE plots for different FPE score ranges from year 1960 until 2021 for E. coli K-12 genes are separately shown for five different categories, i.e. (A) very understudied, (B) understudied, (C) moderately studied, (D) intensively studied and (E) very intensively studied. The y-axis is given in the same scale for visual comparison across different categories. Fig. S3. We illustrate the number of new genes of E. coli K-12 achieving the FPE score ranges (T0, T1, T5, T10, T15, T20, T25, T30, T35, T40, T45, T50, T75, T100, T500) across the years in (A) phase 1 and (B) phase 2 periods. The linear regression line (number of new genes (y-axis) versus year (x-axis)) is shown. The magnitude of the slope is provided in Table 2. Fig. S4. FPE plots for different FPE score range from year 1960 until 2021 for E. coli softcore genes are separately shown for five different categories, i.e. (A) very understudied, (B) understudied, (C) moderately studied, (D) intensively studied and (E) very intensively studied. The y-axis is given in the same scale for visual comparison across different categories. Fig. S5. We illustrate the number of new genes of the E. coli softcore genome achieving the FPE score ranges (T0, T1, T5, T10, T15, T20, T25, T30, T35, T40, T45, T50, T75, T100, T500) across the years [file 13062_2023_362_MOESM2_ESM.zip › Supplementary Figure S14.pdf]

A

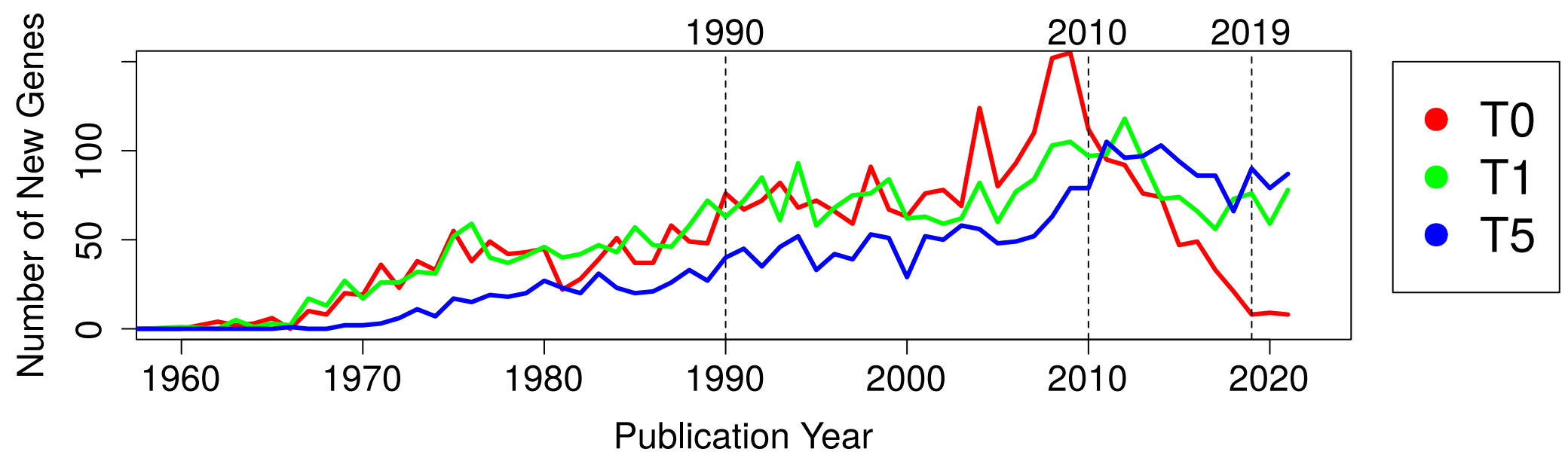

B

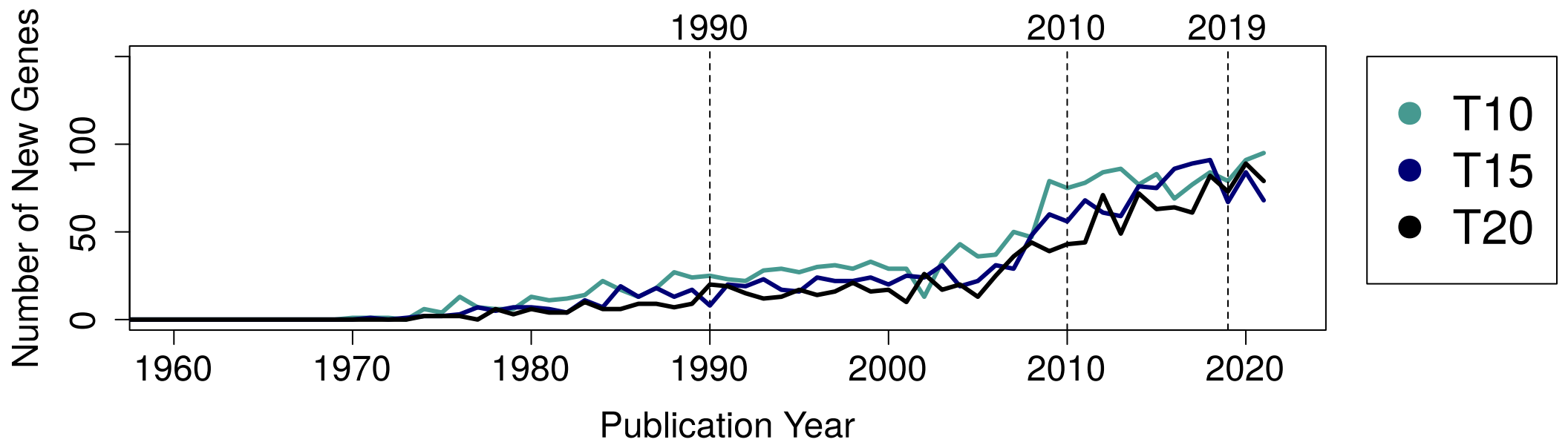

C

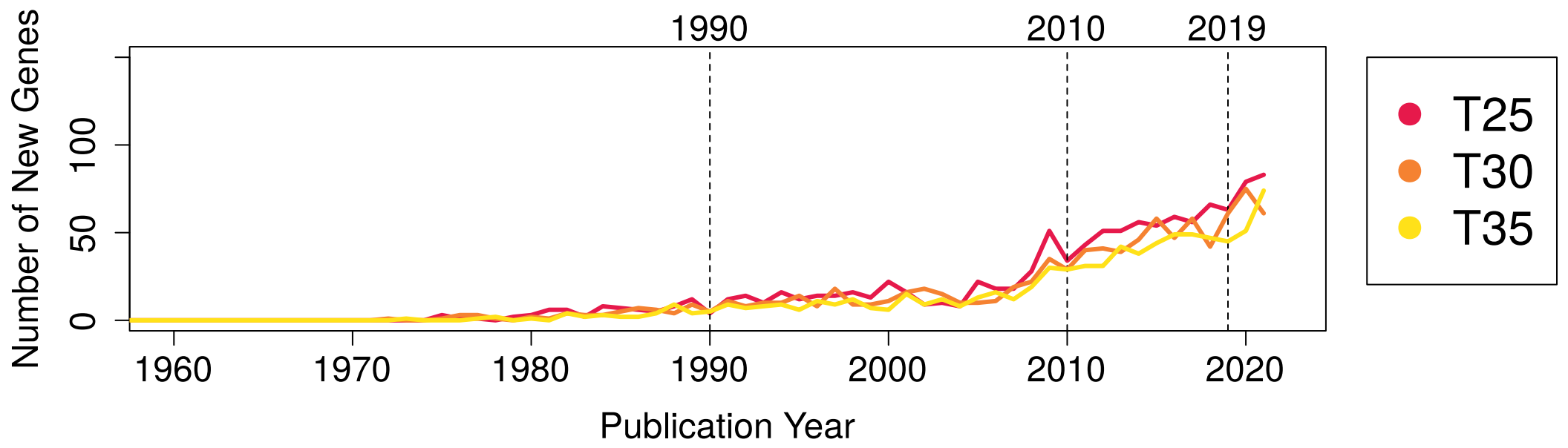

D

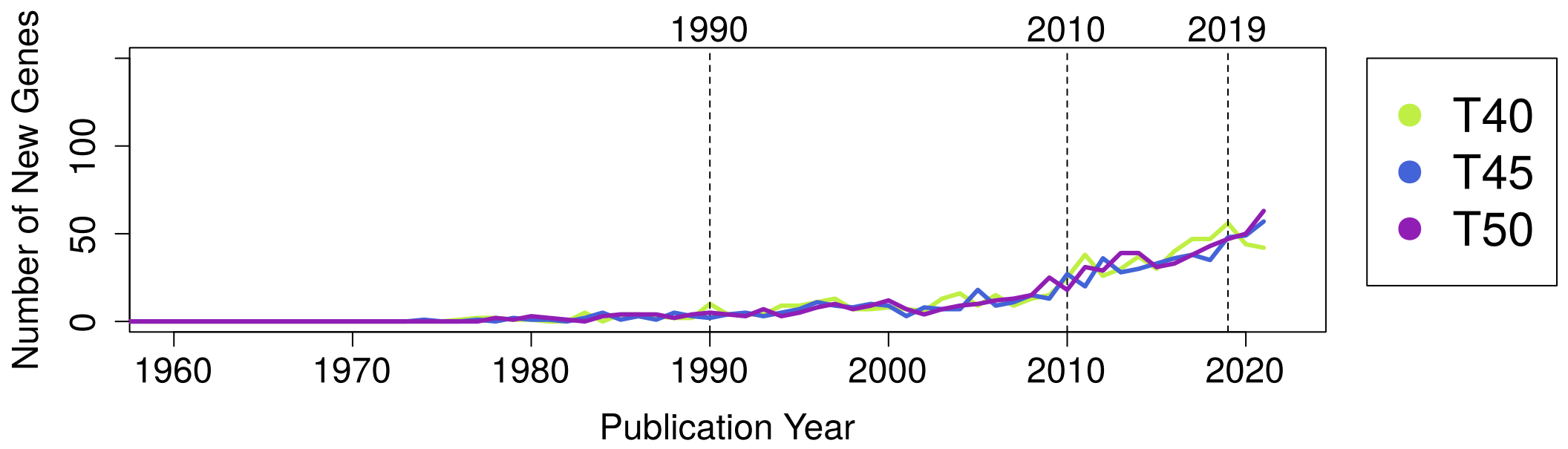

E

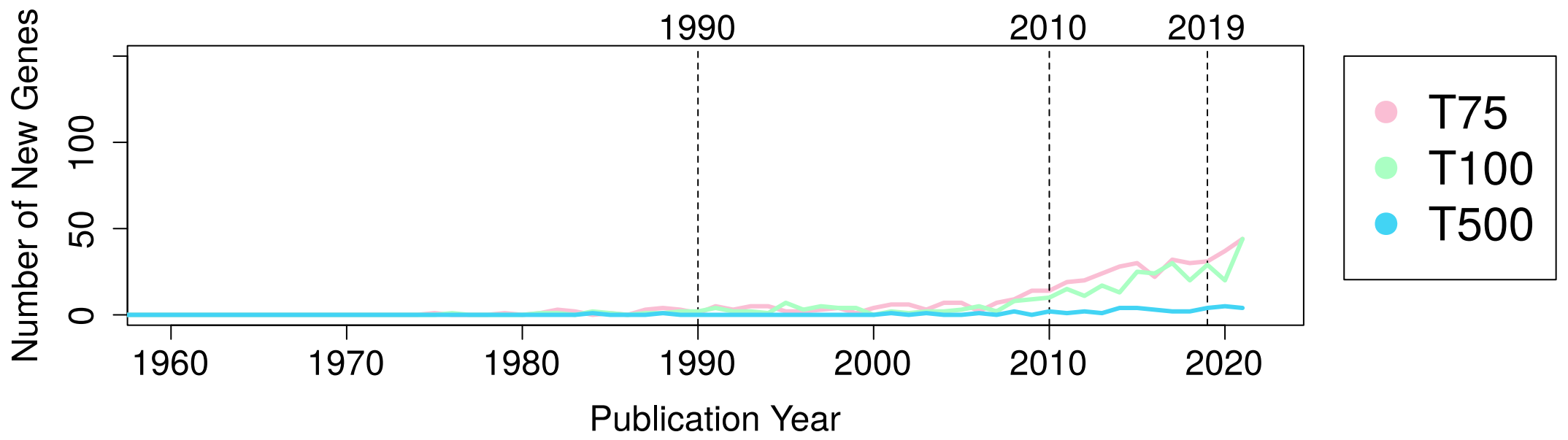

Supplement: Supplementary file 2 — Additional file 2: Fig. S1. We show the total number of E. coli softcore genes’ related publications (red line relative to the left y-axis) and the total number of genes mentioned in the respective literature (blue line relative to the right y-axis) from year 1939 up to year 2021. The blue dashed vertical lines mark the expansion period for the total number of genes from year 1965 to 2009. It apparently plateaus after the year 2019. The red dashed vertical lines at years 1970 and 2007 indicate two periods of publication dynamics: 1970–2007 and 2007–2021. The ratio of the number of publications in each year to the total number of new genes identified in each year is shown in the insert. Fig. S2. FPE plots for different FPE score ranges from year 1960 until 2021 for E. coli K-12 genes are separately shown for five different categories, i.e. (A) very understudied, (B) understudied, (C) moderately studied, (D) intensively studied and (E) very intensively studied. The y-axis is given in the same scale for visual comparison across different categories. Fig. S3. We illustrate the number of new genes of E. coli K-12 achieving the FPE score ranges (T0, T1, T5, T10, T15, T20, T25, T30, T35, T40, T45, T50, T75, T100, T500) across the years in (A) phase 1 and (B) phase 2 periods. The linear regression line (number of new genes (y-axis) versus year (x-axis)) is shown. The magnitude of the slope is provided in Table 2. Fig. S4. FPE plots for different FPE score range from year 1960 until 2021 for E. coli softcore genes are separately shown for five different categories, i.e. (A) very understudied, (B) understudied, (C) moderately studied, (D) intensively studied and (E) very intensively studied. The y-axis is given in the same scale for visual comparison across different categories. Fig. S5. We illustrate the number of new genes of the E. coli softcore genome achieving the FPE score ranges (T0, T1, T5, T10, T15, T20, T25, T30, T35, T40, T45, T50, T75, T100, T500) across the years [file 13062_2023_362_MOESM2_ESM.zip › Supplementary Figure S2.pdf]

**A**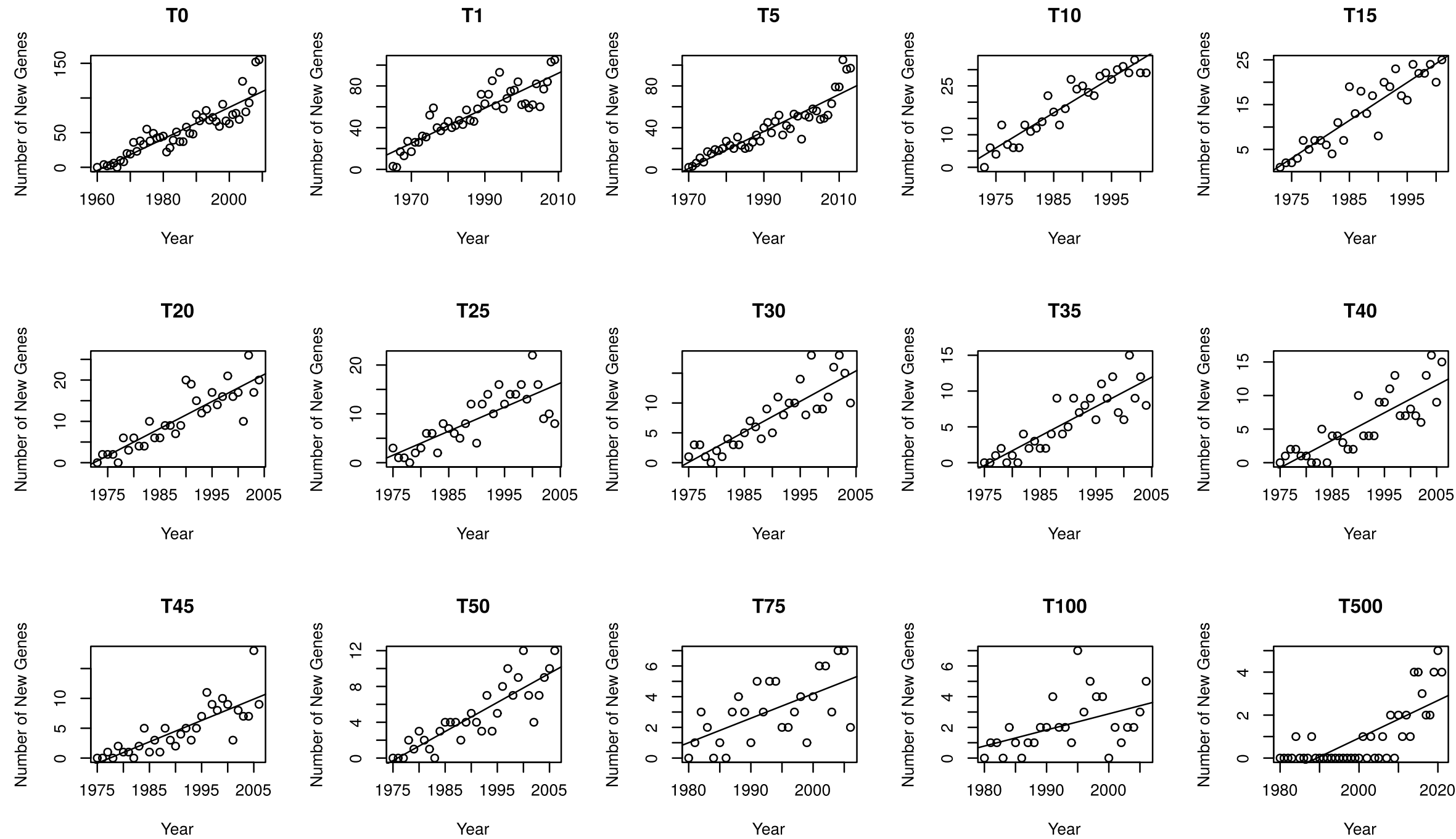**B**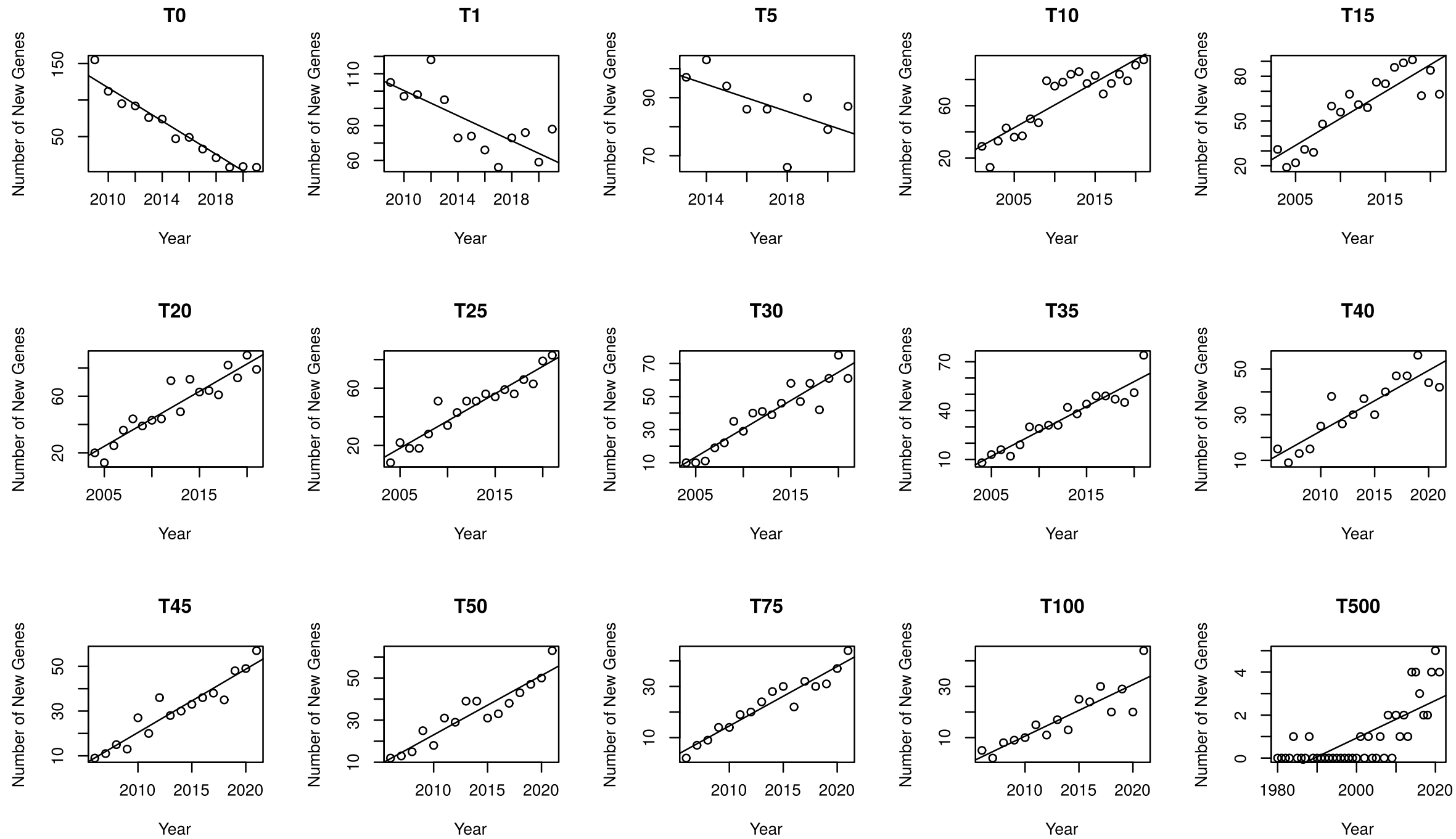

Supplement: Supplementary file 2 — Additional file 2: Fig. S1. We show the total number of E. coli softcore genes’ related publications (red line relative to the left y-axis) and the total number of genes mentioned in the respective literature (blue line relative to the right y-axis) from year 1939 up to year 2021. The blue dashed vertical lines mark the expansion period for the total number of genes from year 1965 to 2009. It apparently plateaus after the year 2019. The red dashed vertical lines at years 1970 and 2007 indicate two periods of publication dynamics: 1970–2007 and 2007–2021. The ratio of the number of publications in each year to the total number of new genes identified in each year is shown in the insert. Fig. S2. FPE plots for different FPE score ranges from year 1960 until 2021 for E. coli K-12 genes are separately shown for five different categories, i.e. (A) very understudied, (B) understudied, (C) moderately studied, (D) intensively studied and (E) very intensively studied. The y-axis is given in the same scale for visual comparison across different categories. Fig. S3. We illustrate the number of new genes of E. coli K-12 achieving the FPE score ranges (T0, T1, T5, T10, T15, T20, T25, T30, T35, T40, T45, T50, T75, T100, T500) across the years in (A) phase 1 and (B) phase 2 periods. The linear regression line (number of new genes (y-axis) versus year (x-axis)) is shown. The magnitude of the slope is provided in Table 2. Fig. S4. FPE plots for different FPE score range from year 1960 until 2021 for E. coli softcore genes are separately shown for five different categories, i.e. (A) very understudied, (B) understudied, (C) moderately studied, (D) intensively studied and (E) very intensively studied. The y-axis is given in the same scale for visual comparison across different categories. Fig. S5. We illustrate the number of new genes of the E. coli softcore genome achieving the FPE score ranges (T0, T1, T5, T10, T15, T20, T25, T30, T35, T40, T45, T50, T75, T100, T500) across the years [file 13062_2023_362_MOESM2_ESM.zip › Supplementary Figure S3.pdf]

**A**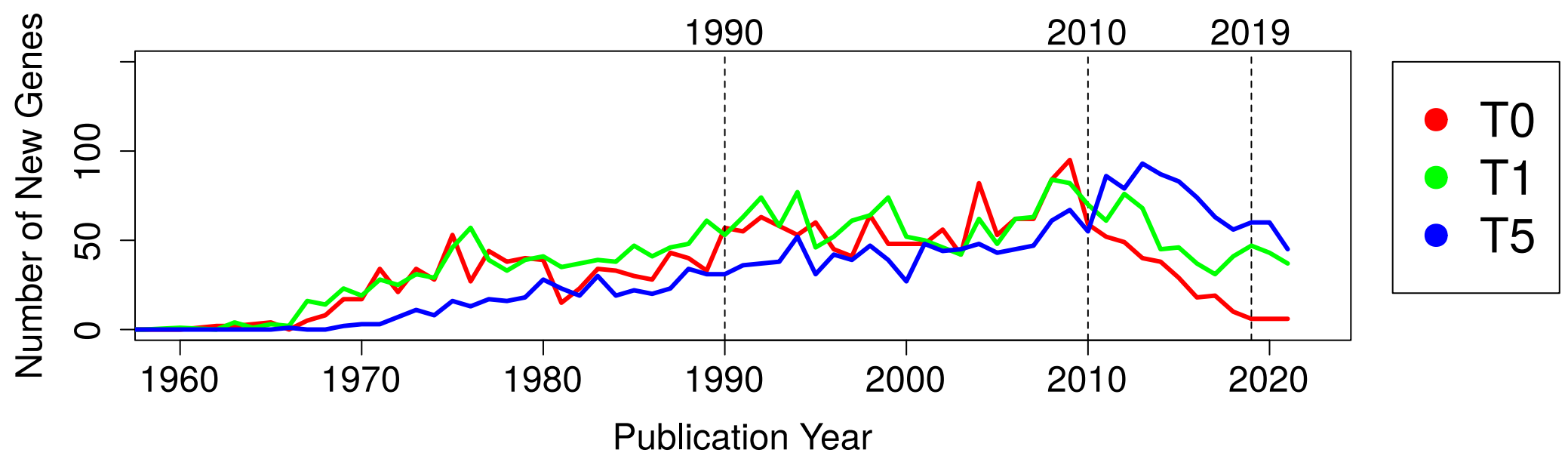**B**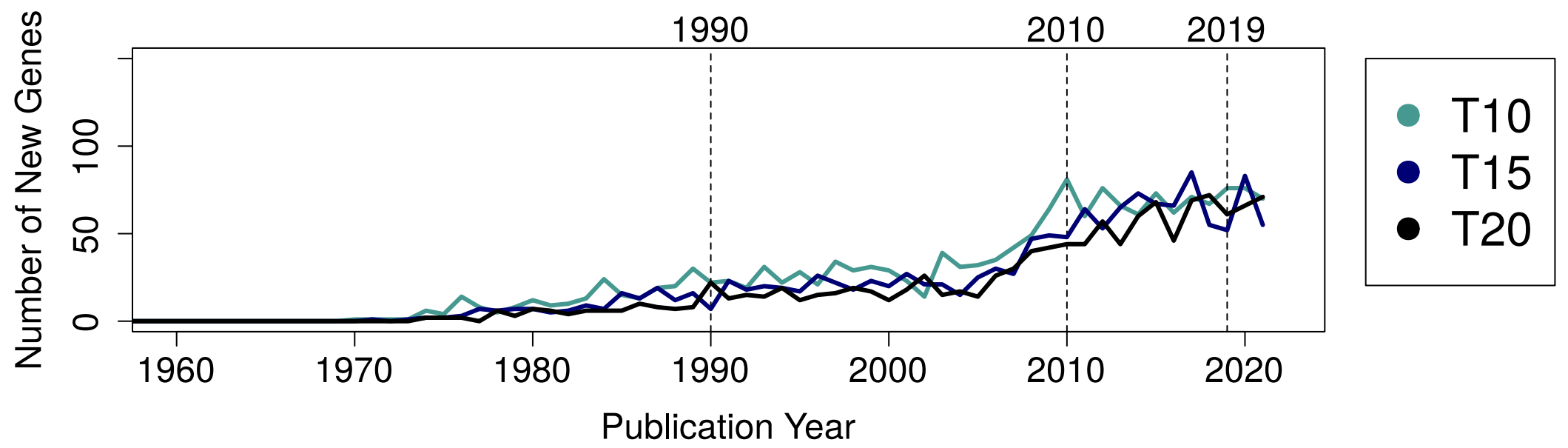**C**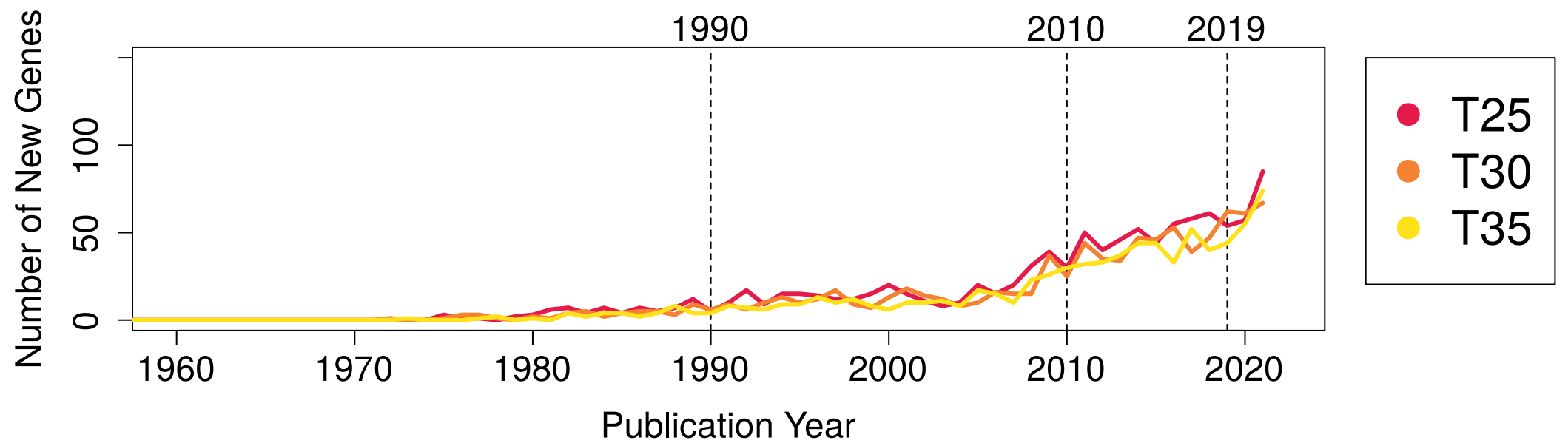**D**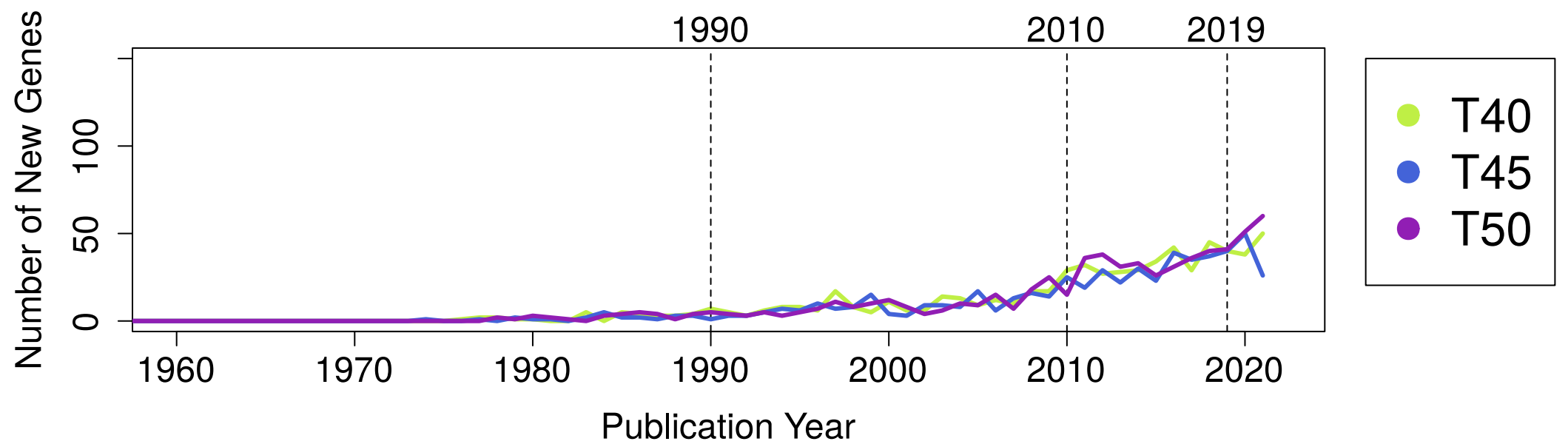**E**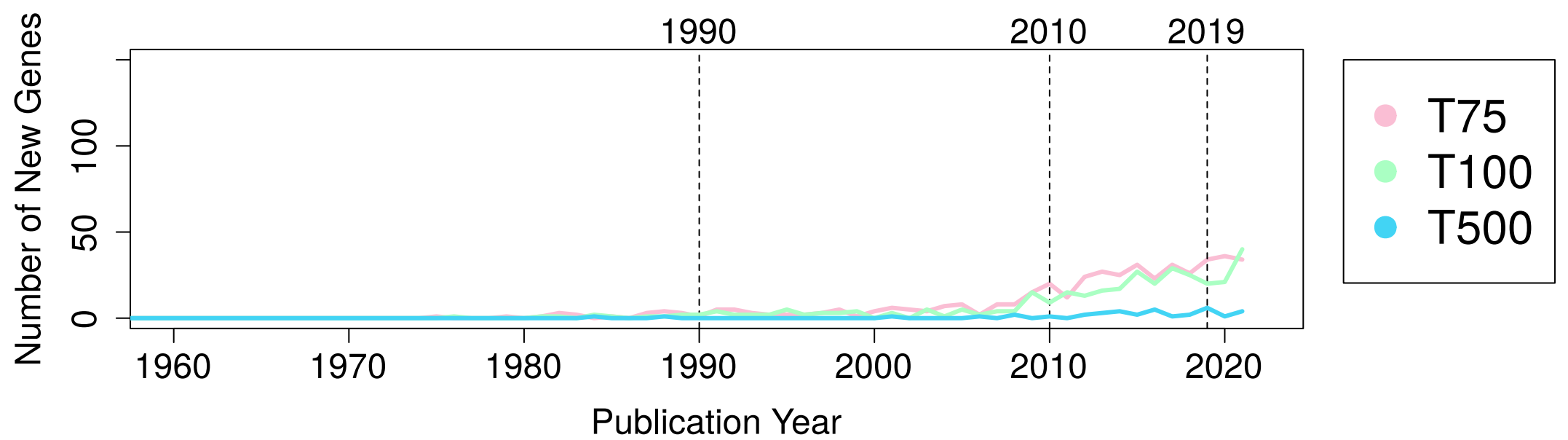

Supplement: Supplementary file 2 — Additional file 2: Fig. S1. We show the total number of E. coli softcore genes’ related publications (red line relative to the left y-axis) and the total number of genes mentioned in the respective literature (blue line relative to the right y-axis) from year 1939 up to year 2021. The blue dashed vertical lines mark the expansion period for the total number of genes from year 1965 to 2009. It apparently plateaus after the year 2019. The red dashed vertical lines at years 1970 and 2007 indicate two periods of publication dynamics: 1970–2007 and 2007–2021. The ratio of the number of publications in each year to the total number of new genes identified in each year is shown in the insert. Fig. S2. FPE plots for different FPE score ranges from year 1960 until 2021 for E. coli K-12 genes are separately shown for five different categories, i.e. (A) very understudied, (B) understudied, (C) moderately studied, (D) intensively studied and (E) very intensively studied. The y-axis is given in the same scale for visual comparison across different categories. Fig. S3. We illustrate the number of new genes of E. coli K-12 achieving the FPE score ranges (T0, T1, T5, T10, T15, T20, T25, T30, T35, T40, T45, T50, T75, T100, T500) across the years in (A) phase 1 and (B) phase 2 periods. The linear regression line (number of new genes (y-axis) versus year (x-axis)) is shown. The magnitude of the slope is provided in Table 2. Fig. S4. FPE plots for different FPE score range from year 1960 until 2021 for E. coli softcore genes are separately shown for five different categories, i.e. (A) very understudied, (B) understudied, (C) moderately studied, (D) intensively studied and (E) very intensively studied. The y-axis is given in the same scale for visual comparison across different categories. Fig. S5. We illustrate the number of new genes of the E. coli softcore genome achieving the FPE score ranges (T0, T1, T5, T10, T15, T20, T25, T30, T35, T40, T45, T50, T75, T100, T500) across the years [file 13062_2023_362_MOESM2_ESM.zip › Supplementary Figure S4.pdf]

**A**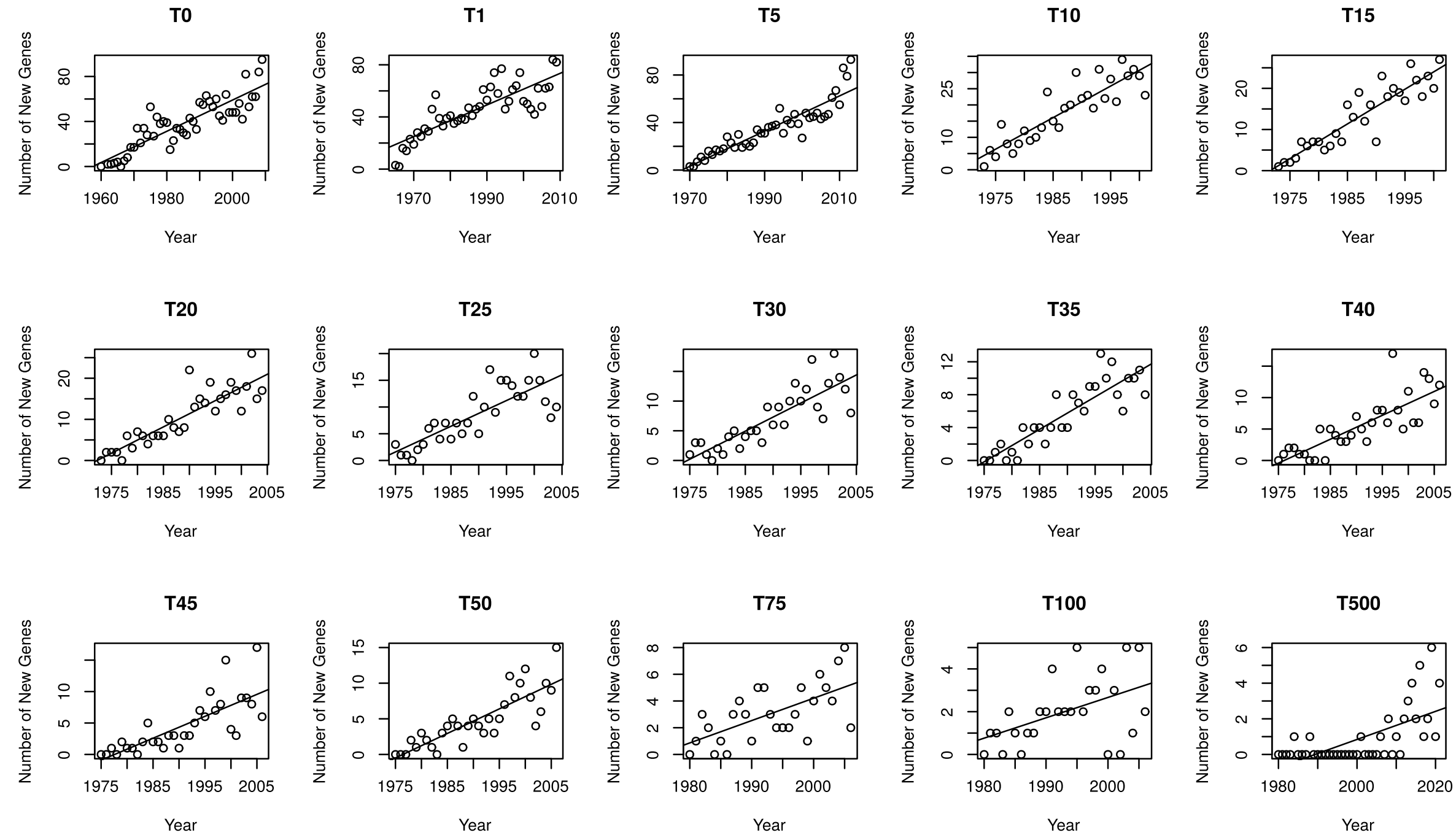**B**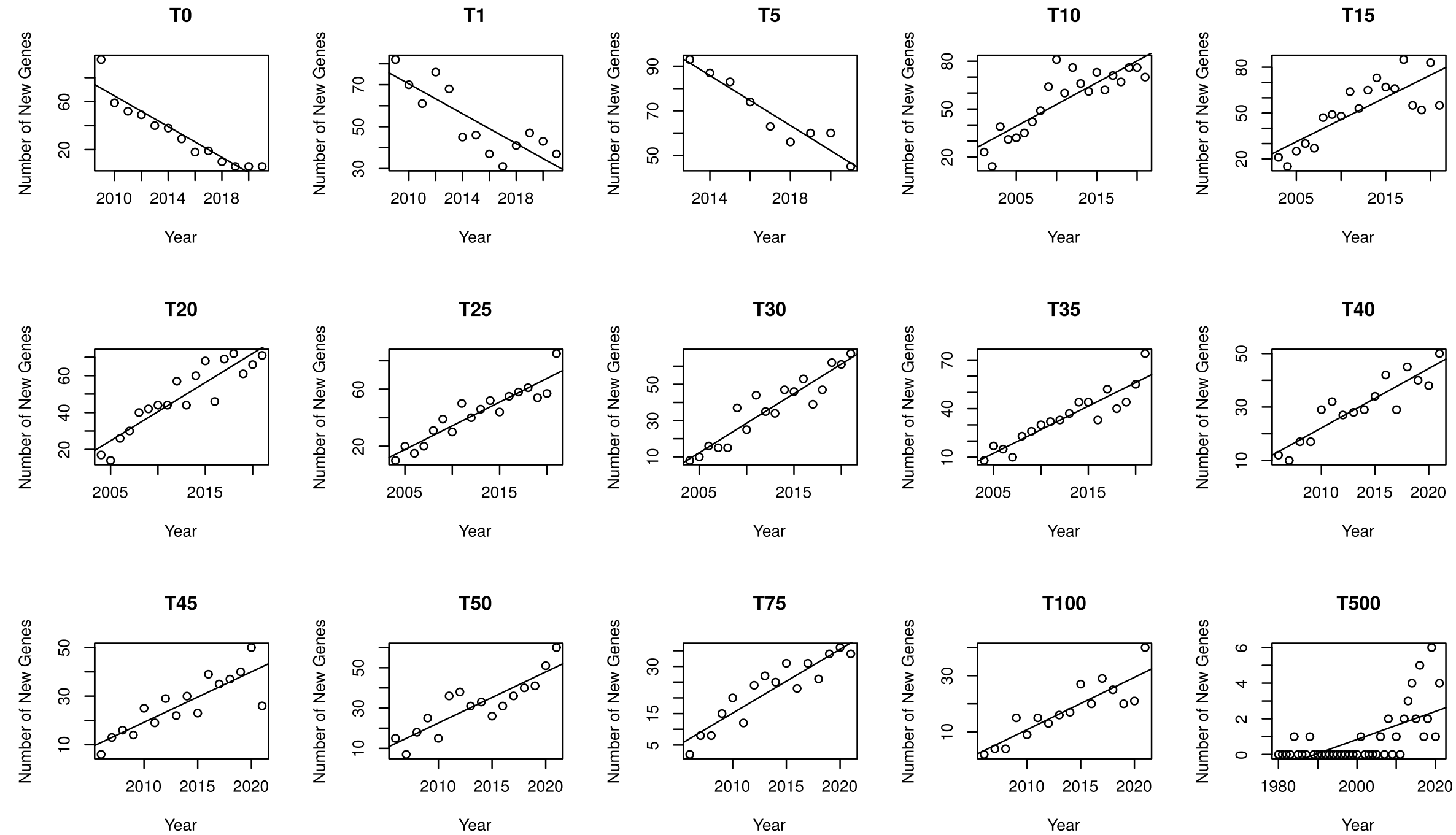

Supplement: Supplementary file 2 — Additional file 2: Fig. S1. We show the total number of E. coli softcore genes’ related publications (red line relative to the left y-axis) and the total number of genes mentioned in the respective literature (blue line relative to the right y-axis) from year 1939 up to year 2021. The blue dashed vertical lines mark the expansion period for the total number of genes from year 1965 to 2009. It apparently plateaus after the year 2019. The red dashed vertical lines at years 1970 and 2007 indicate two periods of publication dynamics: 1970–2007 and 2007–2021. The ratio of the number of publications in each year to the total number of new genes identified in each year is shown in the insert. Fig. S2. FPE plots for different FPE score ranges from year 1960 until 2021 for E. coli K-12 genes are separately shown for five different categories, i.e. (A) very understudied, (B) understudied, (C) moderately studied, (D) intensively studied and (E) very intensively studied. The y-axis is given in the same scale for visual comparison across different categories. Fig. S3. We illustrate the number of new genes of E. coli K-12 achieving the FPE score ranges (T0, T1, T5, T10, T15, T20, T25, T30, T35, T40, T45, T50, T75, T100, T500) across the years in (A) phase 1 and (B) phase 2 periods. The linear regression line (number of new genes (y-axis) versus year (x-axis)) is shown. The magnitude of the slope is provided in Table 2. Fig. S4. FPE plots for different FPE score range from year 1960 until 2021 for E. coli softcore genes are separately shown for five different categories, i.e. (A) very understudied, (B) understudied, (C) moderately studied, (D) intensively studied and (E) very intensively studied. The y-axis is given in the same scale for visual comparison across different categories. Fig. S5. We illustrate the number of new genes of the E. coli softcore genome achieving the FPE score ranges (T0, T1, T5, T10, T15, T20, T25, T30, T35, T40, T45, T50, T75, T100, T500) across the years [file 13062_2023_362_MOESM2_ESM.zip › Supplementary Figure S5.pdf]

TMHMM posterior probabilities for GF\_29643

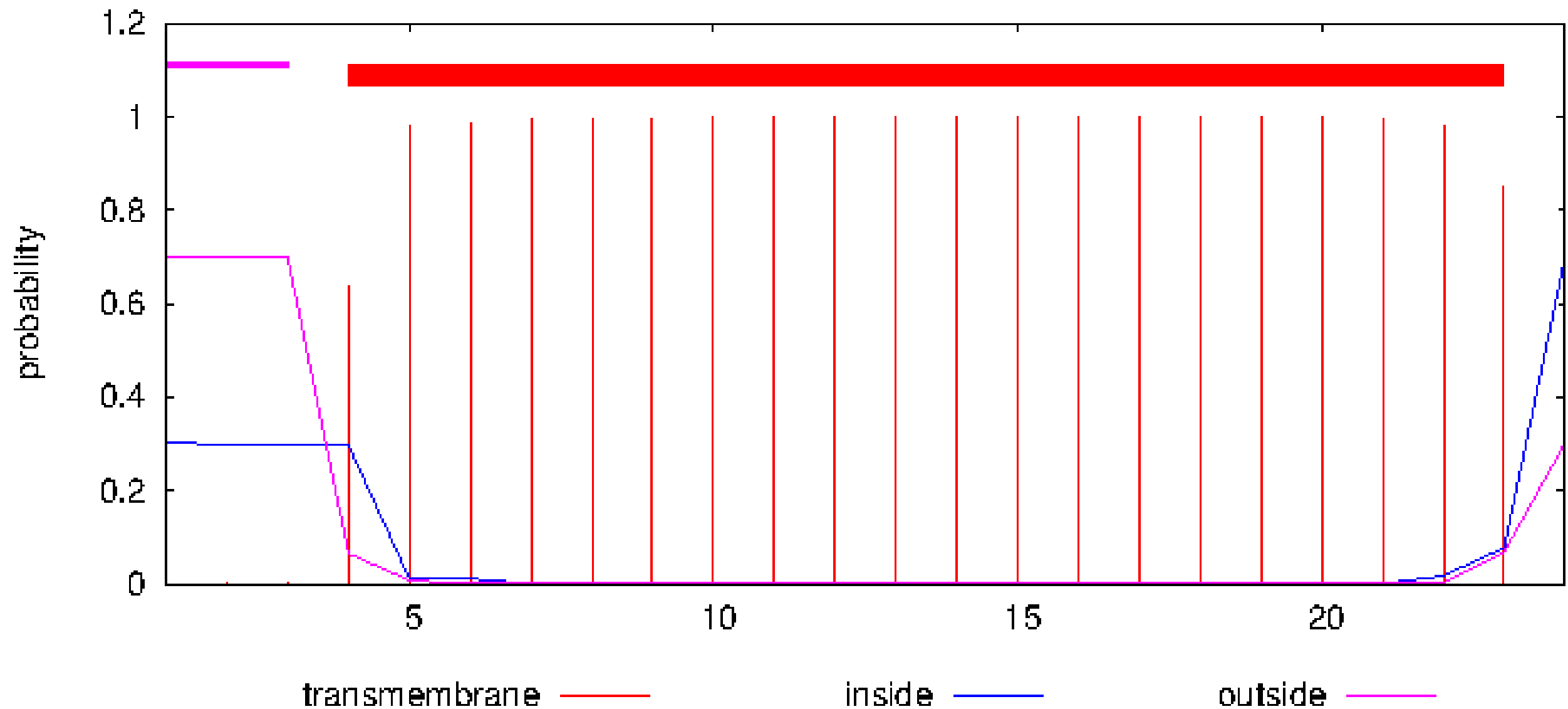

Supplement: Supplementary file 2 — Additional file 2: Fig. S1. We show the total number of E. coli softcore genes’ related publications (red line relative to the left y-axis) and the total number of genes mentioned in the respective literature (blue line relative to the right y-axis) from year 1939 up to year 2021. The blue dashed vertical lines mark the expansion period for the total number of genes from year 1965 to 2009. It apparently plateaus after the year 2019. The red dashed vertical lines at years 1970 and 2007 indicate two periods of publication dynamics: 1970–2007 and 2007–2021. The ratio of the number of publications in each year to the total number of new genes identified in each year is shown in the insert. Fig. S2. FPE plots for different FPE score ranges from year 1960 until 2021 for E. coli K-12 genes are separately shown for five different categories, i.e. (A) very understudied, (B) understudied, (C) moderately studied, (D) intensively studied and (E) very intensively studied. The y-axis is given in the same scale for visual comparison across different categories. Fig. S3. We illustrate the number of new genes of E. coli K-12 achieving the FPE score ranges (T0, T1, T5, T10, T15, T20, T25, T30, T35, T40, T45, T50, T75, T100, T500) across the years in (A) phase 1 and (B) phase 2 periods. The linear regression line (number of new genes (y-axis) versus year (x-axis)) is shown. The magnitude of the slope is provided in Table 2. Fig. S4. FPE plots for different FPE score range from year 1960 until 2021 for E. coli softcore genes are separately shown for five different categories, i.e. (A) very understudied, (B) understudied, (C) moderately studied, (D) intensively studied and (E) very intensively studied. The y-axis is given in the same scale for visual comparison across different categories. Fig. S5. We illustrate the number of new genes of the E. coli softcore genome achieving the FPE score ranges (T0, T1, T5, T10, T15, T20, T25, T30, T35, T40, T45, T50, T75, T100, T500) across the years [file 13062_2023_362_MOESM2_ESM.zip › Supplementary Figure S6.pdf]

U00096.3:325576..333459 Escherichia coli str. K-12 substr. MG1655, complete genome

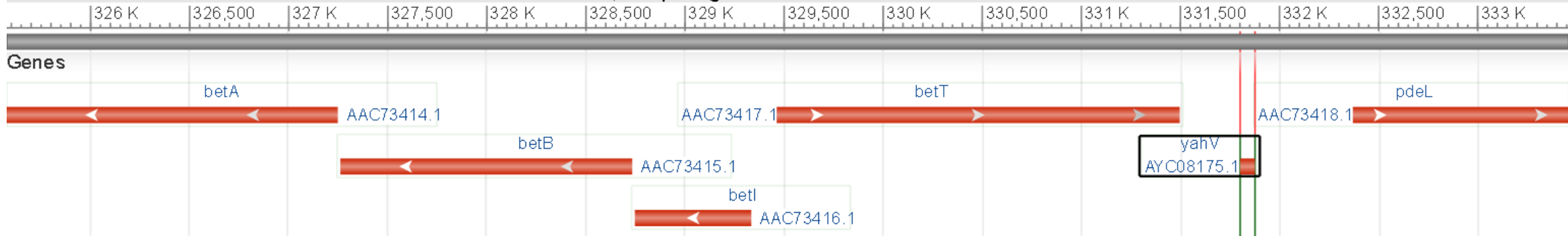

Supplement: Supplementary file 2 — Additional file 2: Fig. S1. We show the total number of E. coli softcore genes’ related publications (red line relative to the left y-axis) and the total number of genes mentioned in the respective literature (blue line relative to the right y-axis) from year 1939 up to year 2021. The blue dashed vertical lines mark the expansion period for the total number of genes from year 1965 to 2009. It apparently plateaus after the year 2019. The red dashed vertical lines at years 1970 and 2007 indicate two periods of publication dynamics: 1970–2007 and 2007–2021. The ratio of the number of publications in each year to the total number of new genes identified in each year is shown in the insert. Fig. S2. FPE plots for different FPE score ranges from year 1960 until 2021 for E. coli K-12 genes are separately shown for five different categories, i.e. (A) very understudied, (B) understudied, (C) moderately studied, (D) intensively studied and (E) very intensively studied. The y-axis is given in the same scale for visual comparison across different categories. Fig. S3. We illustrate the number of new genes of E. coli K-12 achieving the FPE score ranges (T0, T1, T5, T10, T15, T20, T25, T30, T35, T40, T45, T50, T75, T100, T500) across the years in (A) phase 1 and (B) phase 2 periods. The linear regression line (number of new genes (y-axis) versus year (x-axis)) is shown. The magnitude of the slope is provided in Table 2. Fig. S4. FPE plots for different FPE score range from year 1960 until 2021 for E. coli softcore genes are separately shown for five different categories, i.e. (A) very understudied, (B) understudied, (C) moderately studied, (D) intensively studied and (E) very intensively studied. The y-axis is given in the same scale for visual comparison across different categories. Fig. S5. We illustrate the number of new genes of the E. coli softcore genome achieving the FPE score ranges (T0, T1, T5, T10, T15, T20, T25, T30, T35, T40, T45, T50, T75, T100, T500) across the years [file 13062_2023_362_MOESM2_ESM.zip › Supplementary Figure S7.pdf]

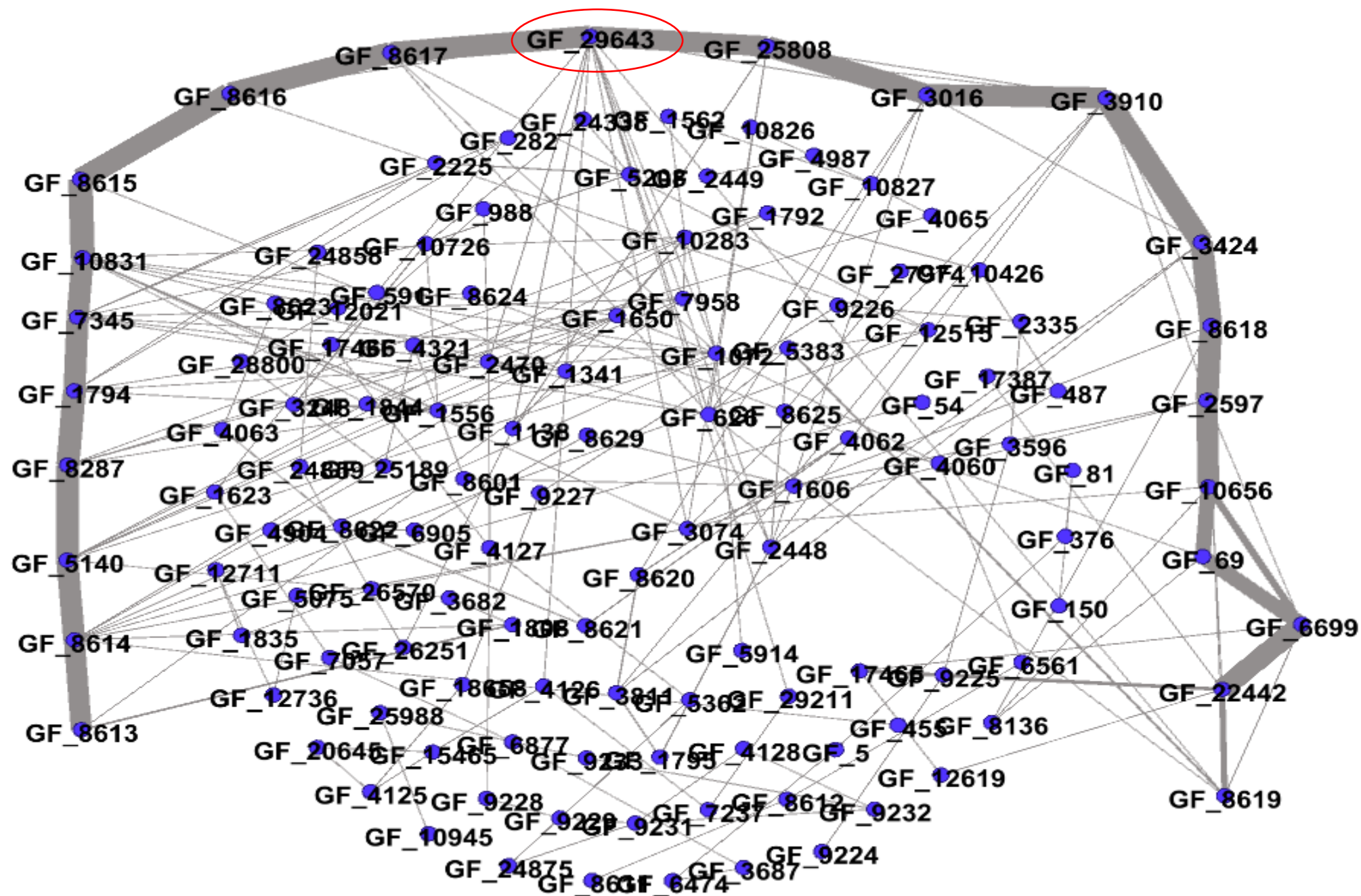

Supplement: Supplementary file 2 — Additional file 2: Fig. S1. We show the total number of E. coli softcore genes’ related publications (red line relative to the left y-axis) and the total number of genes mentioned in the respective literature (blue line relative to the right y-axis) from year 1939 up to year 2021. The blue dashed vertical lines mark the expansion period for the total number of genes from year 1965 to 2009. It apparently plateaus after the year 2019. The red dashed vertical lines at years 1970 and 2007 indicate two periods of publication dynamics: 1970–2007 and 2007–2021. The ratio of the number of publications in each year to the total number of new genes identified in each year is shown in the insert. Fig. S2. FPE plots for different FPE score ranges from year 1960 until 2021 for E. coli K-12 genes are separately shown for five different categories, i.e. (A) very understudied, (B) understudied, (C) moderately studied, (D) intensively studied and (E) very intensively studied. The y-axis is given in the same scale for visual comparison across different categories. Fig. S3. We illustrate the number of new genes of E. coli K-12 achieving the FPE score ranges (T0, T1, T5, T10, T15, T20, T25, T30, T35, T40, T45, T50, T75, T100, T500) across the years in (A) phase 1 and (B) phase 2 periods. The linear regression line (number of new genes (y-axis) versus year (x-axis)) is shown. The magnitude of the slope is provided in Table 2. Fig. S4. FPE plots for different FPE score range from year 1960 until 2021 for E. coli softcore genes are separately shown for five different categories, i.e. (A) very understudied, (B) understudied, (C) moderately studied, (D) intensively studied and (E) very intensively studied. The y-axis is given in the same scale for visual comparison across different categories. Fig. S5. We illustrate the number of new genes of the E. coli softcore genome achieving the FPE score ranges (T0, T1, T5, T10, T15, T20, T25, T30, T35, T40, T45, T50, T75, T100, T500) across the years [file 13062_2023_362_MOESM2_ESM.zip › Supplementary Figure S8.pdf]

**Legend:**

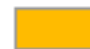

Transmembrane

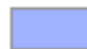

Periplasmic loop

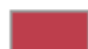

Extracellular loop

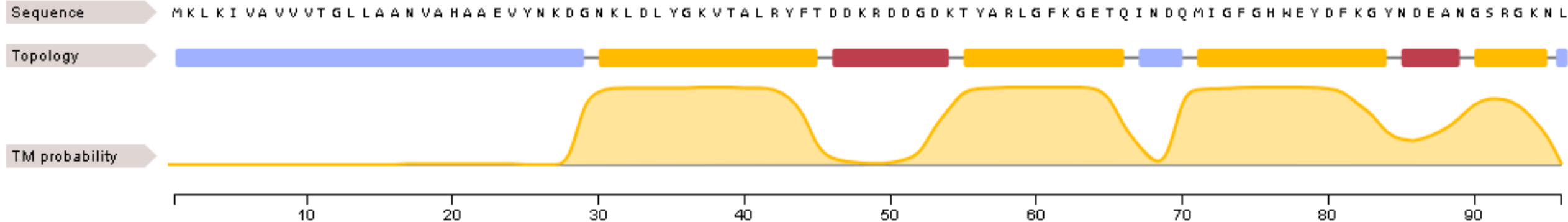

Supplement: Supplementary file 2 — Additional file 2: Fig. S1. We show the total number of E. coli softcore genes’ related publications (red line relative to the left y-axis) and the total number of genes mentioned in the respective literature (blue line relative to the right y-axis) from year 1939 up to year 2021. The blue dashed vertical lines mark the expansion period for the total number of genes from year 1965 to 2009. It apparently plateaus after the year 2019. The red dashed vertical lines at years 1970 and 2007 indicate two periods of publication dynamics: 1970–2007 and 2007–2021. The ratio of the number of publications in each year to the total number of new genes identified in each year is shown in the insert. Fig. S2. FPE plots for different FPE score ranges from year 1960 until 2021 for E. coli K-12 genes are separately shown for five different categories, i.e. (A) very understudied, (B) understudied, (C) moderately studied, (D) intensively studied and (E) very intensively studied. The y-axis is given in the same scale for visual comparison across different categories. Fig. S3. We illustrate the number of new genes of E. coli K-12 achieving the FPE score ranges (T0, T1, T5, T10, T15, T20, T25, T30, T35, T40, T45, T50, T75, T100, T500) across the years in (A) phase 1 and (B) phase 2 periods. The linear regression line (number of new genes (y-axis) versus year (x-axis)) is shown. The magnitude of the slope is provided in Table 2. Fig. S4. FPE plots for different FPE score range from year 1960 until 2021 for E. coli softcore genes are separately shown for five different categories, i.e. (A) very understudied, (B) understudied, (C) moderately studied, (D) intensively studied and (E) very intensively studied. The y-axis is given in the same scale for visual comparison across different categories. Fig. S5. We illustrate the number of new genes of the E. coli softcore genome achieving the FPE score ranges (T0, T1, T5, T10, T15, T20, T25, T30, T35, T40, T45, T50, T75, T100, T500) across the years [file 13062_2023_362_MOESM2_ESM.zip › Supplementary Figure S9.pdf]
