# Supplementary material for: About the dark corners in the gene function space of Escherichia coli remaining without illumination by scientific literature
Source: Biol Direct. 2023 Feb 28;18:7. doi: 10.1186/s13062-023-00362-0 (PMC9976479; doi:10.1186/s13062-023-00362-0)
Supplement: Supplementary file 3 — Additional file 3: File 1. Details about E. coli K-12 MG1655 genes with mapped literature FPE-scores. This file provides the list of E. coli K-12 MG1655 protein accession IDs (ProteinID) with the gene IDs (GeneID), gene names (GeneName), protein lengths (Length), product descriptions (Product), GF IDs from the E. coli pangenome study (GF_ID) and the aggregated FPE score till June 2022 for the gene ID. File 1B. Details about E. coli softcore genome GFs with mapped literature FPE-scores. This file provides the list of E. coli softcore genome GFs with gene family ID from the E. coli pangenome study (GF_ID), the representative sequence, the product description (Product), gene name (GeneName) and the aggregated FPE-score till June 2022. File 2. The mapping of E. coli K-12 MG1655 gene id (Gene) to the PubmedID, the year of publication for the respective PubmedID (Year), the number of times the gene appear in that PubmedID (Count) and the calculated FPE score (FPE_Score) for the gene id. The sum of FPE_Score for each unique PMID should be equal to 1. File 2B. The mapping of E. coli softcore gene family ID (GF_ID) to the PubmedID, the year of publication for the respective PubmedID (Year), the number of times the gene appear in that PubmedID (Count) and the calculated FPE score (FPE_Score) for the GF_ID. The sum of FPE_Score for each unique PMID should be equal to 1. File 3. This supplementary file provides the total number of E. coli K-12 MG1655 gene id that has been mentioned (#genes have been mentioned) till the specified year (Year). File 3B. This supplementary file provides the total number of E. coli softcore gene family id (GF_ID) that has been mentioned (#GF_ID have been mentioned) till the specified year (Year). File 4. This supplementary file provides the year when the E. coli K-12 MG1655 gene id (GeneID) was first mentioned in the literature. This is an approximation based on the literature mapping data. File 4B. This supplementary file provides the year when the [file 13062_2023_362_MOESM3_ESM.zip › Legends to Supplementary Files.pdf]

Supplementary File 1.txt
Details about *E. coli* K-12 MG1655 genes with mapped literature FPE-scores.

This file provides the list of *E. coli* K-12 MG1655 protein accession IDs (ProteinID) with the gene IDs (GeneID), gene names (GeneName), protein lengths (Length), product descriptions (Product), GF IDs from the *E. coli* pangenome study (GF_ID) and the aggregated FPE score till June 2022 for the gene ID.

Supplementary File 1B.txt
Details about *E. coli* softcore genome GFs with mapped literature FPE-scores.

This file provides the list of *E. coli* softcore genome GFs with gene family ID from the *E. coli* pangenome study (GF_ID), the representative sequence, the product description (Product), gene name (GeneName) and the aggregated FPE-score till June 2022.

Supplementary File 2.txt
The mapping of *E. coli* K-12 MG1655 gene id (Gene) to the PubmedID, the year of publication for the respective PubmedID (Year), the number of times the gene appear in that PubmedID (Count) and the calculated FPE score (FPE_Score) for the gene id. The sum of FPE_Score for each unique PMID should be equal to 1.

Supplementary File 2B.txt
The mapping of *E. coli* softcore gene family ID (GF_ID) to the PubmedID, the year of publication for the respective PubmedID (Year), the number of times the gene appear in that PubmedID (Count) and the calculated FPE score (FPE_Score) for the GF_ID. The sum of FPE_Score for each unique PMID should be equal to 1.

Supplementary File 3.txt
This supplementary file provides the total number of *E. coli* K-12 MG1655 gene id that has been mentioned (#genes have been mentioned) till the specified year (Year).

Supplementary File 3B.txt
This supplementary file provides the total number of *E. coli* softcore gene family id (GF_ID) that has been mentioned (#GF_ID have been mentioned) till the specified year (Year).

Supplementary File 4.txt
This supplementary file provides the year when the *E. coli* K-12 MG1655 gene id (GeneID) was first mentioned in the literature. This is an approximation based on the literature mapping data.

Supplementary File 4B.txt
This supplementary file provides the year when the *E. coli* softcore genome GF_ID (GF_ID) was first mentioned in the literature. This is an approximation based on the literature mapping data.

Supplementary File 5.txt
The data table for *E. coli* K-12 MG1655 with year of study on rows and Tx on columns. The value for each row represents the number of genes achieved Tx (T0, T1, …, T500) in the respective year.

Supplementary File 5B.txt
The data table for *E. coli* softcore genome with year of study on rows and Tx on columns. The value for each row represents the number of genes achieved Tx (T0, T1, …, T500) in the respective year.

Supplementary File 6.xlsx
Manual analysis of the lists of 176 genes (*E. coli* K-12 MG1655) and 39 GFs (95%-threshold softcore genome) without automatically assigned publications

This file (first worksheet) shows the list of 176 genes (177 transcripts due to *yibX* with two transcripts YP_010051208.1 (80AA) and YP_010051209.1 (24AA)) that do not have any literature mapped using our automated procedure. These genes/transcripts are reinvestigated by two approaches, i.e. (1) mapping onto the GFs from the previously published pangenome study and (2) manual queries on PubMed. If the gene/transcript does not have any mapped literature directly or via another gene from the respective pangenome GF after reinvestigation, it was categorized as "Unmapped".

The list is ordered according to the gene family ID (GF_ID) with its pangenome category (accessory, softcore or core), gene id (GeneID) and gene name (GeneName) information given in the table. The columns "Homologue", "Manual Check" and "Unmapped" contain the binary entry of 0 or 1 as the indicator of the reinvestigation. An entry of 1 "Manual Check" column suggests that publications relevant for the gene/transcript/protein's function can be recovered through manual checking of PUBMED. An entry of 1 in the "Homologue" column shows that one or more of the GF member genes have mapped literature from the K-12 literature (11 cases) or from the manual PUBMED searches. The value 0 indicates otherwise. Genes that cannot be mapped either through homologous mapping or manual checking will be assigned 1 in the "Unmapped" column; otherwise, it is assigned as 0.

The "Pangenome_Category" classifies the GF_ID into "accessory", "softcore" and "core" genome according to our previously published *E. coli* pangenome study. The softcore genome is defined as set of GF IDs that are present in at least 95% of the *E. coli* strains. The core genome is defined as set of GF IDs that are present in all *E. coli* strains of the *E. coli* pangenome study (using 1324 completely sequenced genomes). Any other GF ID that is found in less than 95% of the *E. coli* strains is categorized as accessory genome.

As a summary, 137 proteins encoded by the 177 transcripts from 176 genes remain unmapped. The remaining 40 cases are explained as: 31 can be assigned publications via a manual PUBMED search (Supplementary Table S1). 12 have literature-annotated homologues in their GF (11 by automated mapping of the K-12 literature, one (*ibsE*) after including manual PUBMED search results). In three cases, both conditions apply.

Thus, the 176 genes (177 transcripts/proteins) map onto 171 GFs. The 137 unmapped proteins belong to 135 GFs, out of which 25 are part of the softcore genome (one is even from the core genome) and 110 are accessory genome GFs.

Similarly, we analyzed the 39 GFs of the softcore genome (second worksheet: 39 GFs - softcore analysis, see also legend for Supplementary Table 1) that were not automatically mapped to literature. Ten of them contain K-12 genes that were annotated with articles by our manual PUBMED searches. 23 GFs coincide with GFs from the K-12 gene mapping that have no associated publication even after manual testing. Among six GFs with no K-12 gene, 3 can be mapped to multiple publications, whereas the other 3 remain unmapped. Thus, 26 GFs from the softcore genome have no assigned publication.

Supplementary File 7.xlsx

The list of 45 gene family IDs (GF_ID) that have at least one significant coincidently associated GF_ID based on the CoinFinder analysis. The number of significantly associated GF_ID (Num_AGs) is provided together with the information on how many of these significantly associated GF_ID have FPE score > 0 (Num_AGs_with_Literature) and its percentage (Num_AGs_with_Literature / Num_AGs * 100%). The total FPE score of these associated GF_IDs are given (Total_FPE). The associated gene name (GeneName) and product description (Product) are listed as well. The highlighted rows (in same color) are the clusters of GF_IDs, which share common associated GF_IDs.

Supplementary File 8.xlsx

The annotation for 30 gene family IDs (GF_ID) that are significantly associated to GF_29643, GF_4841 and GF_8394 (highlighted rows). The gene id (GeneID), gene name (GeneName), product description (Product), mapped NCBI COG reference ID (COG ID), COG Functional code, COG Functional Description and the inferred potential biological process are given. Finally, further relevant information is provided in the remarks column. The rows with red text are added because they are either softcore genes (*paaJ* and *paaY*) or borderline significantly associated (*tynA*) in the coincidence analysis. Therefore, there are 33 GFs in total in this spreadsheet.
