# Supplementary material for: About the dark corners in the gene function space of Escherichia coli remaining without illumination by scientific literature
Source: Biol Direct. 2023 Feb 28;18:7. doi: 10.1186/s13062-023-00362-0 (PMC9976479; doi:10.1186/s13062-023-00362-0)
Supplement: Supplementary file 2 — Additional file 2: Fig. S1. We show the total number of E. coli softcore genes’ related publications (red line relative to the left y-axis) and the total number of genes mentioned in the respective literature (blue line relative to the right y-axis) from year 1939 up to year 2021. The blue dashed vertical lines mark the expansion period for the total number of genes from year 1965 to 2009. It apparently plateaus after the year 2019. The red dashed vertical lines at years 1970 and 2007 indicate two periods of publication dynamics: 1970–2007 and 2007–2021. The ratio of the number of publications in each year to the total number of new genes identified in each year is shown in the insert. Fig. S2. FPE plots for different FPE score ranges from year 1960 until 2021 for E. coli K-12 genes are separately shown for five different categories, i.e. (A) very understudied, (B) understudied, (C) moderately studied, (D) intensively studied and (E) very intensively studied. The y-axis is given in the same scale for visual comparison across different categories. Fig. S3. We illustrate the number of new genes of E. coli K-12 achieving the FPE score ranges (T0, T1, T5, T10, T15, T20, T25, T30, T35, T40, T45, T50, T75, T100, T500) across the years in (A) phase 1 and (B) phase 2 periods. The linear regression line (number of new genes (y-axis) versus year (x-axis)) is shown. The magnitude of the slope is provided in Table 2. Fig. S4. FPE plots for different FPE score range from year 1960 until 2021 for E. coli softcore genes are separately shown for five different categories, i.e. (A) very understudied, (B) understudied, (C) moderately studied, (D) intensively studied and (E) very intensively studied. The y-axis is given in the same scale for visual comparison across different categories. Fig. S5. We illustrate the number of new genes of the E. coli softcore genome achieving the FPE score ranges (T0, T1, T5, T10, T15, T20, T25, T30, T35, T40, T45, T50, T75, T100, T500) across the years [file Supplementary Figure S13.pdf › Supplementary Figure S13.pdf]

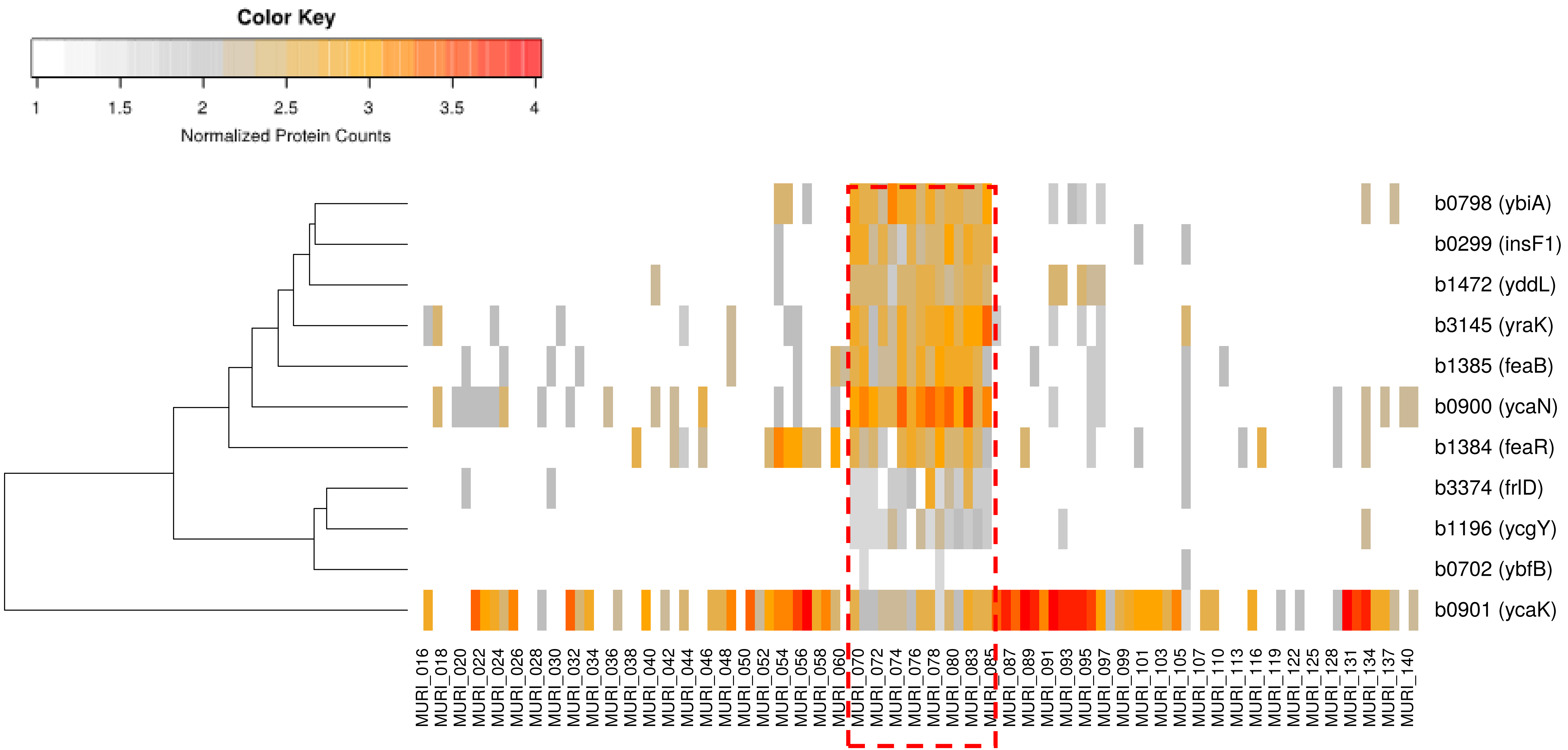
Color Key
1
1.5
2
2.5
3
3.5
4
Normalized Protein Counts
b0798 (ybiA)
b0299 (insF1)
b1472 (yddL)
b3145 (yraK)
b1385 (feaB)
b0900 (ycaN)
b1384 (feaR)
b3374 (frlD)
b1196 (ycgY)
b0702 (ybfB)
b0901 (ycaK)
MURI_016
MURI_018
MURI_020
MURI_022
MURI_024
MURI_026
MURI_028
MURI_030
MURI_032
MURI_034
MURI_036
MURI_038
MURI_040
MURI_042
MURI_044
MURI_046
MURI_048
MURI_050
MURI_052
MURI_054
MURI_056
MURI_058
MURI_060
MURI_070
MURI_072
MURI_074
MURI_076
MURI_078
MURI_080
MURI_083
MURI_085
MURI_087
MURI_089
MURI_091
MURI_093
MURI_095
MURI_097
MURI_099
MURI_101
MURI_103
MURI_105
MURI_107
MURI_110
MURI_113
MURI_116
MURI_119
MURI_122
MURI_125
MURI_128
MURI_131
MURI_134
MURI_137
MURI_140
